# The causal role of male pubertal timing for the development of externalizing and internalizing traits: results from Mendelian randomization studies

**DOI:** 10.1017/S0033291725000352

**Published:** 2025-03-28

**Authors:** Lars Dinkelbach, Triinu Peters, Corinna Grasemann, Anke Hinney, Raphael Hirtz

**Affiliations:** 1 Department of Pediatrics III, University Hospital Essen, University of Duisburg-Essen, Essen, Germany; 2 Institute of Sex- and Gender-sensitive Medicine, University Hospital Essen, University of Duisburg-Essen, Essen, Germany; 3 Section of Molecular Genetics in Mental Disorders, University Hospital Essen, University of Duisburg-Essen, Essen, Germany; 4 Center for Translational Neuro- and Behavioral Sciences, University Hospital Essen, University of Duisburg-Essen, Essen, Germany; 5 Department of Pediatrics, Division of Rare Diseases and CeSER, St. Josef-Hospital, Ruhr-University Bochum, Bochum, Germany; 6 Center for Child and Adolescent Medicine, Helios University Hospital Wuppertal, Witten/Herdecke University, Wuppertal, Germany

**Keywords:** adolescence, depression, male puberty timing, mental health

## Abstract

**Background:**

Preexisting epidemiological studies suggest that early pubertal development in males is associated with externalizing (e.g. conduct problems, risky behavior, and aggression) and internalizing (e.g. depression and anxiety) traits and disorders. However, due to problems inherent to observational studies, especially of residual confounding, it remains unclear whether these associations are causal. Mendelian randomization (MR) studies take advantage of the random allocation of genes at conception and can establish causal relationships.

**Methods:**

In this study, N = 76 independent genetic variants for male puberty timing (MPT) were derived from a large genome-wide association study (GWAS) on 205,354 participants and used as an instrumental variable in MR studies on 17 externalizing and internalizing traits and psychopathologies utilizing outcome GWAS with 16,400–1,045,957 participants.

**Results:**

In these MR studies, earlier MPT was significantly associated with higher scores for the overarching phenotype of ‘Externalizing Traits’ (b = −0.03, 95% CI [−0.06, −0.01]). However, this effect was likely driven by an earlier age at first sexual contact (b = −0.17, 95% CI [−0.21, −0.13]), without evidence for an effect on further externalizing phenotypes. Regarding internalizing phenotypes, earlier MPT was associated with higher levels of the ‘Depressed Affect’ subdomain of neuroticism (b = −0.04, 95% CI [−0.07, −0.01]). Late MPT was related to higher scores of internalizing traits in early life (b = 0.04, 95% CI [0.01, 0.08]).

**Conclusions:**

This comprehensive MR study supports a causal effect of MPT on specific traits and behaviors. However, no evidence for an effect of MPT on long-term clinical outcomes (depression, anxiety disorders, alcohol dependency, cannabis abuse) was found.

## Background

Early pubertal timing has been suggested as a risk factor for externalizing (e.g. oppositional defiant disorder, antisocial behavior, risky sexual behavior, and substance abuse) and internalizing traits and disorders (e.g. depression or anxiety) in several cross-sectional epidemiological and clinical studies with small but consistent effects in both males and females (Ullsperger & Nikolas, 2017). However, these associations are subject to numerous confounders. In boys exposed to a negative social microenvironment (e.g. parental rejection, peer victimization), early puberty is associated with increased rule-breaking behavior (Vijayakumar, Youssef, et al., 2023) and a higher burden of depressive symptoms (Benoit, Lacourse, & Claes, 2013). Additionally, adverse family environments may contribute to an earlier puberty onset (Pham, DiLalla, Corley, Dorn, & Berenbaum, 2022), introducing a potential confounding pathway. Thus, the true causal effect of earlier puberty timing on negative mental health outcomes may have been overestimated. Additional confounders of the relationship between pubertal maturation and mental health include the macroenvironment (i.e. the neighborhood income) (Niu, Sheffield, & Li, 2023), ethnicity (Hamlat, Stange, Abramson, & Alloy, 2014), and family interaction patterns (Li, Zhao, Zhang, & Zhang, 2023; Ye & Rudolph, 2023) – many of which are difficult to control in conventional observational studies. Therefore, the causal relationship between early pubertal timing and negative mental health outcomes remains unclear.

Mendelian randomization (MR) studies can overcome limitations inherent to observational studies and are able to distinguish between residual confounding and causal relationships (Ebrahim & Davey Smith, 2008; Lawlor et al., 2008). In brief, MR studies take advantage of the random allocation of genetic variants at conception. If a genetic variant is associated with an exposure (e.g. puberty timing), this genetic variant can serve as an unconfounded predictor to analyze the effect of the exposure on the outcome of interest (e.g. psychopathologies) under certain assumptions (Ebrahim & Davey Smith, 2008; Lawlor et al., 2008). Since most confounders in the relationship between puberty timing and mental health arise from environmental and social factors, which are unlikely to be linked to genetic variant allocation, the MR approach may be particularly advantageous for investigating this connection.

Several MR studies analyzed the effects of pubertal timing on psychopathologies in females and discovered an influence of early age at menarche on depression (Hirtz et al., 2022) and substance abuse (Pan et al., 2023). In males, one MR study analyzed the effect of pubertal timing on substance addiction and found no effect on tobacco, alcohol, or cannabis addiction (Pan et al., 2023). However, conclusions from this MR analysis are limited since both the exposure and outcome associations were drawn from a single sample, the UK Biobank, violating the non-sample overlap assumption of two-sample MR studies (Burgess, Davies, & Thompson, 2016). In addition, this study relied on a Genome-Wide Association Study (GWAS) assessing pubertal timing by single measures of physical maturation (such as late vs. early voice break [VB] and late vs. early facial hair growth) rather than a combined measure of male pubertal timing (Pan et al., 2023).

In males, a recent meta-analysis of GWAS based on 205,354 European males combined different indicators of pubertal timing in boys (e.g. the timing of VB and facial hair growth) to study the genetic associations of male puberty timing (MPT) in general (Hollis et al., 2020). Thus, this large-scale GWAS is less dependent on a specific assessment of MPT and provides a more reliable tool to investigate the causal effect of male pubertal timing on psychopathologies. In a previous MR study, this GWAS was utilized to analyze the effect of MPT on depression but did not find robust evidence for a relationship between both (Hirtz et al., 2023). However, further psychopathologies in males have not been addressed by MR studies so far, despite reasonable evidence for extensive associations of MPT with externalizing and internalizing traits and disorders in epidemiological studies (Ullsperger & Nikolas, 2017).

### Objective

Here, we aim to utilize multiple MR studies based on a current large GWAS to analyze whether male pubertal timing has a causal effect on the development of (1) externalizing traits, extraversion, antisocial behavior, and aggression, (2) risky behavior, including general risk tolerance, smoking, and risky sexual behavior (number of sexual partners, age at first sexual contact), (3) substance abuse, i.e. alcohol and cannabis abuse, (4) internalizing traits including neuroticism, and (5) internalizing psychiatric entities such as depression and anxiety disorders.

## Methods

### MR assumptions

To address the above-mentioned research questions, 17 two-sample MR studies were conducted. In MR studies, genetic variants or single nucleotide polymorphisms (SNPs) are used as instrumental variables to analyze the effect of the exposure variable (here, MPT) on the outcome variable (here, externalizing and internalizing traits and psychopathologies). SNPs are valid instrumental variables if the three main assumptions of MR studies are met: (a) the relevance assumption, meaning that the genetic variants have to be strongly related to the exposure, (b) the independence assumption, meaning that genetic variants are not associated with confounders of the exposure–outcome association, and (c) the exclusion restriction assumption, meaning that genetic variants are only related to the outcome via pathways mediated by the exposure (Haycock et al., 2016; Lawlor et al., 2008). In two-sample MR studies, the genotype-exposure associations and genotype-outcome associations are derived from two distinct GWAS analyses. Consequently, the two-sample MR approach relies on two additional assumptions: (e) nonoverlapping exposure and outcome GWAS, as sample overlap can result in a bias of unknown direction and extent (Burgess et al., 2016) and (f) the assumption that both the exposure and outcome GWAS are derived from the same population (Woolf et al., 2024).

### Study design and sensitivity analyses

Of the first three MR assumptions, only the relevance criterion (a) can be tested directly (Haycock et al., 2016). This assumption was ensured by calculating F-statistics for each genetic variant. SNPs with *F*-statistics < 10 are considered weak instruments (Lawlor et al., 2008). For MPT, only genome-wide significant (*p* < 5 × 10^−8^) SNPs were utilized as instrumental variables (Hollis et al., 2020), all surpassing the threshold for strong instruments (median F-statistic was 43.38, range 29.92–231.07). Violations of the second and third (b and c) assumptions arise when causal pathways between genetic variants and the outcome do not involve the exposure phenotype (i.e. the genetic variants are causally linked to the outcome either directly or via another phenotype), typically stemming from pleiotropy. Pleiotropy denotes a scenario where a genetic variant is linked to two or more seemingly unrelated phenotypes. To detect or correct violations of the second and third (b and c) assumptions, the following steps i–iii. were taken:

- Identification and exclusion of horizontally pleiotropic outliers by the MR-PRESSO framework (Verbanck, Chen, Neale, & Do, 2018). The primary endpoint for all MR analyses was the effect estimate (calculated by the inverse variance weighted [IVW] method) after the exclusion of horizontally pleiotropic outliers.
- Calculation of a set of so-called robust methods that are less susceptible to or correct for different kinds of pleiotropy (Burgess, Bowden, Fall, Ingelsson, & Thompson, 2017). As sensitivity analyses, the following set of robust methods were calculated after the exclusion of outliers: Weighted median- and mode-based estimates (Bowden, Davey Smith, Haycock, & Burgess, 2016; Hartwig, Davey Smith, & Bowden, 2017), MR Egger (Bowden, Davey Smith, & Burgess, 2015) and MR-RAPS (Zhao, Wang, Hemani, Bowden, & Small, 2020). Details on these methods are given in Supplemental Methods 1.
- Correction for pleiotropy was performed using multivariable MR (MVMR) analyses (Burgess & Thompson, 2015). Body mass index (BMI) was selected as the primary adjustment factor due to its strong genetic overlap with pubertal timing and its association with internalizing and externalizing problems, indicating a potential pleiotropic pathway (Day et al., 2015; Drosopoulou et al., 2021; Hollis et al., 2020; Hoyt, Niu, Pachucki, & Chaku, 2020). To assess whether body weight-related pleiotropy affected our results, effect estimates of MPT on psychological outcomes were calculated with adjustment for BMI (IVW BMIcorr) via MVMR. For MVMR, a GWAS on BMI in up to 374,756 males was used (Pulit et al., 2019).

Despite efforts to identify outcome GWAS without sample overlap, this study included several GWAS for relevant outcomes that used data from the UK Biobank, which also contributed to the GWAS on MPT. Thus, to assess the impact of a violation of the ‘nonoverlap assumption’ (e), beta weights and standard errors for VB were derived from 23andMe participants (Hollis et al., 2020) and used as an alternative instrumental variable to calculate an effect estimate without sample overlap (IVW VB).

To correct for multiple comparisons, the *p*-values of primary endpoints were corrected by the false discovery rate (FDR) (Benjamini & Hochberg, 1995). Scatter plots (visualizing the effect of each SNP on MPT and the outcome of interest), funnel plots (drawing the MR estimate of each SNP against the inverse of its standard error as a measure of its precision), and forest plots (illustrating the MR estimate for each SNP individually) were drawn to visually screen for violations of MR assumptions or distortion of effects by single genetic variants (Burgess et al., 2017). Leave-one-out analyses were conducted to assess whether the results were driven by single variants. Details on the software packages used can be found in the Supplemental Methods.

**Figure 1.**
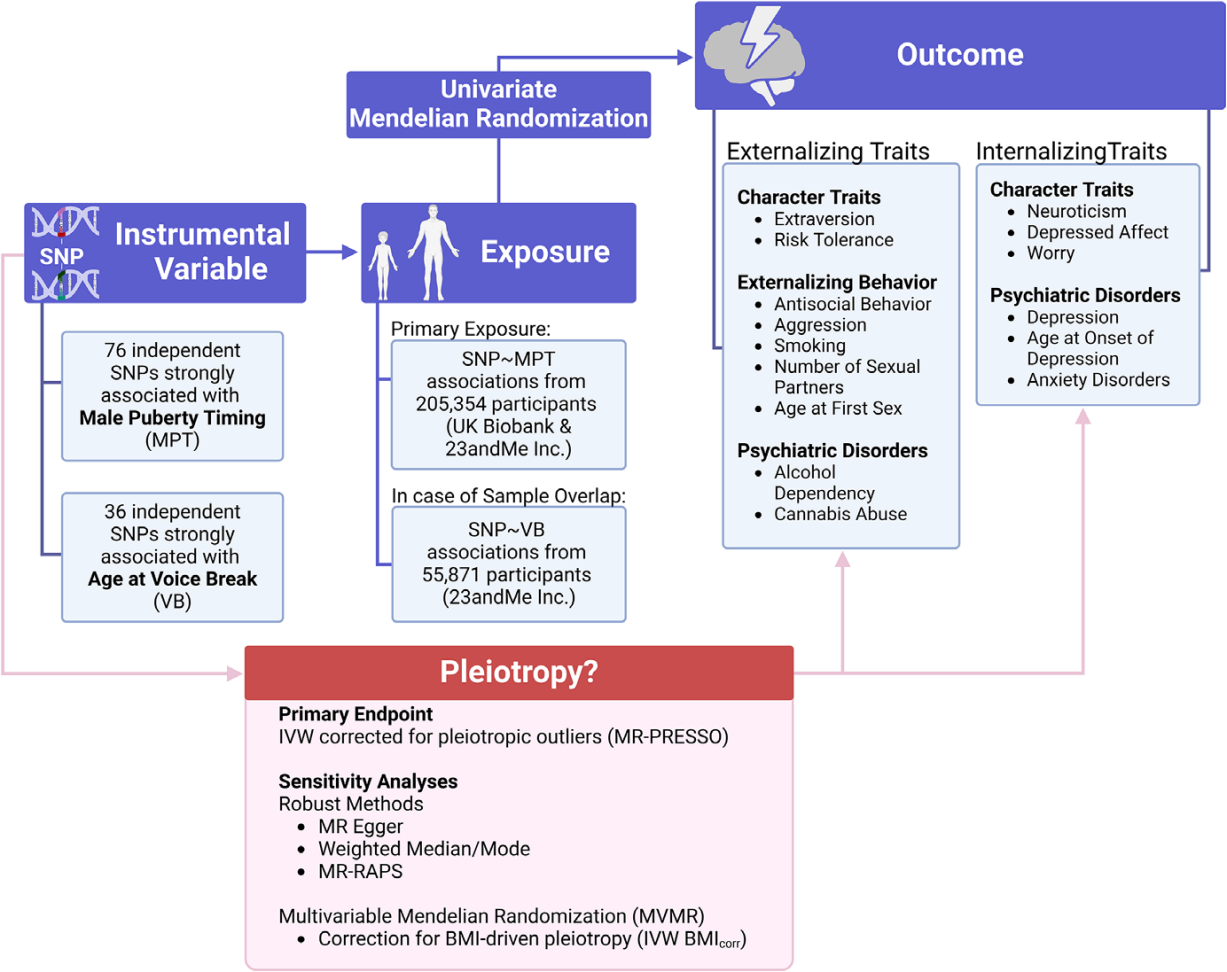
Illustration of the analytic approach and outcome variables assessed. Further details on the outcome phenotypes are given in Tables 1 and 2. BMI=body mass index; IVW=inverse-variance weighted; MPT=male puberty timing; SNP=single-nucleotide polymorphism; VB=voice break.

The MR analyses of this study were performed and reported in accordance with the STROBE-MR statement (Skrivankova et al., 2021). Figure 1 gives an overview of the study design.

### Study population

Tables 1 and 2 give an overview of the GWAS and sample sizes utilized in this study (Harder et al., 2022; Ip et al., 2021; Jami et al., 2022; Johnson et al., 2020; Karlsson Linnér et al., 2019; Karlsson Linnér et al., 2021; Mills et al., 2021; Nagel, Jansen, et al., 2018; Otowa et al., 2016; Tielbeek et al., 2017; Van den Berg et al., 2016; Walters et al., 2018; Williams et al., 2023; Wray et al., 2018). The selection of outcome GWAS was motivated by (i) the sample size and novelty of the sample, (ii) the availability of summary statistics, and (iii) the potential sample overlap between exposure and outcome GWAS. However, the latter criterion was not always met due to the excessive use of consortia data to achieve large sample sizes. Supplemental Table S1 gives a detailed description of how the respective phenotypes were obtained, the SNP-based heritability of each outcome GWAS, and the covariates included in the GWAS.

**Table 1.** Overview of the outcome GWAS included in the MR analyses for externalizing traits

Study	Trait	Phenotype description	Age group	Cohorts	Sample size
Karlsson Linnér et al., 2021 and Williams et al., 2023 ^a^	Externalizing traits	This trait is based on pooled data of GWAS (each with N > 50.000) for seven externalizing behaviors and disorders: general risk tolerance, attention-deficit/hyperactivity disorder (ADHD), problematic alcohol use, lifetime cannabis use, lifetime smoking initiation, age at first sexual contact, and number of sexual partners. Based on these data, a latent multi-trait variable, ‘Externalizing Traits’ was defined	Mainly adults	UK Biobank and 63 additional cohorts GPC (29 cohorts)	1,045,957
Van den Berg et al., 2016	Extraversion	This GWAS was based on a meta-analysis of 63,030 subjects of 29 cohorts and derived a latent variable ‘Extraversion’ based on the response behavior on extraversion-items of personality scales (e.g. the NEO Five Factor Inventory, NEO-FFI)	Mainly adults	GPC (29 cohorts)	63,030
Tielbeek et al., 2017	Antisocial behavior	The trait ‘Antisocial Behavior’ was based on measures of antisocial behavior, e.g. the clinical diagnosis of antisocial personality disorders, retrospective or current diagnosis of conduct disorders, screening tools for antisocial behavior, or teacher-based scales for rule-breaking behavior. Each of these antisocial measures was obtained in one of eight samples, and distinct GWAS were calculated for each measure and then combined via meta-analysis to the trait ‘Antisocial Behavior’	Children, adolescents, and adults	Broad Antisocial Behavior Consortium (8 cohorts)	16,400
Ip et al, 2021	Early life aggression	This trait was defined by 29 measures (self-report, parental report, or teacher ratings) covering scales of conduct problems, oppositional defiant disorders, or aggressive behavior (obtained e.g. via the Strengths and Difficulties Questionnaire, SDQ)	Children and adolescents	ACTION and EAGLE consortium (29 cohorts)	87,485
Karlsson Linnér et al., 2019	General risk tolerance	General risk tolerance was measured via a single-question item asking participants for their general comfort in taking risks	Adults	UK Biobank and 10 additional cohorts	466,571
Karlsson Linnér et al., 2019	Ever smoker	The trait ‘Ever Smoker’ was based on the participants’ self-description of being a former or current smoker	Adults	UK Biobank TAG consortium (16 cohorts)	518,633
Karlsson Linnér et al., 2019	Number of sexual partners	This trait is based on the lifetime number of sexual partners as reported by study participants on self-report	Adults	UK Biobank	370,711
Mills et al., 2021	Age at first sexual contact	This trait is based on the self-report of adult study participants on the age at which they had their first sexual contact (oral, vaginal, or anal)	Adults	UK Biobank	182,791
Walters et al., 2018	Alcohol dependency	This is a case-control GWAS where cases were defined by the diagnosis of alcohol dependency by clinicians or semi-structured interviews according to DSM-IV (or DSM-III-R) criteria	Adolescents and adults	PGC (28 cohorts)	11,569 cases 34,999 controls
Johnson et al., 2020	Cannabis abuse	This is a case-control GWAS where a case was required to meet diagnostic criteria for cannabis abuse or dependency (based on DSM-III-R, DSM-IV, DSM-V or ICD–10 codes)	Adolescents and adults	PGC (20 cohorts)	17,068 cases 357,219 controls

*Note*: This table gives an overview of the exposure and outcome GWAS included in the MR analyses in the present study. ‘Mainly adult’ indicates that most of the sample consisted of adults (see Supplemental Table S1 for details).

a In the initial GWAS by Karlsson Linnér et al. (2021) on ‘Externalizing Traits’ up to 1,373,240 participants from 65 cohorts were included. The largest cohorts came from 23andMe (*N*_max_ 599,289) and the UK Biobank (*N*_max_ 403,349). As the company 23andMe Holding Co. restricts access to the data of their samples, the GWAS was rerun based on 1,045,957 participants, excluding the 23andMe sample. This reanalysis yielded similar factor loadings and genetic correlations (Williams et al. 2023) and was used in this study for the outcome ‘Externalizing Traits’.

**Table 2.** Overview of the outcome GWAS included in the MR analyses for internalizing traits

Study	Trait	Phenotype description	Age group	Cohorts	Sample size
Jami et al, 2022	Early life internalizing traits	This trait was derived from assessments of internalizing symptoms, encompassing measures of depression, anxiety, emotional difficulties, and related internalizing issues. These assessments were based on self-ratings (19.7%) or proxy ratings (80.3%, primarily from mothers) using twelve instruments, most frequently the Strengths and Difficulties Questionnaire (SDQ) and the Achenbach System of Empirically Based Assessment (ASEBA). Data from 22 cohorts were synthesized meta-analytically to define the phenotype ‘Early Life Internalizing Traits’	Children and adolescents	EAGLE consortium (22 cohorts)	64,561^a^
Nagel et al., 2018	Neuroticism	The measurement of the personality trait ‘Neuroticism’ was based on two different questionnaires (the Eysenck Personality Questionnaire Revised Short Form (EPQ-RS) or the NEO-FFI, each containing 12 items)	Mainly adults	UK Biobank GPC (10 cohorts)	390,278
Nagel et al., 2018	Depressed affect	The traits ‘Depressed Affect’ and ‘Worry’ are subdomains of ‘Neuroticism’ and each based on four items of the EPQ-RS. These subdomains were identified by studying the genetical architecture of ‘Neuroticism’ on a single-item level (Nagel et al. 2018)	Adults	UK Biobank	357,957
Nagel et al., 2018	Worry		Adults	UK Biobank	348,219
Wray et al., 2018	Depression^b^	In this case-control GWAS, cases with ‘Depression’ were identified according to DSM-III, DSM-IV, ICD-9, or ICD-10 criteria via structured clinical interviews, checklists, or review of medical records	Adults	PGC (29 cohorts) and 4 additional cohorts	45,396 cases 97,250 controls
Harder et al., 2022	Age at onset of depression	This phenotype was based on self-reported age at diagnosis and age of onset of symptoms of major depression in participants with a lifetime history of major depression or self-reported symptoms of depression of the UK Biobank	Adults	UK Biobank	94,154
Otowa et al., 2016	Anxiety disorders	This phenotype was based on a quantitative factor score that captures different degrees of clinical severity across different anxiety disorders (including subclinical forms). The study sample was clinically enriched and included 7,016 participants with a clinical diagnosis of at least one anxiety disorder	Adults	PGC (8 cohorts)	18,186

*Note*: This table gives an overview of the exposure and outcome GWAS included in the MR analyses in the present study. ‘Mainly adult’ indicates that the majority of the sample consisted of adults (see Supplemental Table S1 for details); PGC=Psychiatric Genomics Consortium.

a The GWAS by the EAGLE (EArly Genetics and Lifecourse Epidemiology) consortium covered data from 64,561 children and adolescents from 22 cohorts (Jami et al. 2022). As participants were longitudinally assessed, the GWAS was based on 251,152 observations.

b To avoid sample overlap with the exposure GWAS, the publicly available GWAS on ‘Depression’, excluding UK Biobank participants, was used for the purpose of this study.

#### Instrumental variable

For MPT, the GWAS by Hollis et al. (2020) based on 205,354 men of European ancestry, was used. This GWAS comprised data from the UK Biobank and 23andMe in which MPT was operationalized as recalled age at VB (in the 23andMe cohort) or, for UK Biobank participants, having a relatively (compared to peers) early/late (or about average) onset of VB and onset of facial hair growth (Hollis et al., 2020). Separate GWAS for the five phenotypes (age at VB, early/late VB, and early/late facial hair growth) were calculated, standardized, and then meta-analyzed using Multi-Trait Analysis of GWAS (MTAG) to derive the composite phenotype ‘Male Puberty Timing’ (Hollis et al., 2020; Turley et al., 2018). In total, 76 independent (defined by distance of at least 1 MB) genome-wide significant (*p* < 5 × 10^−8^) target SNPs for MPT were identified and utilized as instrumental variables for the current MR analyses. These SNPs explained 2.2% of the variance in MPT. Polygenic risk scores derived from these target SNPs significantly predicted longitudinal self-reported Tanner staging in up to 2,403 boys, further supporting the validity of this operationalization of MPT (Hollis et al., 2020).

To evaluate if using UK Biobank data led to phenotype-exposure-related sample overlap that confounded our MR results, we conducted sensitivity analyses with ‘Age at VB’ data from 23andMe Inc., which included 55,871 participants (Hollis et al., 2020). Of the 76 target SNPs for MPT, 36 had F-statistics > 10 for VB had and thus qualified as instrumental variables for VB.

## Results

### The effect of MPT on externalizing traits and disorders

**Figure 2.**
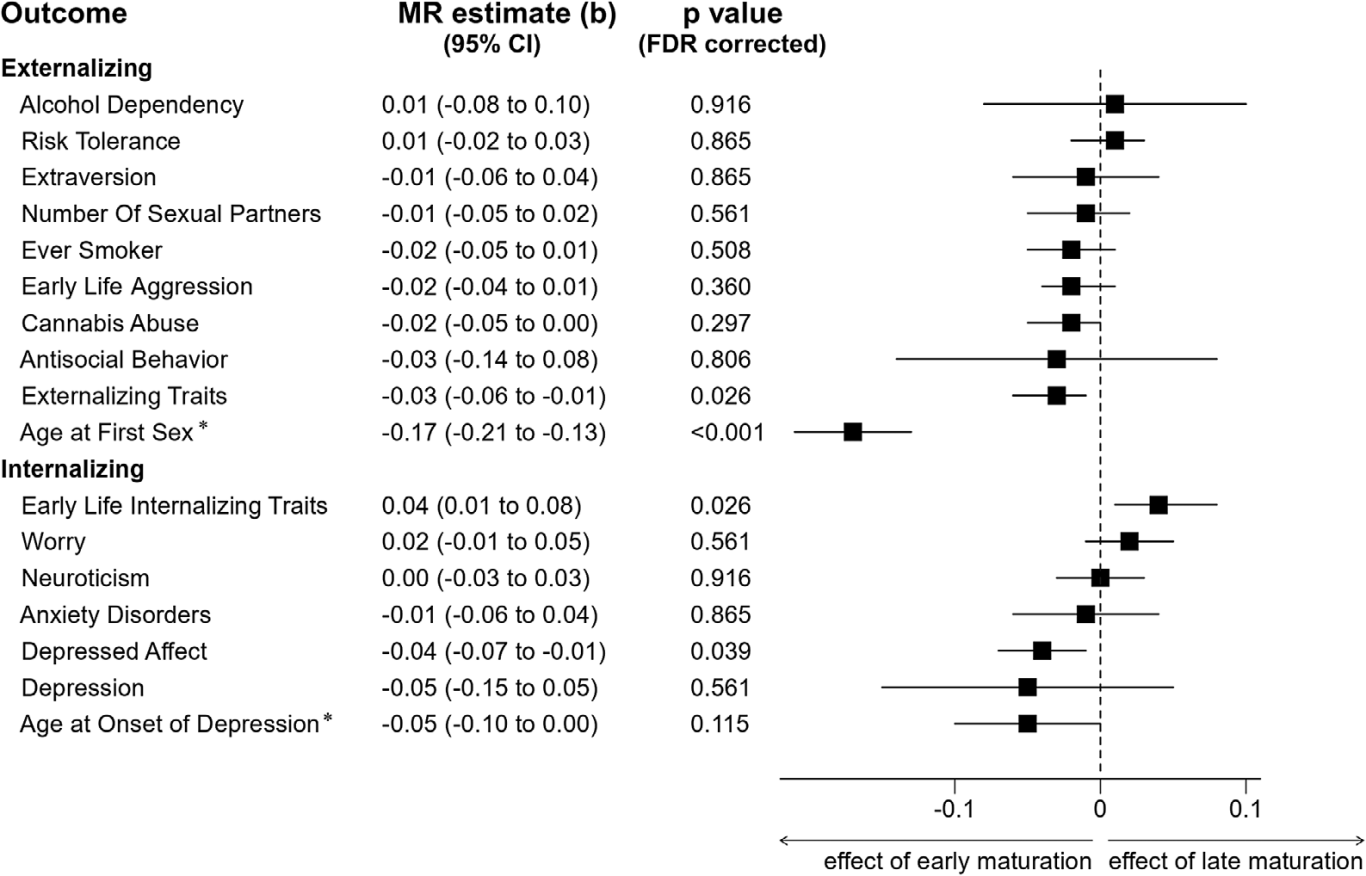
Results of the univariable Mendelian randomization (MR) analyses on the effect of male puberty timing on externalizing and internalizing traits and disorders. As primary endpoints, the inverse-variance weighted (IVW) effect estimates are illustrated after MR-PRESSO correction for pleiotropic outliers. The MR estimate (b) represents the average effect of a one-year increase in MPT on the outcome of interest. Negative effect estimates indicate a higher risk of developing psychopathology (for clinical endpoints), increased expression of a specific trait or behaviors, or a younger age at first sexual contact/depression onset with earlier male puberty timing. *For reasons of comparability, ‘Age at First Sexual Contact’ and ‘Age at Onset of Depression’ are inversely coded in the current MR analyses, e.g. negative effect estimates indicate earlier ‘Age at First Sexual Contact’ or earlier ‘Age at Onset of Depression’ with earlier MPT. The outcome variables ‘Early Life Aggression’ and ‘Early Life Internalizing Traits’ are exclusively based on data from children and adolescents, whereas the other outcomes mainly involve adult cohorts (see Tables 1 and 2 and Supplemental Table S1 for a detailed description of cohorts and phenotypes). Results of the sensitivity analyses for FDR-corrected outcomes with a *p*-value < 0.05 are illustrated in Figure 3, while those for all other outcomes are given in Supplementary Figures S21–S33.

**Figure 3.**
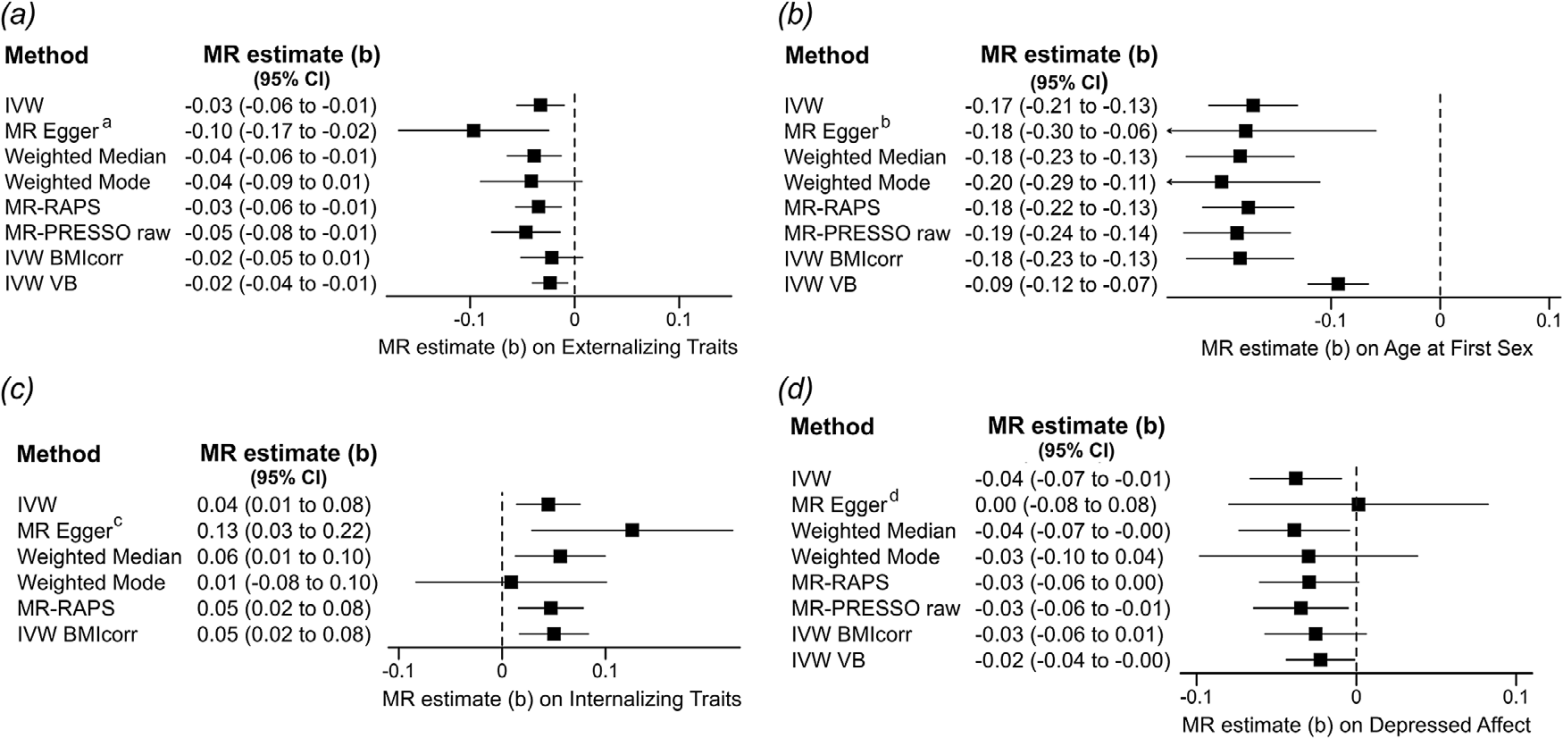
Results of the sensitivity analyses for outcomes with significant results in primary endpoints. The MR estimate (b) represents the average effect of a one-year increase in MPT per one standard deviation change in the outcome of interest. *Fig 3A:* Results of the MR analyses of the effect of male puberty timing on the outcome ‘Externalizing Traits’. For this outcome, 65 of the 76 target SNPs for MPT were available in the outcome GWAS by Williams et al. (2023). The MR-PRESSO global test for pleiotropy was significant (RSSobs=380.38, *p* < 3 × 10^−4^), and seven outliers were identified by MR-PRESSO. Thus, the instrumental variable used for further analyses consisted of 58 SNPs. *a:* Eggers-intercept did not show evidence for significant directional pleiotropy (intercept=0.0018, se=0.0010, *p* = 0.072). The *I*^2^-statistic was 0.762, indicating a possible violation of the NOME assumption. Therefore, the MR Egger result should be interpreted with caution. *Fig 3B:* MR analyses on ‘Age at First Sexual Contact’. For this outcome, all the 76 target SNPs for male puberty timing were covered in the outcome GWAS by Mills et al. (2021). The MR-PRESSO global test for pleiotropy was significant (RSSobs=241.57, *p* < 3 × 10^−4^), and MR-PRESSO identified five outliers. *b:* Eggers-intercept did not show evidence for significant directional pleiotropy (intercept=0.0002, se=0.0017, *p* = 0.905). The *I*^2^-statistic was 0.777, again indicating a possible violation of the NOME assumption. *Fig 3C:* MR analyses on ‘Early Life Internalizing Traits’. For this outcome, 71 of the 76 target SNPs for ‘Male Puberty Timing’ were available in the outcome GWAS by Jami et al. (2022). The MR-PRESSO global test for pleiotropy was not significant (RSSobs=84.24, *p* = 0.169), and MR-PRESSO identified no outlier. *c:* Eggers-intercept did not show evidence for significant directional pleiotropy (intercept=−0.002, se=0.0014, *p* = 0.089). The *I*^2^-statistic was 0.785, indicating a possible violation of the NOME assumption. *Fig 3D:* MR analyses on the ‘Depressed Affect’ subdomain of neuroticism. Concerning the ‘Depressed Affect’ subdomain of neuroticism, 72 target SNPs were included in the outcome GWAS by Nagel, Jansen, et al. (2018). The MR-PRESSO global test for pleiotropy was significant (RSSobs=140.03, *p* < 3 × 10^−4^), and one outlier was identified, which was subsequently excluded from further analyses. *d*: Eggers-intercept did not show evidence for significant directional pleiotropy (intercept=−0.0012, se=0.0012, *p* = 0.314). The *I*^2^-statistic was 0.780, indicating a possible violation of the NOME assumption for the conduction of MR Egger*. Description of effect estimates:* ‘IVW’ represents the inverse-variance weighted (IVW) effect estimate after adjusting for pleiotropic outliers identified by MR-PRESSO. The ‘MR-PRESSO raw’ estimate represents the IVW estimate without excluding pleiotropic outliers. For the outcome ‘Early Life Internalizing Traits’ no pleiotropic outlier was detected, and thus, in this instance, the ‘MR-PRESSO raw’ effect estimate corresponds to the IVW estimate and therefore is not shown. The ‘IVW BMIcorr’ estimate represents the IVW effect estimate (after exclusion of pleiotropic outliers by MR-PRESSO) of male puberty timing on the specified outcome in an MVMR analysis considering BMI as an additional exposure. The ‘IVW VB’ effect estimate is the IVW effect estimate (after the exclusion of pleiotropic outliers by MR-PRESSO) for the exposure ‘Age at Voice Break’ on the respective outcome.

For ‘Externalizing Traits’, the IVW estimate revealed a negative effect of MPT on externalizing traits, i.e. earlier pubertal timing was related to higher scores for externalizing traits (IVW, MR estimate per-one-year increase in MPT (b) = −0.03, 95% CI [−0.06, −0.01], see Figure 2). The Q-statistic indicated significant heterogeneity (based on IVW: df=57, Q-statistic=139.70, *p* = 6.78 × 10^−9^). Sensitivity analyses: The weighted median, MR-RAPS, the IVW estimate without exclusion of pleiotropic SNPs (MR-PRESSO raw), and the MR analysis without sample overlap (IVW VB) confirmed the significant results (b from −0.05 to −0.03, *p*-values from 0.002 to 0.007; see Figure 3A for details). The weighted-mode method and the MVMR considering BMI-related pleiotropy (IVW BMIcorr) led to similar effect estimates. However, wider confidence intervals resulted in nonsignificant findings (Weighted Mode, b = −0.04, 95% CI [−0.09, 0.01], *p* = 0.099; IVW BMIcorr, b = −0.02, 95% CI [−0.05, 0.01], *p* = 0.151).

For ‘Age at First Sexual Contact’, the MR analysis revealed a significant effect of MPT on the age at first sexual contact; earlier MPT was associated with an earlier age at first sexual contact (IVW, b = −0.17, 95% CI [−0.21, −0.13], see Figure 2). The Q-statistic revealed significant heterogeneity (IVW: df=70, Q-statistic=138.83, *p* = 1.9×10^−6^). All sensitivity analyses, including those addressing heterogeneity, confirmed this effect (b from −0.20 to −0.09; see Figure 3B for details).

For the remaining externalizing outcomes (‘Alcohol Dependency’, ‘Risk Tolerance’, ‘Extraversion’, ‘Cannabis Abuse’, ‘Number of Sexual Partners’, ‘Ever Smoker’, ‘Early Life Aggression’, and ‘Antisocial Behavior’), the IVW effect estimate remained nonsignificant (see Figure 2). For ‘Risk Tolerance’, ‘Number of Sexual Partners’, ‘Cannabis Abuse’, and ‘Ever Smoker’, some sensitivity analyses indicated significant effects, but these no longer appeared in the remaining or additionally performed sensitivity analyses (see Figures S23 and S25–S27 for details).

### The effect of MPT on internalizing traits and disorders

For ‘Early Life Internalizing Traits’, the MR analysis revealed a significant effect of MPT: later maturation leads to more internalizing traits in children and adolescents (IVW, b = 0.04, 95% CI [0.01, 0.08]; see Figure 2). The Q-statistic indicated no significant heterogeneity (IVW: df=70, Q-statistic=81.88, *p* = 0.157). The above finding was confirmed by all sensitivity analyses (b from 0.04 to 0.13), except for the weighted mode method (b = 0.01, 95% CI [−0.08, 0.10]; see Figure 3C).

Concerning the ‘Depressed Affect’ subdomain of neuroticism, the MR analysis revealed that earlier MPT is associated with an increased ‘Depressed Affect’ (IVW, b = −0.04, 95% CI [−0.07, −0.01]; see Figure 2). The Q-statistic was significant, indicating heterogeneity (IVW: df=70, Q-statistic=123.84, *p* = 7.6×10^−5^). Sensitivity analyses: The weighted median method, the IVW effect estimate without exclusion of outliers (MR-PRESSO raw), and the analysis excluding sample overlap (IVW VB) led to significant negative effect estimates (b from −0.04 to −0.02). The weighted mode method, MR-RAPS, and the MVMR (IVW BMIcorr) method provided similar but less precise effect estimates with wider confidence intervals and, thus, nonsignificant hypotheses testing (b ranging from −0.03 to −0.02; see Figure 3D for details). In contrast, the MR Egger method provided an effect estimate close to zero and wide confidence intervals (b = 0.00, 95% CI [−0.08, −0.08]). However, the MR Egger method might have been biased by a possible violation of the NOME assumption (*I*^2^ = 0.780; see Supplemental Methods 1 for details). The funnel plot illustrating the precision of each genetic variant and their MR effect estimate revealed one outlier with a strong negative effect estimate (see Figure S14). However, even after excluding this outlier, leave-one-out analyses confirmed an effect of MPT on ‘Depressed Affect’ (see Figure S16).

For the outcome ‘Age at Onset of Depression’, a significant effect of ‘MPT’ was observed (IVW, b = −0.05, 95% CI [−0.10, 0.0], *p* = 0.034 without FDR-correction), suggesting that earlier maturation in males is linked to a younger age at onset of depression. However, after FDR correction, this effect must be considered nonsignificant according to conventional criteria (*p* = 0.115; see Figure 2). Sensitivity analyses, including MR Egger, MR-RAPS, MR-PRESSO raw, and IVW VB, supported an association between MPT and ‘Age at Onset of Depression’. In contrast, the weighted median and weighted mode method yielded nonsignificant results with smaller (weighted median, b = −0.02, 95% CI [−0.09, 0.04]) and even positive (weighted mode, b = 0.01, 95% CI [−0.14, 0.17]) effect estimates, suggesting a potential bias of the IVW results by pleiotropy (see Figure S21 for details). Leave-one-out analyses further underscored the instability of this finding, as the exclusion of individual SNPs rendered the association between MPT and ‘Age at Onset of Depression’ nonsignificant (see Figure S20 for details).

For all other internalizing outcomes, MR analyses yielded nonsignificant results (Figure 2). This pattern was confirmed across various sensitivity analyses (Figures S22–S33).

## Discussion

Epidemiological studies indicate that an early onset of puberty is a risk factor for a broad spectrum of externalizing and internalizing behaviors and disorders. However, correlational relationships in epidemiological studies are subject to numerous confounders. Here, several MR studies were performed to provide evidence for a causal contribution of MPT to the development of character traits, behaviors, and psychopathologies. While earlier MPT led to earlier ‘Age at First Sexual Contact’, more ‘Externalizing Traits’, and increased ‘Depressed Affect’, later MPT was associated with more ‘Internalizing Traits’ in early life (childhood, adolescence, and young adulthood). However, the effect sizes for these associations were small, and all other tested associations yielded nonsignificant findings.

### Externalizing traits and disorders

There was evidence that early puberal timing in males leads to an earlier initiation of sexual activity, i.e. a younger age at first sexual contact. This observation is consistent with a genetic correlation between the age of VB and the age at first sexual contact (Mills et al., 2021) as well as increased risky sexual behaviors in early-maturing boys in epidemiological studies (Ullsperger & Nikolas, 2017). Our analyses also revealed an association between an early onset of puberty in boys and the development of externalizing traits. The outcome ’Externalizing Traits’ was operationalized via a meta-analysis that combined seven problematic behaviors and psychiatric entities (including smoking, cannabis, and problematic alcohol use, risk tolerance as well as the number of sexual partners, and the age at first sexual contact; see Supplemental Table S1 and (Karlsson Linnér et al., 2021)). However, except for the age at first sexual contact, MR analyses exploring the relationship between MPT and specific entities (‘Alcohol Dependency’, ‘Cannabis Abuse’, ‘Ever Smoker’, ‘Risk Tolerance’, and ‘Number of Sexual Partners’) by individual GWAS found no significant effects of MPT on these outcomes (see Figure 2). While an additional influence of MPT on the broader phenotype ‘Externalizing Traits’ beyond individual outcomes cannot be ruled out, the relatively larger effect size observed between MPT and ‘Age at First Sexual Contact’, compared to its effect on ‘Externalizing Traits’, suggests that the association with the combined outcome is primarily driven by its effect on age at first sexual contact. Therefore, it remains uncertain whether MPT exerts any further influence on externalizing traits beyond this specific outcome.

### Internalizing traits and disorders

To our surprise, we observed that *later* male puberty leads to more internalizing traits in children and adolescents (‘Early Life Internalizing Traits’; i.e. depressive symptoms, emotional problems, and anxiety). This observation contrasts with most previous epidemiological studies suggesting a link between *early* (but not late) onset of puberty and the development of internalizing problematic traits in adolescent boys (Ullsperger & Nikolas, 2017). Additionally, a meta-analysis of the effects of *late* puberty timing in males did not indicate an association with the occurrence of psychopathologies (Ullsperger & Nikolas, 2017). However, this conclusion rests on relatively few studies, as noted by the authors of this meta-analysis (Ullsperger & Nikolas, 2017). Further, a recent large combined epidemiological and MR study, including the most comprehensive set of confounders to date, found no evidence of a relationship between (either early or late) MPT and major depressive disorder (MDD) in males (Hirtz et al., 2023). This finding suggests that previous studies may have been affected by confounding, which could also extend to other domains of internalizing traits. The ‘gendered deviation’ hypothesis postulates that those experiencing the most extreme deviations in puberty timing are at the highest risk for mental health problems (Sontag, Graber, & Clemans, 2011; Ullsperger & Nikolas, 2017). As girls generally mature earlier than boys, late-maturing boys are particularly at risk of suffering psychosocial stress, as late-maturing boys are not only the last of their sex but also the last of their whole age group to go through puberty (Sontag et al., 2011; Ullsperger & Nikolas, 2017). So far, epidemiological studies have suggested little evidence for this hypothesis (Ullsperger & Nikolas, 2017). In contrast, our observation of more internalizing symptoms with later maturation in boys would be consistent with this hypothesis. However, it must be noted that the outcome GWAS by Jami et al. (2022) relied on a heterogeneously defined phenotype of ‘Early Life Internalizing Traits’ that was assessed using multiple sources (e.g. parental, self, or teacher ratings) and different measurement instruments, across a broad age range (boys and girls between 3 and 18 years). As individual-level data or age-/instrument-specific GWAS were not available to us, our analysis cannot disentangle whether the effect found here is age-specific (e.g. later MPT leading to more internalizing problems, early MPT leading to less internalizing problems, or both) or nonlinear (e.g. extreme early or late puberty leading to more internalizing problems). Additionally, it remains unclear whether our finding is applicable to all measurement approaches included to define the outcome phenotype. Thus, the final interpretation of our results, and the extent to which confounding factors may account for discrepancies with existing epidemiological studies, must be determined by future, well-designed epidemiological studies that are able to analyze age-specific and nonlinear effects and complement our approach.

### Long-term effects of MPT

Although numerous cross-sectional studies suggest a relationship between earlier onset of puberty and (clinical) endpoints (i.e. the development of alcohol dependency, cannabis abuse, major depression, or anxiety disorders), this was not shown by the MR analyses conducted in the present study. The clinical outcomes in this study were assessed in adults considering acute or past diagnoses or symptoms, thereby serving as an estimate of the lifetime risk for these conditions. The ‘maturational disparity’ hypothesis postulates that early maturing children and adolescents are confronted with a mismatch between rapid physical changes and slowly evolving cognitive resources to cope with these changes (Benoit et al., 2013; Ullsperger & Nikolas, 2017) which is also supported on a neurophysiological level (Vijayakumar, Whittle, & Silk, 2023). However, whether this mismatch attenuates with increasing age, and thus the long-term impact of early puberty on problematic behaviors, still needs to be investigated. In line, recent longitudinal studies have shown that the effects of early puberty in males on antisocial behavior, depression, externalizing traits, and substance abuse decrease (and in some cases even reverse) with increasing age (Dimler & Natsuaki, 2021; Hoyt et al., 2020). Accordingly, the MR analyses conducted here also showed a relationship between early onset of puberty and age at first sexual contact but not the total number of sexual partners in adulthood. Also, while there was a notable trend towards a younger age of onset of depressive disorders, this did not apply to the lifetime risk for the development of a MDD. Thus, these results indicate that mental health consequences associated with ‘maturational disparity’ are transient, possibly confined to adolescence and early adulthood. On a genetic level, a recent GWAS in 94,154 participants with depression found a significant, albeit not perfect, genetic correlation (*r*_g_ = −0.49) between age at onset of depression and lifetime MDD risk (Harder et al., 2022). Similarly, strong but not perfect correlations were found between internalizing traits in children and adolescents and the lifetime risk for depression and anxiety disorders (*r*_g_ = 0.72 and 0.76; (Jami et al., 2022). Thus, even though much of the genetic liability of psychiatric disorders is shared across the lifespan, their manifestation exhibits age-related specificity. Further GWAS focusing on specific age groups (e.g. in children, adolescents, and young adults) are needed to gain a deeper understanding of these complex relationships across the lifespan and could inform future MR studies.

The only lifetime trait affected by MPT was the ‘Depressed Affect’ subdomain of neuroticism, with early puberty timing in males leading to increased scores for ‘Depressed Affect’. It should be noted that the effect of MPT on ‘Depressed Affect’ was not consistently found in sensitivity analyses, potentially related to issues of pleiotropy. The ‘Depressed Affect’ subdomain is derived from four of twelve items of a neuroticism scale (for details, see Supplemental Table S1) by clustering genetic correlations on a single-item level (Nagel, Watanabe, Stringer, Posthuma, & Van Der Sluis, 2018). In contrast, the other subdomain of this scale, ‘Worry’, exhibited a nonsignificant effect estimate in the opposite direction in the present study. Consequently, our MR studies revealed a nonsignificant effect of MPT on the broad trait ‘Neuroticism’. Despite strong genetic correlations between ‘Depressed Affect’ and depressive symptoms as well as anxiety (Nagel, Watanabe, et al., 2018), no effect of MPT on these outcomes was seen here. Thus, further studies on the relevance of these subdomains of ‘Neuroticism’ are needed. The effect of *early* MPT on increased levels of ’Depressed Affect’ is noteworthy, given our other finding that *late* MPT is associated with more pronounced ’Early Life Internalizing Traits,’ suggesting age-specific effects of MPT on internalizing traits. Similarly, sex differences in internalizing psychopathologies, such as depression and anxiety disorders, emerge only during adolescence (Dalsgaard et al., 2020), indicating that the causal pathways for prepubertal versus postpubertal onset may differ, with hormonal influences or puberty timing being potential contributing factors (Naninck, Lucassen, & Bakker, 2011; Rutter, Caspi, & Moffitt, 2003).

### Limitations

Our study has several limitations, some of which are inherent to the MR approach. Therefore, our results should be viewed as complementary to existing evidence from observational studies. First, MR studies are subject to the risk of horizontal pleiotropy. Although we conducted sensitivity analyses using multiple approaches to detect and correct for pleiotropic effects, including robust methods such as MR-Egger, MR-PRESSO, weighted median/mode, and multivariable MR analyses, it is not possible to eliminate the risk of undetected pleiotropic pathways. Nonetheless, the consistency of findings across various approaches strengthens the validity of our conclusions.

Second, the exposure (MPT) was determined based on self-reported information on the timing of voice breakage and facial hair growth, which might be subject to recall bias.

Third, GWAS for 16 of the 17 outcomes analyzed in this study were based on sex-pooled effect estimates rather than sex-specific analyses, primarily because sex-specific GWAS were not available. Current evidence suggests marginal sex differences in the autosomal genetic architecture of most traits, behaviors, and psychopathologies (Martin et al., 2021), justifying their use as outcomes in MR with sex-specific exposure. Accordingly, this approach has been applied in various MR studies, including testosterone levels in men or age at menarche concerning MDD (Hirtz et al., 2022; Syed, He, & Shi, 2020). Although we do not expect significant differences based on sex, given the genetic similarity of the traits and the inclusion of sex as a covariate in the outcome GWAS (see Supplemental Table S1), future research using sex-specific GWAS may help to further refine our understanding of any potential sex-specific effects.

Fourth, the sample sizes of the outcome GWAS varied widely, leading to large differences in the power of the conducted MR studies. As most of the outcome GWAS were based on continuous outcomes and the power estimation of MR studies on continuous outcomes is dependent on further information, e.g. on the exposure–outcome association on the phenotypic level (Brion, Shakhbazov, & Visscher, 2013), which was not available to us, we decided against power calculations based on arbitrary assumptions. However, despite reasonable sample sizes in most of the studies included, minor effects of MPT may have been overlooked, particularly when less reliable outcome measures were considered, such as individual rather than composite outcome phenotypes.

Fifth, two-sample MR studies as utilized here are based on linear models and, therefore, do not account for potential nonlinear relationships.

Sixth, the MR assumption that the exposure influences the outcome, rather than vice versa, could not be directly assessed. Although a reverse relationship appears unlikely, given that most outcome phenotypes were measured in adulthood, bidirectional MR studies to confirm this could not be performed due to restricted access to the summary statistics from the GWAS on MPT.

Seventh, all participants of the GWAS utilized in this study were of white European ancestry. The focus on this population was driven by the higher availability of data rather than scientific interest. However, it remains unclear whether the results of our study are transferable to populations of other ancestry.

## Conclusions

The MR analyses conducted here support a causal effect of MPT on the development of specific psychological traits and behaviors. Specifically, earlier MPT was associated with an earlier age at first sexual contact, while later pubertal timing was associated with increased internalizing traits in children and adolescents. However, all effect sizes for these effects were small. Further, the effects of MPT might be transient as no effects on adulthood extraversion or neuroticism or lifetime risk for psychiatric disorders were found. In line with this conclusion, there was a notable trend towards earlier age at the onset of depression with early maturation. However, this trend did not perpetuate to the lifetime risk of developing a MDD. To further explore the causal relationships between puberty timing and the onset of psychopathologies, particularly during the critical periods of late adolescence and early adulthood, additional age- and sex-specific GWAS followed by MR analyses are needed. MR analyses should, wherever possible, be combined with observational studies to compensate for the advantages and limitations of each of these analytical approaches.

## Supporting information

Dinkelbach et al. supplementary material 1 Dinkelbach et al. supplementary material

Dinkelbach et al. supplementary material 2 Dinkelbach et al. supplementary material

## Data Availability

All GWAS summary statistics utilized in this study were publicly available or available upon request (see Supplemental Table S1 details on how the GWAS summary statistics were obtained).

## Abbreviations

BMI: body mass index
FDR: false discovery rate
GWAS: genome-wide association study
IVW: inverse-variance weighted
MDD: major depressive disorder
MPT: male puberty timing
MR: Mendelian randomization
MVMR: multivariable Mendelian randomization
SNP: single-nucleotide polymorphism

## Supplementary material

The supplementary material for this article can be found at http://doi.org/10.1017/S0033291725000352.
