# Supplementary material for: The causal role of male pubertal timing for the development of externalizing and internalizing traits: results from Mendelian randomization studies
Source: Psychol Med. 2025 Mar 28;55:e101. doi: 10.1017/S0033291725000352 (PMC12094630; doi:10.1017/S0033291725000352)
Supplement: Dinkelbach et al. supplementary material 1 — Dinkelbach et al. supplementary material [file S0033291725000352sup001.docx]

| **Supplemental Table S01.** Detailed description of GWAS used in MR studies for outcome phenotypes. | | | | | | | |
| --- | --- | --- | --- | --- | --- | --- | --- |
| **Study** | **Trait** | **Phenotype Description** | **Cohorts** | **Age Distribution** | **SNP-based Heritability** | **Covariates** | **GWAS Access** |
| **Externalizing Traits** | | | | | | | |
| Karlsson Linnér et al., 2021 and Williams et al., 2023 | Externalizing Traits | The outcome 'Externalizing Traits' was defined by a latent multitrait variable for self-regulation and addiction based on seven externalizing behaviors and disorders: general risk tolerance, attention-deficit/hyperactivity disorder (ADHD), problematic alcohol use, lifetime cannabis use, lifetime smoking initiation, age at first sexual contact (inversely coded), and number of sexual partners. | General risk tolerance: 11 SSGAC cohorts (incl. UK Biobank), ADHD: 11 PGC cohorts, problematic alcohol use: 24 PGC cohorts on alcohol dependence and UK Biobank on AUDIT-P, lifetime cannabis use: 15 ICC study cohorts (incl. UK Biobank), Lifetime smoking initiation: 24 GSCAN study cohorts (incl. UK Biobank), Age at first Sexual Contact: UK Biobank, Number of Sexual Partners: UK Biobank | The GWAS on ADHD was based on children, adolescents, and adults. All other traits were based on adult cases. | Reported heritability varied from 5.3% (for general risk tolerance) to 26% (for ADHD). | Sex, age, genetic principal components, genotyping array, and batch (baseline model). | GSEM.GWAS.EXTERNALIZING.excl23andMe.SHARE.v20230628.txt https://externalizing.rutgers.edu/request-data/. |
| Van den Berg et al., 2016 | Extraversion | The latent variable 'Extraversion' was based on the results of all extraversion items available for each individual in the GWAS. Extraversion items were derived from several personality inventories (from Van den Berg et al., 2016, Supplemental Material, page 15): the Neuroticism Extraversion Openness to Experience personality inventory revised (NEO-PI-R; 240 items, 48 items for Extraversion), the abbreviated NEO Five Factor Inventory (NEO-FFI; 60 items, 12 Extraversion items), the 30-item short version of the NEO-Five-Factor Model (NEO-FFI-30), the Eysenck Personality Questionnaire Revised (EPQ-R; 48 items, 12 items for Extraversion), the Junior EPQ (JEPQ; 81 items, 24 Extraversion items), the Eysenck Personality Inventory (EPI; 20 items, 9 for Extraversion), the EPI-based Amsterdamse Biografische Vragenlijst (ABV; 21 Extraversion items), the Big Five item set of the International Personality Item Pool (IPIP; 50 items, 10 items for Extraversion), the Temperament and Character Inventory version 9 (TCI; 240 items, of which 7-13 items were selected for Extraversion) and the Multidimensional Personality Questionnaire (MPQ; 198 items, of which 72 items were selected for Extraversion). | 29 study cohorts (excl. UK Biobank) | Of the 29 cohorts, 4 cohorts (ALSPAC, NTR, YOUNG FINNS, QIMR adolescents) included children and/or adolescents, the remaining cohorts were based on adults. | 5.0% | Sex, age, and, if necessary, within a particular cohort, ancestry principal components. | GPC-2.EXTRAVERSION.full, as accessed via the GWAS catalog (GCST003120). |
| Tielbeek et al., 2017 | Antisocial Behavior | The construct 'Broad Antisocial Behavior' was based on clinical diagnostic criteria based on DSM-IV (current antisocial personality disorder, retrospective or current conduct disorder), parental report on childrens' antisocial behavior as assessed by the Child Behaviour Checklist and parental report in the Development and Wellbeing Assessment (DAWBA), teacher report on the Rule-Breaking Behavior scale of the Teacher Report Form, and the Antisocial Process Screening device (APSD) in adolescents. | Eight cohorts were included: ALSPAC cohort (N= 4336), COGA cohort (N= 1379), GenerationR study (N= 1420), TEDS cohort (N= 2734), QIMR Berghofer cohort (N= 6531), Finnish CRIME Study (N= 6350), Michigan State University Twin Registry (MSUTR, N= 825), Yale-Penn cohort (N= 2316). | Four cohorts were based on children and adolescents (mean age in years; ALSPAC=13.1, GENR=6.7, TEDS=12.5, MSUTR=8.2), four on adult samples (COGA=43.8, QIMR=33.8, Finnish Crime Study56.1, Yale-Penn=41.0). | 5.2% | Sex, age, and four principal components. | GCST004954 as accessed via the GWAS catalog (28979981-GCST004954-EFO_0007052.h.tsv). |
| Ip et al, 2021^b^ | Early Life Aggression | Childhood aggression was based on 26 different instruments including self-reports, parental rating, or teacher rating. Most commonly, the Strengths and Difficulties Questionnaire (34%) and the Achenbach System of Empirically Based Assessment (ASEBA; 41%) was used. For details on the specific instruments used, see Supplemental Table 3 of Ip et al., 2021. | 29 study cohorts (excl. UK Biobank) | Children and Adolescents 1.5- to 18-year-old at time of assessment (no details on age distribution given for the combined sample). | 3.1% | Sex, age, and first five ancestry-based principal components as covariates, and, if necessary, cohort-specific covariates. | CLEANED.GWAMA_ALL.N_weighted_GWAMA.results.txt as accessed via the EAGLE consortium website (https://www.eagle-consortium.org/downloads/). |
| Karlsson Linnér et al., 2019 | General Risk Tolerance | In the UK Biobank, the general risk tolerance was evaluated with a single item: "“Would you describe yourself as someone who takes risks? Yes /No.”. The items used in the ten replication samples differed, but all used a single question to evaluate the participants (un-)comfort with taking risks. | UK Biobank (N=431,126) and ten replication samples (N=35,445). Please note that the original publication utilized data of a 23andMe sample. However, this data was not included in the published summary statistics. | Adults | 4.5% | for UK Biobank: 20 genetic principal components, sex-specific birth year fixed effects, fixed effects for genotyping batches. Covariates of the remaining samples differed, however all at least included sex, birth year, and 10 genetic principal components. | RISK_GWAS_MA_UKB+replication.txt as accessed via the GWAS catalog (GCST007322). |
| Karlsson Linnér et al., 2019 | Ever Smoker | The trait ‘Ever Smoker’ was based on the participants’ self-description of being a former or current smoker. | UK Biobank (N=444,598) and TAG consortium (16 studies, N=74,035) | Adults | 10.9% | for UK Biobank: 20 genetic principal components, sex-specific birth year fixed effects, fixed effects for genotyping batches. Covariates of the remaining samples differed, however all at least included sex, birth year, and 10 genetic principal components. | EVER_SMOKER_GWAS_MA_UKB+TAG.txt as accessed via the GWAS catalog (GCST007327). |
| Karlsson Linnér et al., 2019 | Number of Sexual Partners | This trait is based on the lifetime number of sexual partners as reported by study participants on self-report. | UK Biobank | Adults | 12.8% | for UK Biobank: 20 genetic principal components, sex-specific birth year fixed effects, fixed effects for genotyping batches. Covariates of the remaining samples differed, however all at least included sex, birth year, and 10 genetic principal components. | NUMBER_SEXUAL_PARTNERS_GWAS.txt as accessed via the GWAS catalog (GCST007326). |
| Mills at al., 2021 | Age at First Sexual Contact | The age at first sexual contact was assessed by questions such as "What was your age when you first had sexual intercourse? (Sexual intercourse includes vaginal, oral or anal intercourse)" For the GWAS by Mills et al., 2021, answers indicating an age below 12 years were excluded from further analyses. For the current Mendelian Randomization analyses, this outcome was inversely coded. | UK Biobank | Adults (mean age 57 years, SD=8.5). | SNP-based heritability varied with the participants' birth year between 15% and 23%. | First ten genetic principal components, sex and year of birth, local district at birth (baseline model), additionally number of siblings (final model). | GCST90000046_buildGRCh37.tsv.gz as accessed by the GWAS catalog. |
| Walters et al., 2018 | Alcohol Dependency | Participants were defined as suffering from alcohol dependency if they met the criteria for alcohol dependency according to the DSM-IV (or DSM-III-R in one cohort). The diagnoses were made by clinicians or semi-structured interviews (depending on the study cohort). | 28 study cohorts (excl. UK Biobank). | Four of the 28 samples included adolescents (the cohort from the Center on Antisocial Drug Dependence (CADD), the CEDAR sample, the Add Health cohort and the GEDI sample). The remaining participants were adults. | 9% | Sex and principal components for population structure. | pgc_alcdep.eur_discovery.aug2018_release.txt.gz as accessed via the website of the PGC (https://pgc.unc.edu/for-researchers/download-results/). |
| Johnson et al., 2020 | Cannabis Abuse | This is a case-control GWAS where a case met diagnostic criteria for cannabis abuse or dependency (based on DSM-III-R, DSM-IV, DSM-V or ICD-10 codes). Sole cannabis exposure did not qualify as a case. Different diagnostic interviews were used in the study cohorts (see Supplementary Appendix of Johnson et al., 2020). | 20 study cohorts (excl. UK Biobank). | Four of the twenty samples included adolsecents:, the cohort from the Center on Antisocial Drug Dependence (CADD), was based on adolescents (N=397 cases and N=699 controls, mean age 16.5 years, SD=1.4), the CEDAR sample (N=64 cases and N=148 controls, age 14-18 years), the GEDI sample (N=81 cases and N=491 controls), and the Add Health cohort (N=847 cases and N=4839 controls). The remaining participants were adults. | Depending on the estimated population prevalence of this phenotype, SNP-based heritability was reported to be 6.7%-12.1%. | Sex and five to ten within-ancestry principal components (depending on the study cohort). Age was not included as this variable was not available in all cohorts. | CUD_EUR_full_public_11.14.2020.gz as accessed via the website of the PGC (https://pgc.unc.edu/for-researchers/download-results/). |
| **Internalizing Traits** | | | | | | | |
| Jami et al, 2022 | Early Life Internalizing Traits | Twelve instruments were used to measure internalizing symptoms, which included measures of Depression, Anxiety, Emotional Problems and Internalizing Problems. Of these, the Emotional Problems Subscale of the Strengths and Difficulties Questionnaire (SDQ, 38.2%), the 'Internalizing Problems' subscale of the Achenbach System of Empirically Based Assessment (ASEBA, 36.7%), and the 'Internalizing' scale of the Rutter Children’s Questionnaires (8.2%) were most often used. Ratings were based on observations by mothers (40.7%), fathers (6.8%), teachers (18.3%), self (19.7%), and siblings (0.7%). (From Jami et al, 2022, page 936) | 22 study cohorts (excl. UK Biobank) | 251,152 observations were included in the GWAS. Of these, 30.0% were in adolescence (13-18yrs), 18.4% in late childhood (11-12yrs), 36% in mid childhood (7-10yrs) and 15.1% in early childhood (3-6 years). | 1.7% | Sex, age, first five ancestry-based principal components, and, if necessary, cohort-specific covariates. | GCST90054778_buildGRCh37.tsv.gz as accessed via the GWAS catalog. |
| Nagel et al., 2018 | Neuroticism | The personality trait neuroticism was measured via questionnaires: the Eysenck Personality Questionnaire Revised Short Form (EPQ-RS, 12 items) in the UK Biobank and the NEO-FFI (12 items) in GPC1 samples. | GPC1 (ten cohorts), UK Biobank | Adults (UK Biobank range 39-73 years, mean age 56.7, SD=8.0; GPC1). Participants of the GPC1 cohorts were primarily adults (one of the ten cohorts, the QIMR study, included participants from 16 to 27 years) | 10.0% | Age, sex, Townsend deprivation index, genotype array, and ten ancestry-based principal components. | sumstats_neuroticism_ctg_format.txt as accessed via the GWAS catalog (GCST006476). |
| Nagel et al., 2018 | Depressed Affect | The genetically identified subcluster 'Depressed Affect' was based on four items of the EPQ-RS (see Nagel et al., 2018): (“Do you often feel lonely?”, “Do you ever feel ‘just miserable’ for no reason?”, “Does your mood often go up and down?”, and “Do you often feel ‘fed up’?”). | UK Biobank | Adults (range 39-73 years, mean age 56.7, SD=8.0) | 8.9% | Age, sex, Townsend deprivation index, genotype array, and ten ancestry-based principal components. | sumstats_depressed_affect_ctg_format.txt.gz as accessed via the GWAS catalog (GCST006475). |
| Nagel et al., 2018 | Worry | The genetically identified subcluster 'Worry' was based on four items of the EPQ-RS (see Nagel et al., 2018): (“Are you a worried?”, “Do you suffer from nerves?”, “Would you call yourself a nervous person?”, and “Would you call yourself tense or highly strung”). | UK Biobank | Adults (range 39-73 years, mean age 56.7, SD=8.0) | 9.0% | Age, sex, Townsend deprivation index, genotype array, and ten ancestry-based principal components. | sumstats_worry_ctg_format.txt.gz as accessed via the GWAS catalog (GCST006478). |
| Wray et al., 2018 | Depression | Cases with a lifetime diagnosis of major depressive disorder were identified according to criteria of the DSM-III, DSM-IV, ICD-9, or ICD-10 based on structured clinical interviews or checklists or by review of the medical records. | PGC (29 cohorts), deCODE, GenScotland, GERA, iPsych. For the MR analyses, the summary statstics without UK Biobank and 23andMe data was used | Adults | SNP-based heritability of the down-sampled GWAS was not reported. | Not specified. | daner_pgc_mdd_meta_w2_no23andMe_rmUKBB.gz as accessed via the website of the PGC (https://pgc.unc.edu/for-researchers/download-results/). |
| Harder et al., 2022 | Age at Onset of Depression | Cases with major depression were identified via 1.) a history of major depression in the baseline interview (N=26,425) or based on 2.) lifetime symptoms of major depression (N=76,365) as questioned in the Composite International Diagnostic Criteria Short Form (CIDI-SF) as part of the Mental Health Questionnaire (MHQ). For the first cases, the age at diagnosis was obtained (reported mean age at diagnosis 42.7, SD=13.1), for the second cases, the age at onset of symptoms was asked (reported mean age at onset of symptoms 37.3, SD=15). Both measures were pooled in a single GWAS to obtain the phenotype 'Age at Onset of Depression'. | UK Biobank | Adults (mean age for participants of 'age at diagnosis' at time of data collection 55.4, SD=7.80; mean age for participants of 'age at symptoms' at time of data collection 55.4, SD=7.67) | 5.6% | Not specified. | GCST90101808.tsv as accessed via the GWAS catalog (GCST90101808). |
| Otowa et al., 2016 | Anxiety Disorders | An anxiety factor score was defined combining information (diagnosis, severity) across the spectrum of anxiety disorders. Diagnosis of disorders (general anxiety disorder, agoraphobia, panic attacks, panic disorder, social phobia, specific phobia, major depressive disorder) were based on DSM-IV criteria derived from information obtained via (clinical) questionnaires and interviews (Schedule for Affective Disorders and Schizophrenia - Lifetime and Anxiety disorder version (SADS-LA); lifetime version of the Composite International Diagnostic Interview, Short Form (CIDI-SF) or Munich version (M-CIDI); (Semi-Structured Assessment for the Genetics of Alcoholism (SSAGA); | PGC (8 cohorts) | Adults | 10.6% | Sex, age at interview, and ancestry principal components depending on the respective sample. | anxiety.meta.full.fs.tbl.gz as accessed via the website of the PGC (https://pgc.unc.edu/for-researchers/download-results/). |
