# Supplementary material for: The causal role of male pubertal timing for the development of externalizing and internalizing traits: results from Mendelian randomization studies
Source: Psychol Med. 2025 Mar 28;55:e101. doi: 10.1017/S0033291725000352 (PMC12094630; doi:10.1017/S0033291725000352)
Supplement: Dinkelbach et al. supplementary material 2 — Dinkelbach et al. supplementary material [file S0033291725000352sup002.docx]

# Supplementary Material

**The causal role of male pubertal timing for the development of externalizing and internalizing traits: results from Mendelian randomization studies**

Lars Dinkelbach, Triinu Peters, Corinna Grasemann, Anke Hinney, and Raphael Hirtz

# Overview

# Supplementary Methods 1 – Calculation of effect estimates

# Supplementary Methods 2 – Software

# Supplementary Results 1 – Details on outcomes with primary endpoint p value < 0.05

Externalizing Traits

Supplementary Figure S1 – Scatter Plot - Externalizing Traits

Supplementary Figure S2 – Funnel Plot - Externalizing Traits

# Supplementary Figure S3 – Forest Plot - Externalizing Traits

# Supplementary Figure S4 – Leave-one-out analysis - Externalizing Traits

Age at First Sexual Contact

Supplementary Figure S5 – Scatter Plot – Age at First Sexual Contact

# Supplementary Figure S6 – Funnel Plot – Age at First Sexual Contact

# Supplementary Figure S7 – Forest Plot – Age at First Sexual Contact

# Supplementary Figure S8 – Leave-one-out analysis – Age at First Sexual Contact

Early Life Internalizing Traits

Supplementary Figure S9 – Scatter Plot – Early Life Internalizing Traits

# Supplementary Figure S10 – Funnel Plot – Early Life Internalizing Traits

# Supplementary Figure S11 – Forest Plot – Early Life Internalizing Traits

# Supplementary Figure S12 – Leave-one-out analysis – Early Life Internalizing Traits

Depressed Affect

Supplementary Figure S13 – Scatter Plot – Depressed Affect

# Supplementary Figure S14 – Funnel Plot – Depressed Affect

# Supplementary Figure S15 – Forest Plot – Depressed Affect

# Supplementary Figure S16 – Leave-one-out analysis – Depressed Affect

Age at Onset of Depression

Supplementary Figure S17 – Scatter Plot – Age at Onset of Depression

# Supplementary Figure S18 – Funnel Plot – Age at Onset of Depression

# Supplementary Figure S19 – Forest Plot – Age at Onset of Depression

# Supplementary Figure S20 – Leave-one-out analysis – Age at Onset of Depression

Supplementary Figure S21 – Sensitivity Analyses – Age at Onset of Depression

# Supplementary Results 2 – Results of outcomes with primary endpoint p value > 0.05

Supplementary Figure S22 – Sensitivity Analyses – Alcohol Dependency

Supplementary Figure S23 – Sensitivity Analyses – Risk Tolerance

Supplementary Figure S24 – Sensitivity Analyses – Extraversion

Supplementary Figure S25 – Sensitivity Analyses – Cannabis Abuse

Supplementary Figure S26 – Sensitivity Analyses – Number of Sexual Partners

Supplementary Figure S27 – Sensitivity Analyses – Ever Smoker

Supplementary Figure S28 – Sensitivity Analyses – Early Life Aggression

Supplementary Figure S29 – Sensitivity Analyses – Antisocial Behavior

Supplementary Figure S30 – Sensitivity Analyses – Worry

Supplementary Figure S31 – Sensitivity Analyses – Neuroticism

Supplementary Figure S32 – Sensitivity Analyses – Anxiety Disorders

Supplementary Figure S33 – Sensitivity Analyses – Depression

Supplementary References

# Supplementary Methods

**Methods 1 – Calculation of effect estimates**

First, the MR-PRESSO method was calculated ([Verbanck, Chen, Neale, & Do, 2018](#_ENREF_21)). This method is based on three parts a.) the MR-PRESSO global test, which detects whether overall horizontal pleiotropy is present by comparing the observed effect of each SNP with the estimated effect (obtained via calculation of the effect estimate without the respective SNP). The observed residual sum of squares (RSSobs) is then compared with a simulated expected distribution of the residual sum of squares (using a Gaussian distribution), and this comparison is used to measure total horizontal pleiotropy. b.) The MR-PRESSO outlier test, where the same approach is used to detect horizontal pleiotropy of single variants, and c.) the MR-PRESSO distortion test, which evaluates whether the effect estimates differ with and without the exclusion of pleiotropic outliers ([Verbanck et al., 2018](#_ENREF_21)). As the primary endpoint for all outcomes, the inverse-variance weight (IVW) effect estimate was calculated after excluding pleiotropic outliers ([Burgess, Butterworth, & Thompson, 2013](#_ENREF_4)). The IVW effect estimate combines the effect estimate for the exposure on the outcome for each SNP included in the IV, similar to a fixed-effect meta-analysis ([Burgess et al., 2013](#_ENREF_4)). As sensitivity analyses, the following set of so-called robust methods was calculated: Weighted median- and mode-based estimates ([Bowden, Davey Smith, Haycock, & Burgess, 2016](#_ENREF_2); [Hartwig, Davey Smith, & Bowden, 2017](#_ENREF_6)), MR Egger ([Bowden, Davey Smith, & Burgess, 2015](#_ENREF_1)), and MR-RAPS ([Zhao, Wang, Hemani, Bowden, & Small, 2020](#_ENREF_26)). Weighted median- and mode-based analyses are less powered but give valid estimates even in situations when just the majority (or plurality, for mode-based estimation) of instruments with similar causal effect estimates fulfill the MR assumption ([Bowden, Davey Smith, et al., 2016](#_ENREF_2); [Hartwig et al., 2017](#_ENREF_6)). MR Egger corrects for directional pleiotropy by the introduction of an intercept in the resulting regression model and thus allows all genetic variants to be invalid variants ([Bowden et al., 2015](#_ENREF_1)). However, MR Egger estimates are again less powered and sensitive towards violations of the ‘Instrument Strength Independent of Direct Effect’ (InSIDE) assumption, which postulates that the causal effect of a variant on the outcome and the direct (pleiotropic) effect of a genetic variant are uncorrelated. In addition, MR Egger relies on the ‘no measurement error’ (NOME) assumption, assuming that the association between each genetic variant and the exposure is measured without error ([Bowden, Del Greco M, et al., 2016](#_ENREF_3)). A violation of these assumptions and its effect on the validity of the MR Egger test can be estimated by the *I^2^* statistic ([Bowden, Del Greco M, et al., 2016](#_ENREF_3)). To assess violations of the NOME assumption, the *I*^2^ statistics were calculated for each MR Egger analysis. An *I*² < 0.9 indicate a relative bias in the MR Egger estimate greater than 10%, thus this value was used as a cut-off for a relevant violation of the NOME assumption ([Bowden, Del Greco M, et al., 2016](#_ENREF_3)). Lastly, an MR-RAPS estimate was calculated, considering systematic pleiotropy by modeling the pleiotropic effect of each genetic variant with a random-effects distribution ([Zhao et al., 2020](#_ENREF_26)).

*Multivariable MR analysis with BMI*

An MVMR analysis was conducted for each outcome with BMI as an additional exposure to account for pleiotropy due to differences in body weight. Therefore, the GWAS on BMI in males was used from 374,756 participants from 29 cohors of the Genetic Investigation of ANthropometric Traits (GIANT) consortium and from the UK Biobank ([Pulit et al., 2019](#_ENREF_18)).

*Data modification prior to MR analyses*

For the outcome ‘Early Life Internalizing Traits’, beta values were calculated from z-scores and se (beta = z * se). For the outcome ‘Depression’, beta values were calculated from odds ratios (beta = ln(OR)). For the outcomes ‘Neuroticism’, ‘Cannabis Abuse’, ‘Alcohol Dependency’ and ‘Antisocial Behavior’ only z-scores were available in the reported summary statistics. Beta values and standard errors were calculated from z scores, sample size and minor allele frequency (MAF) with the formula provided by [Zhu et al. (2016)](#_ENREF_27). For ‘Cannabis Abuse’, ‘Alcohol Dependency’ and ‘Antisocial Behavior’ no MAFs were reported, therefore these were approximated by the MAFs as reported in the GWAS on depression from [Wray et al. (2018)](#_ENREF_24). The GWAS by [Wray et al. (2018)](#_ENREF_24) as well as the GWAS for ‘Cannabis Abuse’, ‘Alcohol Dependency’ and ‘Antisocial Behavior’ were based on similar consortia data with a presumably large sample overlap. Thus, it could be assumed that the MAFs as reported by [Wray et al. (2018)](#_ENREF_24) provided a good approximation for the MAFs of the GWAS for ‘Cannabis Abuse’, ‘Alcohol Dependency’ and ‘Antisocial Behavior’. F-statistics were calculated by F=beta²/se².

*Harmonization*

The effects of genetic variants were harmonized between the exposure and outcome GWAS to ensure that the effect of each variant on both the exposure and outcome corresponded to the same allele. For palindromic SNPs, it was assumed that all alleles were reported on the forward strand, as effect allele frequencies were not available in all outcome GWAS to reliably infer the forward strand orientation.

**Methods 2 - Software**

The exclusion of pleiotropic outliers and the MR-PRESSO global test were conducted with MR-PRESSO (version 1.0, ([Verbanck et al., 2018](#_ENREF_21))). The harmonization of genetic variants and the calculation of the IVW, MR Egger, the weighted median, and weighted mode estimators were conducted using the TwoSampleMR package (version 0.5.9, ([Hemani et al., 2018](#_ENREF_8))). The MR-RAPS estimate (considering overdispersion and a I^2^-loss-function) was calculated with the mr.raps package (version 0.2, ([Zhao et al., 2020](#_ENREF_26))). For the calculation of *I*^2^, the MendelianRandomization package was used (version 0.9.0, ([Yavorska & Burgess, 2017](#_ENREF_25))). The MVMR analyses were conducted using the MVMR extensions of the MR-PRESSO and the MendelianRandomization package ([Verbanck et al., 2018](#_ENREF_21); [Yavorska & Burgess, 2017](#_ENREF_25)). The CheckSumStats package was used to confirm the correct annotation of the effect allele and the European population of the summary statistics utilized for the current MR analysis (version 0.0.0.9, only possible if the effect allele frequency was specified in the respective GWAS, ([Haycock et al., 2023](#_ENREF_7))). The graphics were built with forestploter (version 1.1.1). All analyses were conducted using R version 4.3.2.

**Supplementary Results**

**Supplementary Results 1 – Details on outcomes with primary endpoint p value < 0.05**

**Outcome: Externalizing Traits**

65 out of the 76 target SNPs of male puberty timing were available in the outcome GWAS by [Williams et al. (2023)](#_ENREF_23) and [Karlsson Linnér et al. (2021)](#_ENREF_14). MR-PRESSO identified seven pleiotropic SNPs. Thus, the instrumental variable consisted of 58 genetic variants. The exclusion of outliers by MR-PRESSO did not alter the results of the primary endpoint (see ‘MR-PRESSO raw’ IVW estimate in Figure 3A). The scatter plots, funnel plots, forest plots, and leave-one-out analyses did not provide any evidence of violations of the Mendelian Randomization (MR) assumptions, nor were the results influenced by individual variants.


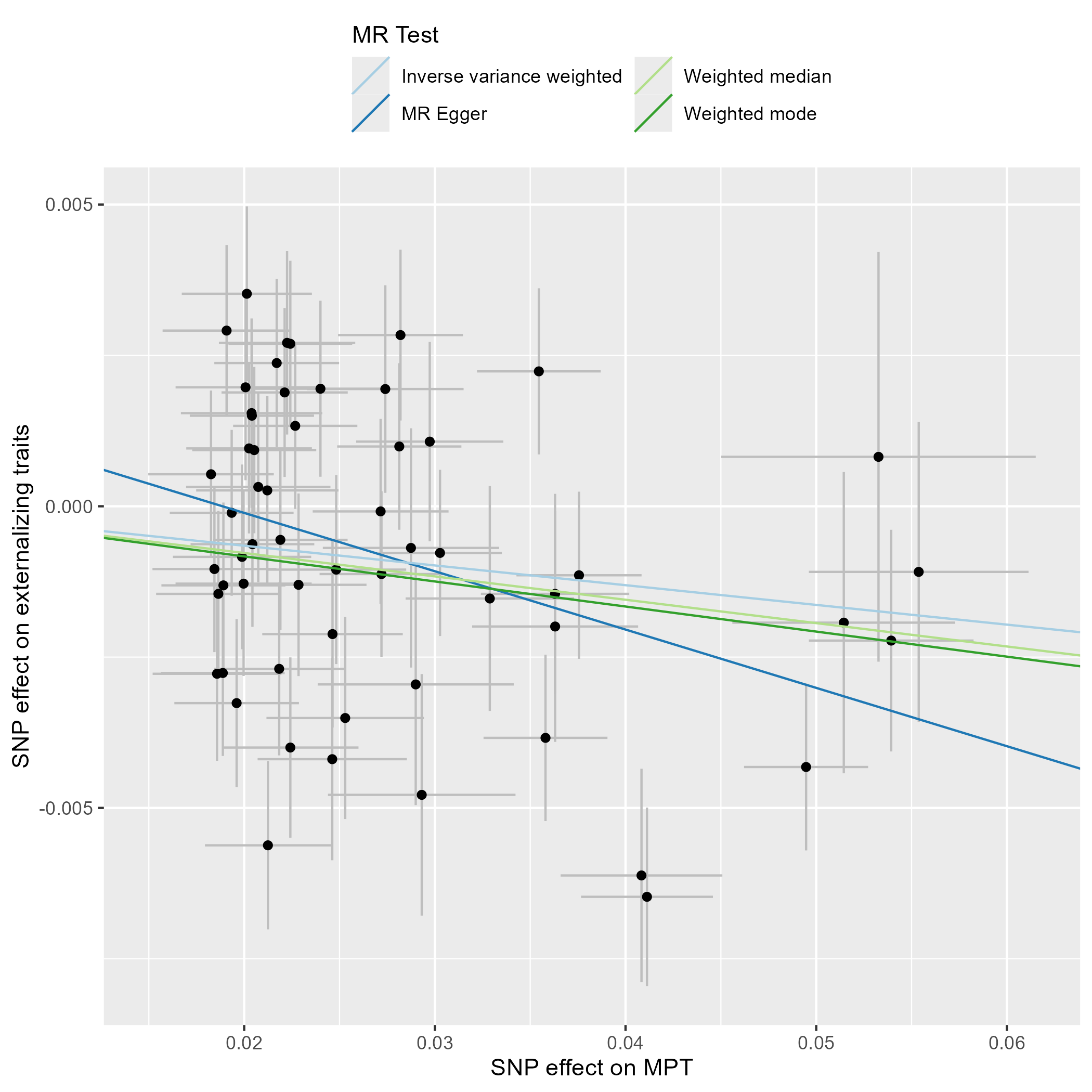


**Supplementary Figure S1 – Scatter Plot - Externalizing Traits.** This scatter plot depicts the effect estimates (beta, error bars represent the standard error) of each genetic variant included in the analysis on the exposure (male puberty timing ([Hollis et al., 2020](#_ENREF_9))) and the outcome (externalizing traits, ([Karlsson Linnér et al., 2021](#_ENREF_14))).


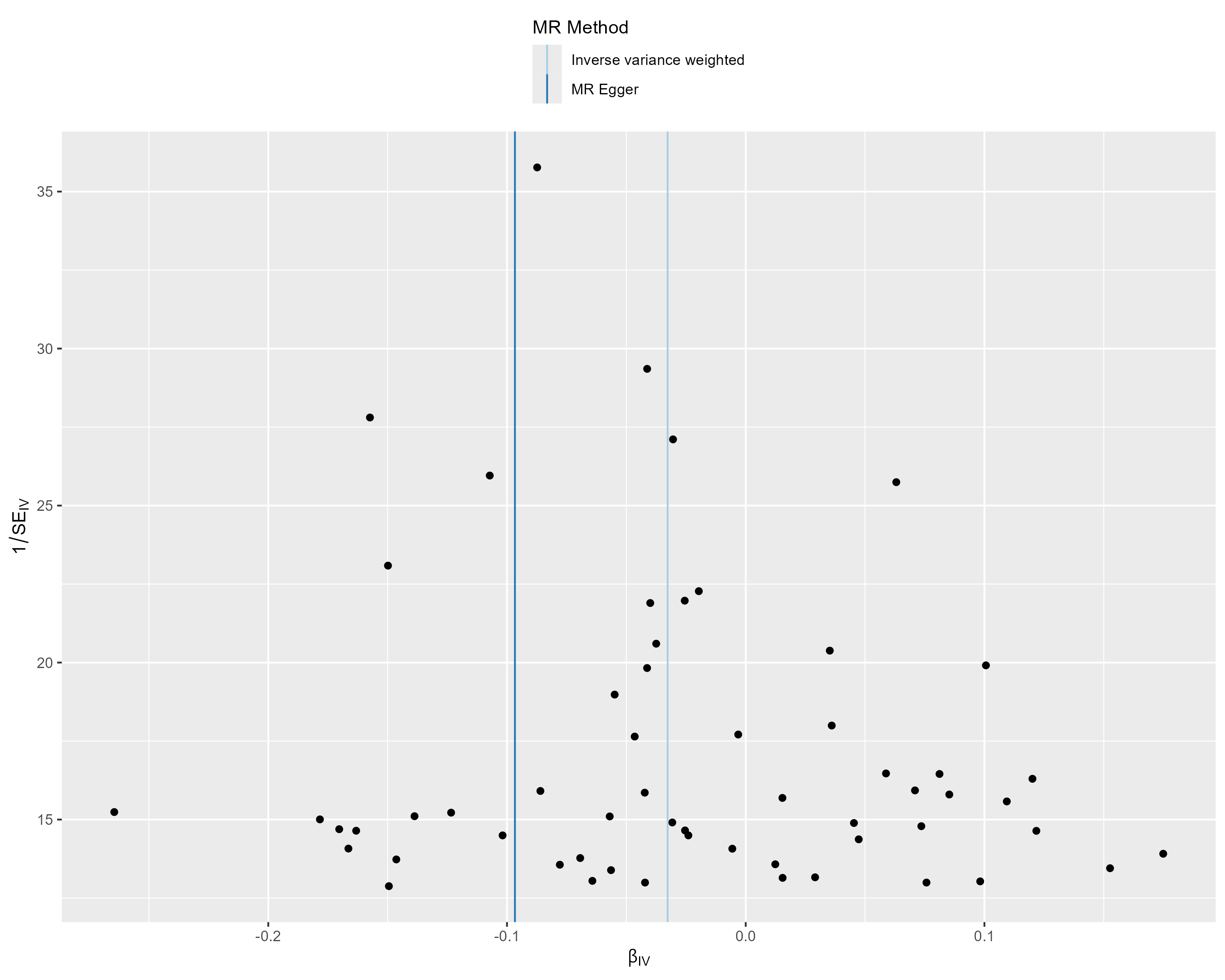


**Supplementary Figure S2 – Funnel Plot - Externalizing Traits.** This funnel plot illustrates the precision of each genetic variant (as measured by the inverse of the standard error (SE_IV_)) and their MR effect estimate (β_IV_).


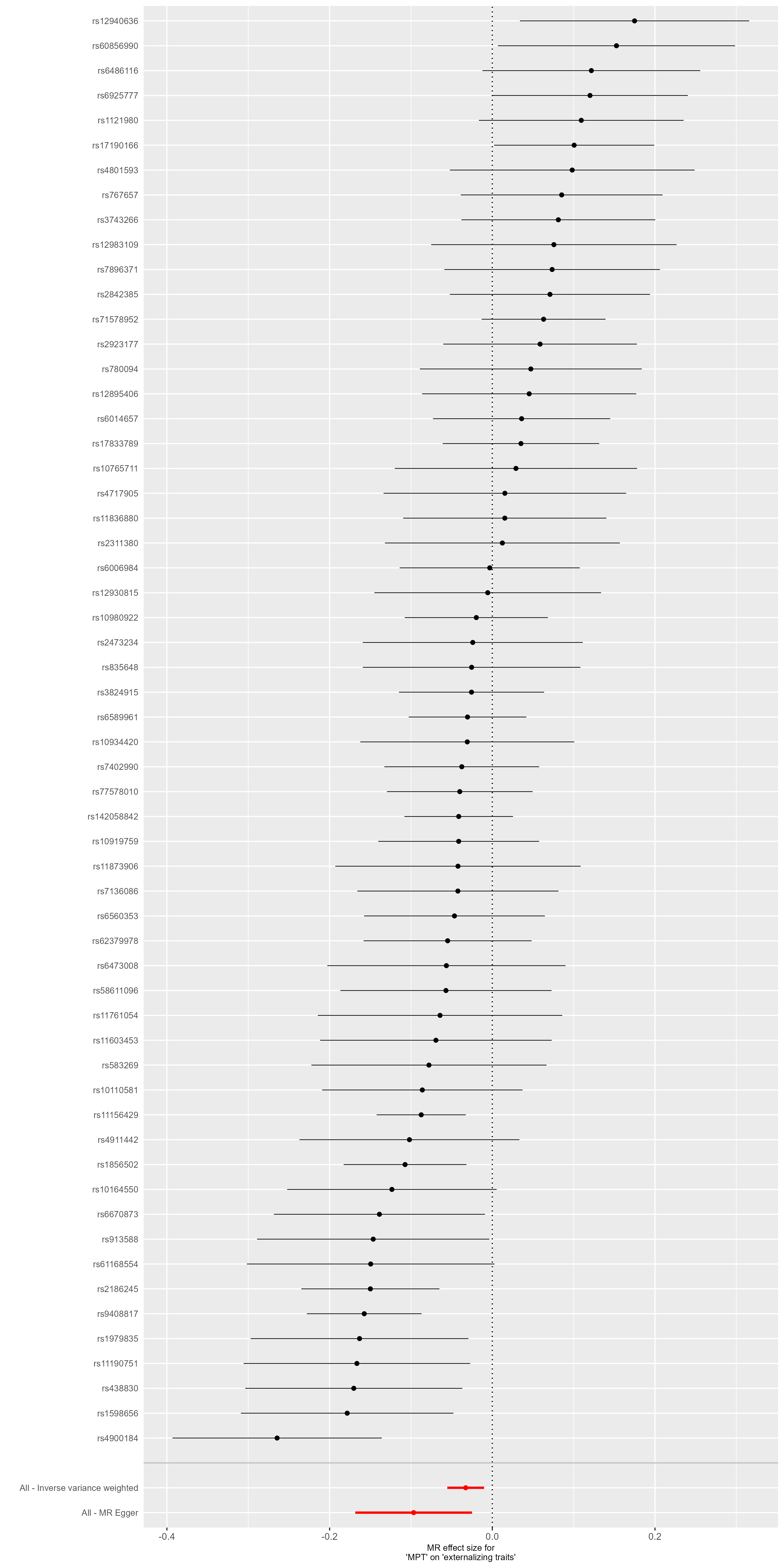


**Supplementary Figure S3 – Forest Plot - Externalizing Traits.** This forest plot illustrates the results of single-SNP MR analyses with the MR effect estimates for each SNP on the outcome (Externalizing Traits).


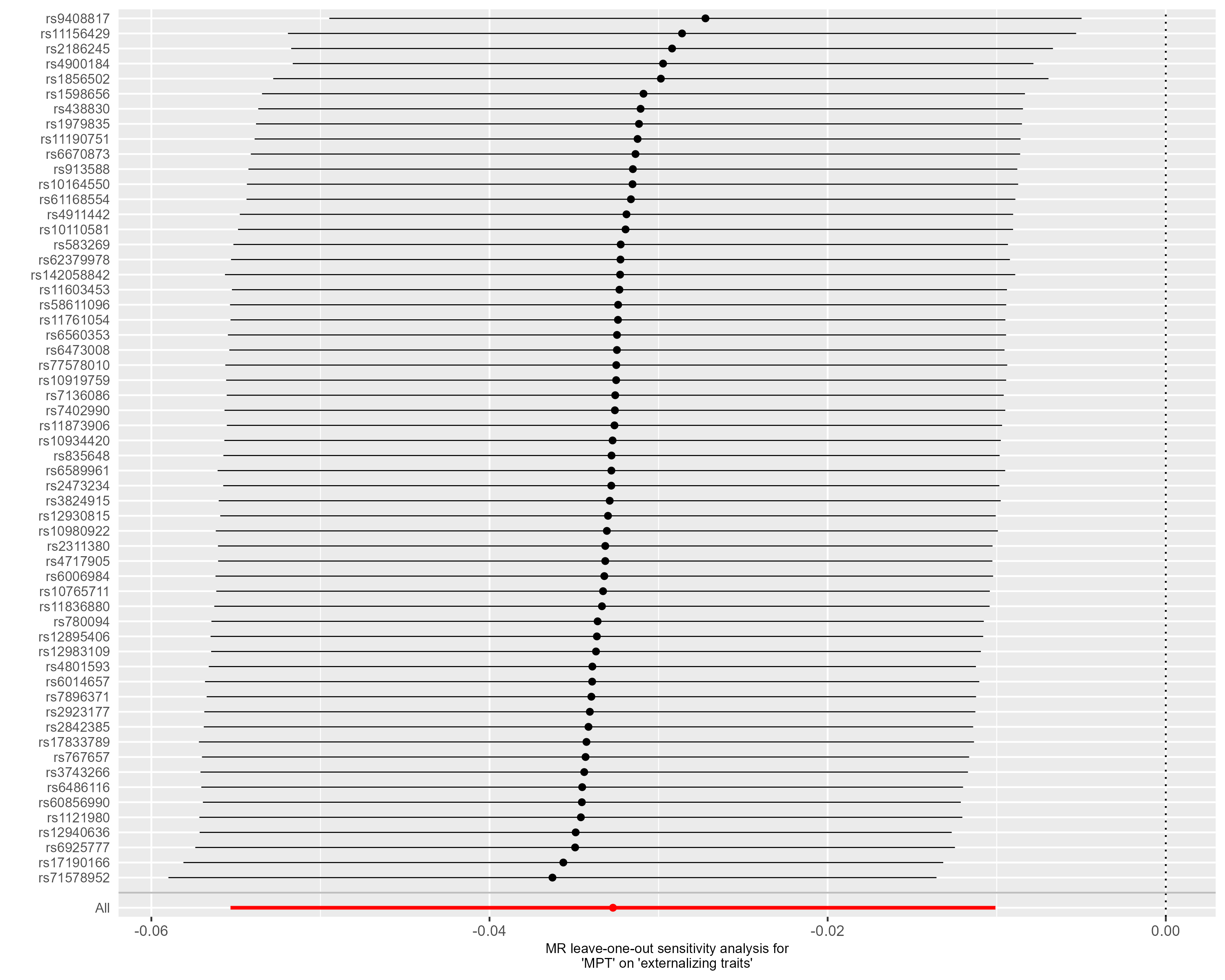


**Supplementary Figure S4 – Leave-one-out analysis - Externalizing Traits.** This forest plot illustrates the results of MR analyses (IVW) following the exclusion of individual genetic variants from the instrumental variable through leave-one-out analysis.

**Outcome: Age at First Sexual Contact**

All 76 target SNPs of male puberty timing were available in the outcome GWAS by [Mills et al. (2021)](#_ENREF_15). MR-PRESSO identified five pleiotropic SNPs. Thus, the instrumental variable consisted of 71 genetic variants. The exclusion of outliers by MR-PRESSO did not alter the results of the primary endpoint (see ‘MR-PRESSO raw’ IVW estimate in Figure 3B). The scatter plots, funnel plots, forest plots, and leave-one-out analyses did not provide any evidence of violations of the Mendelian Randomization (MR) assumptions, nor were the results influenced by individual variants.


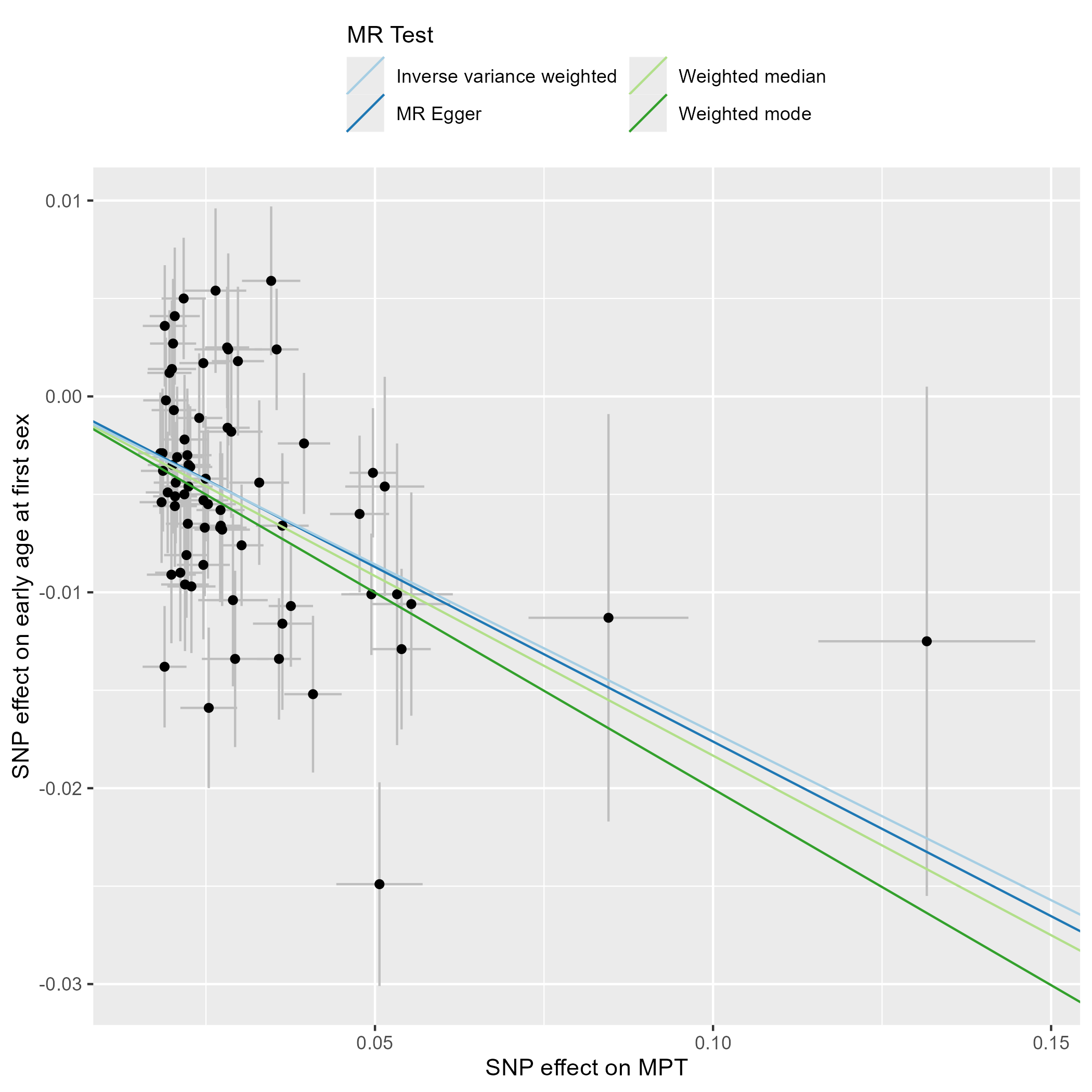


**Supplementary Figure S5 – Scatter Plot – Age at First Sexual Contact.** This scatter plot depicts the effect estimates (beta, error bars represent the standard error) of each genetic variant included in the analysis on the exposure (male puberty timing ([Hollis et al., 2020](#_ENREF_9))) and the outcome (age at first sex, ([Mills et al., 2021](#_ENREF_15))).


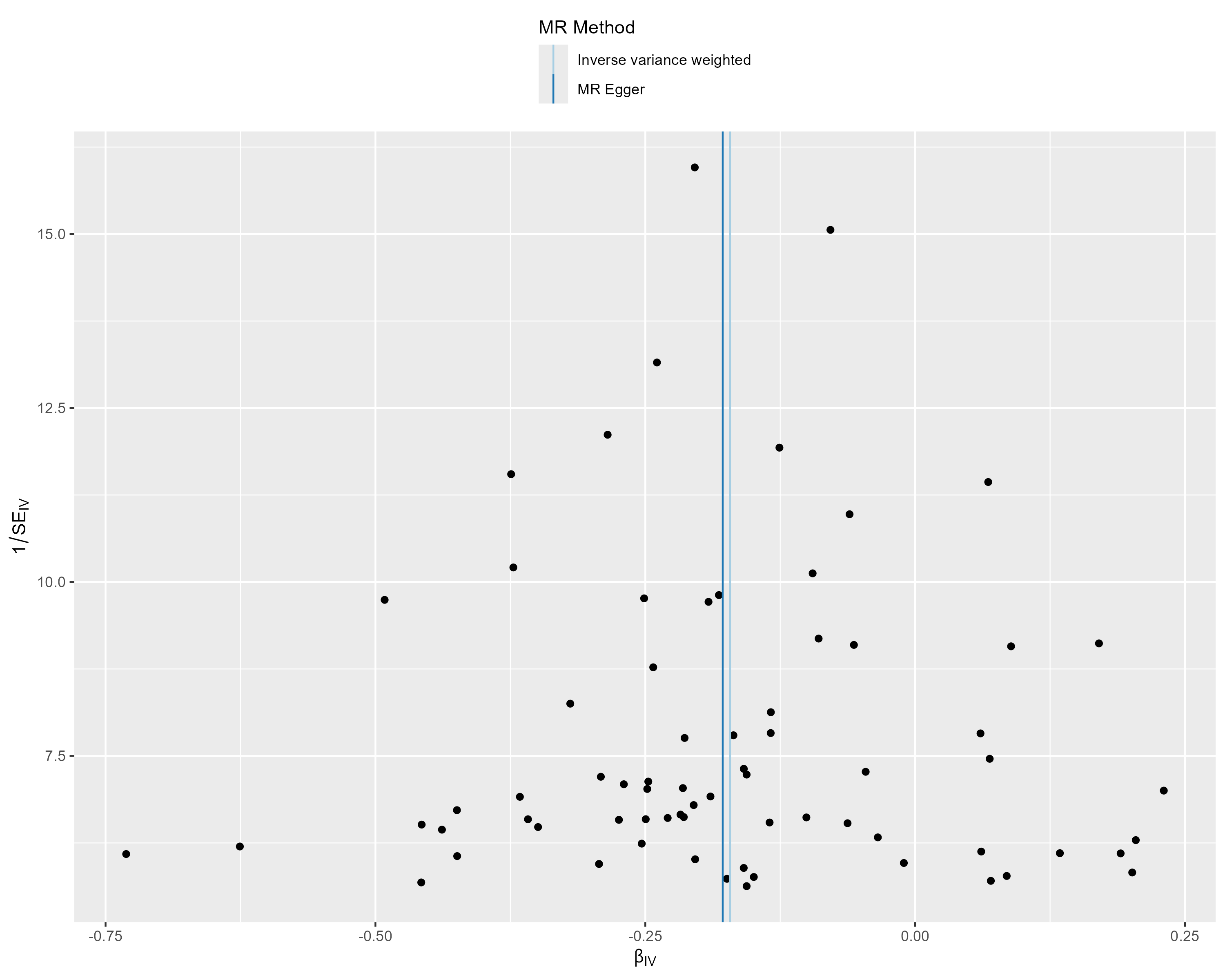


**Supplementary Figure S6 – Funnel Plot - Age at First Sexual Contact.** This funnel plot illustrates the precision of each genetic variant (as measured by the inverse of the standard error (SE_IV_)) and their MR effect estimate (β_IV_).


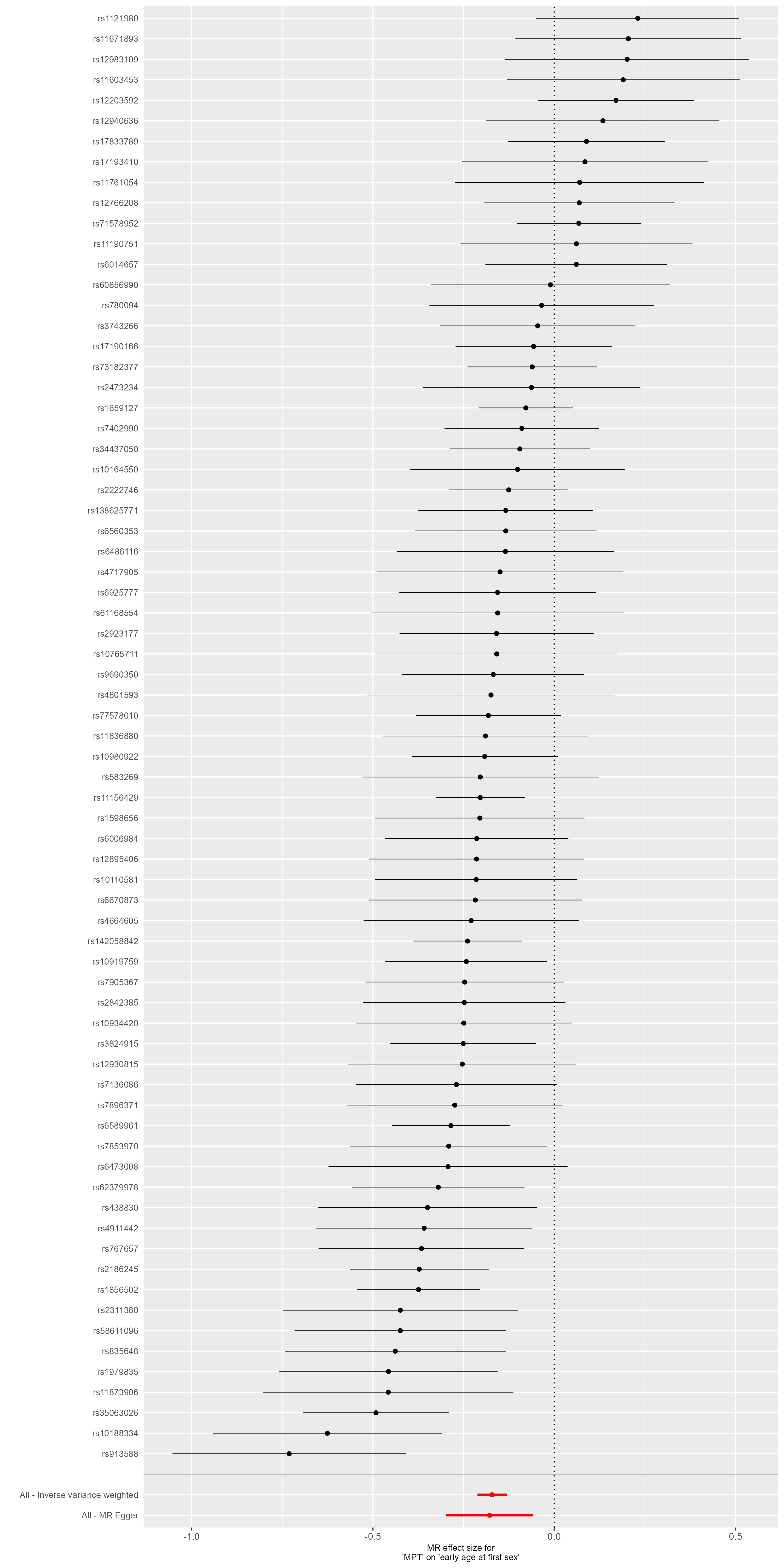


**Supplementary Figure S7 – Forest Plot - Age at First Sexual Contact.** This forest plot illustrates the results of single-SNP MR analyses with the MR effect estimates for each SNP on the outcome (Age at first sexual Intercourse, inversely coded). Negative effect estimates indicate that early male puberty is related to younger age at first sexual contact.


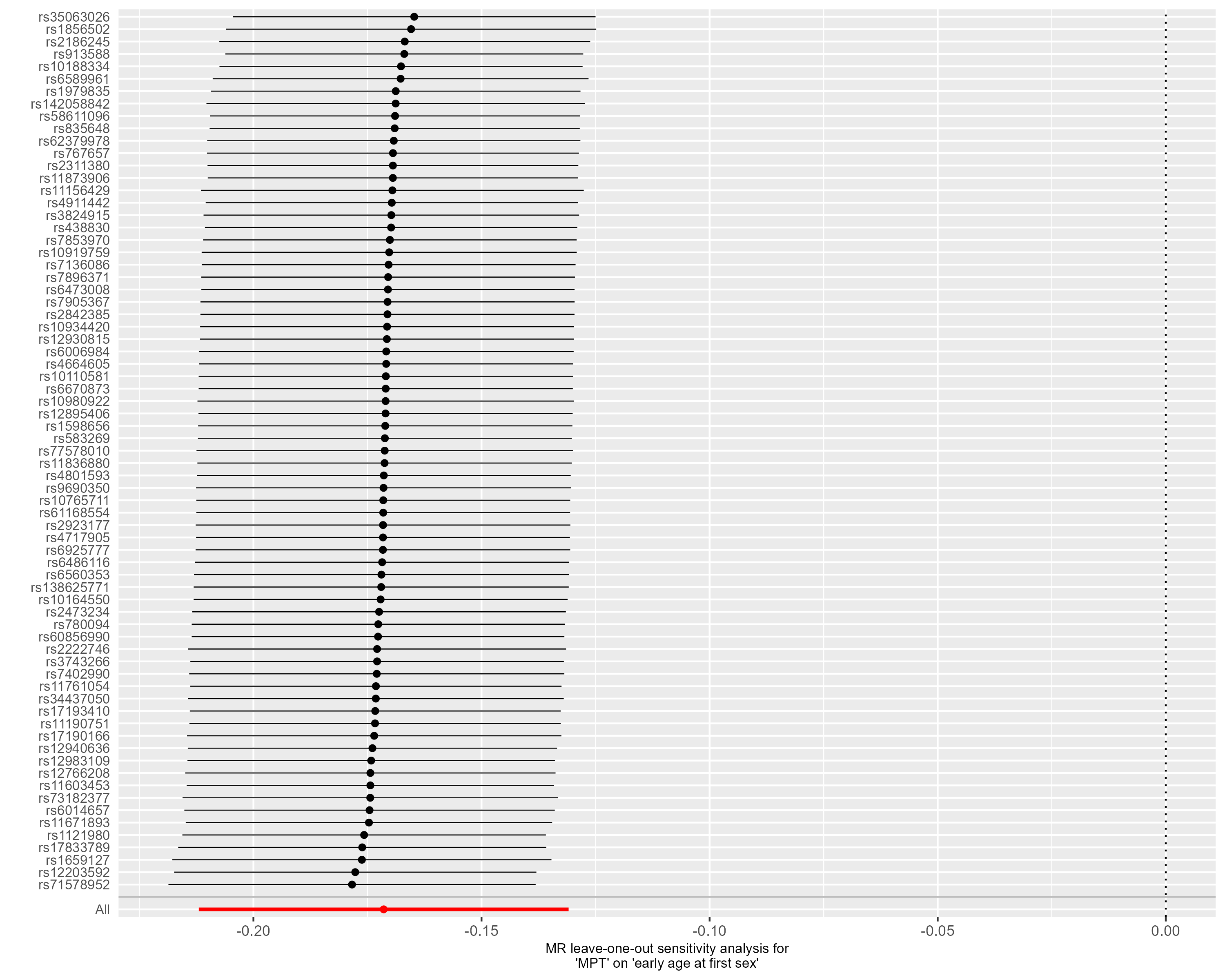


**Supplementary Figure S8 – Leave-one-out analysis - Age at First Sexual Contact.** This forest plot illustrates the results of MR analyses (IVW) following the exclusion of individual genetic variants from the instrumental variable through leave-one-out analysis.

**Outcome: Early Life Internalizing Traits**

71 of the 76 target SNPs of male puberty timing were available in the outcome GWAS by [Jami et al. (2022)](#_ENREF_11). No outliers were identified by MR-PRESSO. Thus, the instrumental variable consisted of 71 genetic variants. The scatter plots, funnel plots, forest plots, and leave-one-out analyses did not provide any evidence of violations of the Mendelian Randomization (MR) assumptions, nor were the results influenced by individual variants.


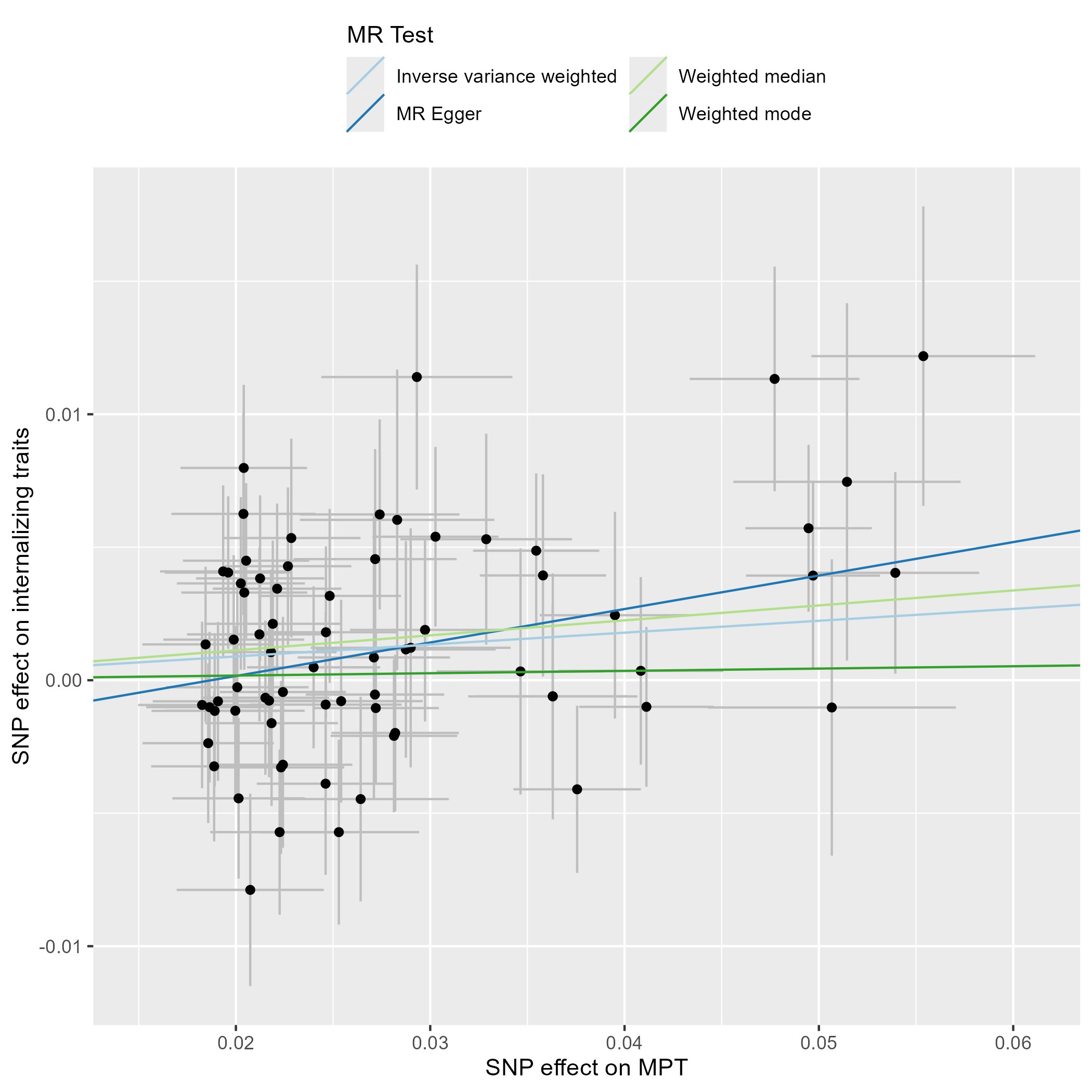


**Supplementary Figure S9 – Scatter Plot – Early Life Internalizing Traits.** This scatter plot depicts the effect estimates (beta, error bars represent the standard error) of each genetic variant included in the analysis on the exposure (male puberty timing ([Hollis et al., 2020](#_ENREF_9))) and the outcome (internalizing traits in children and adolescents, ([Jami et al., 2022](#_ENREF_11))).


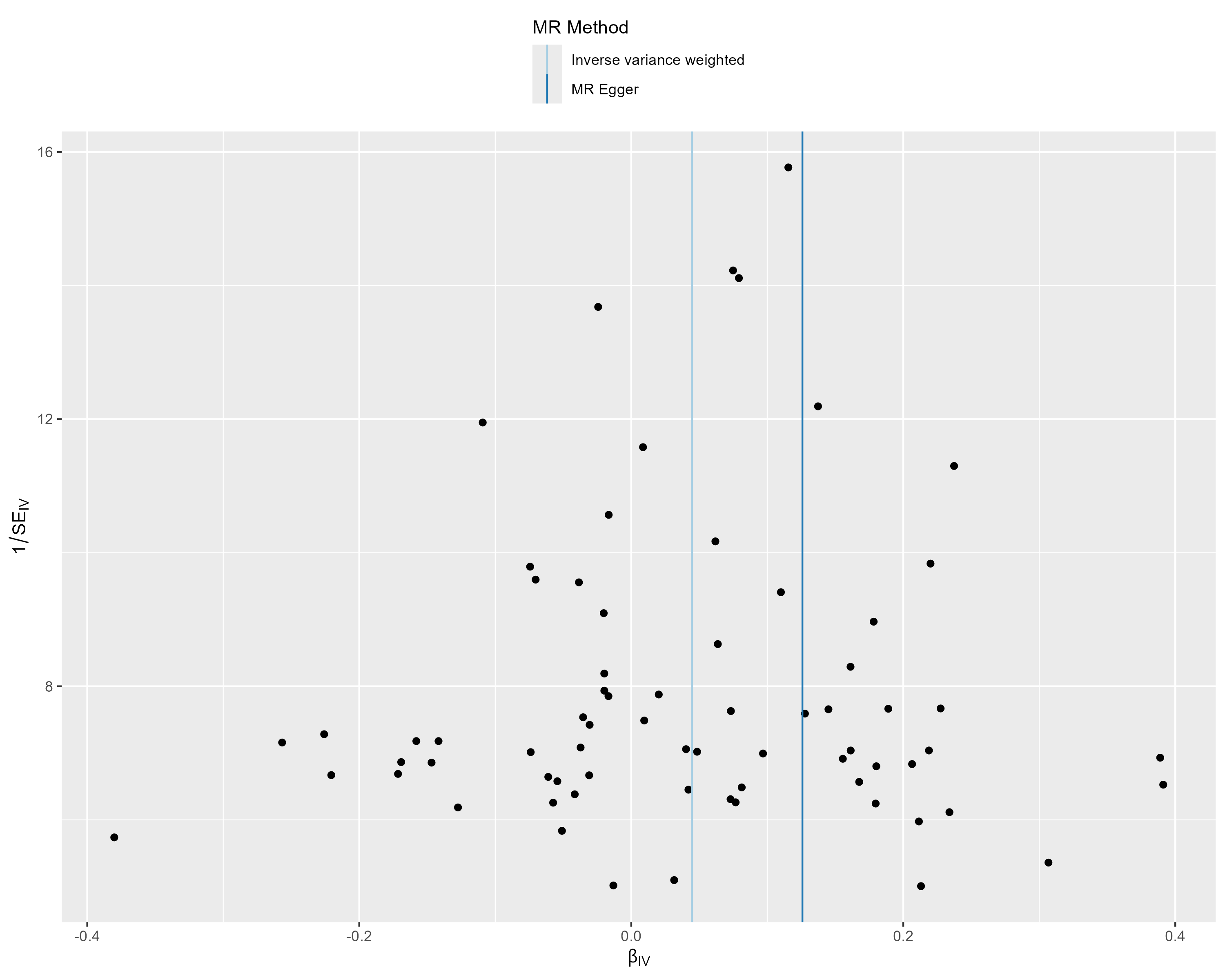


**Supplementary Figure S10 – Funnel Plot - Early Life Internalizing Traits.** This funnel plot illustrates the precision of each genetic variant (as measured by the inverse of the standard error (SE_IV_)) and their MR effect estimate (β_IV_).


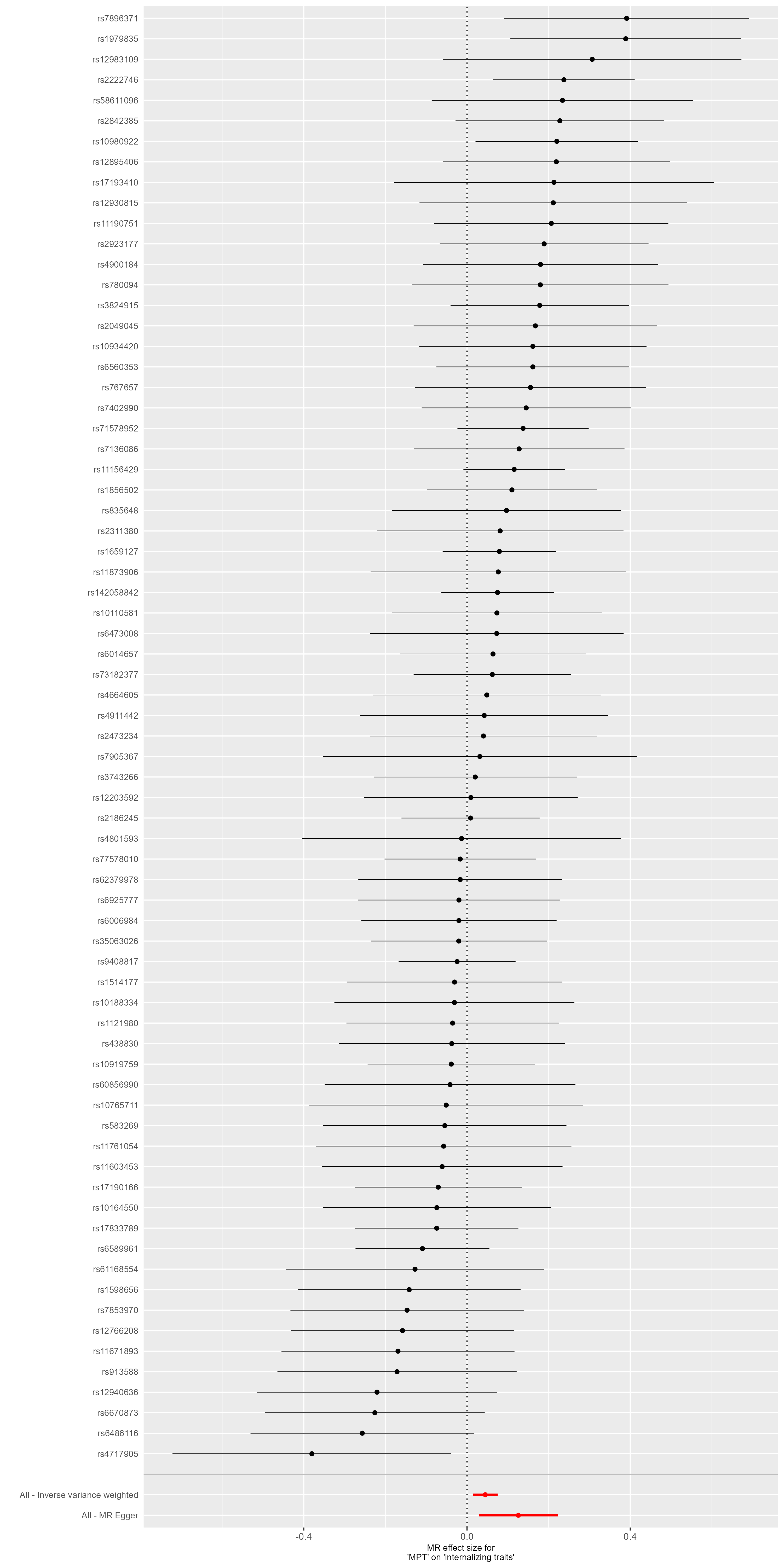


**Supplementary Figure S11 – Forest Plot - Early Life Internalizing Traits.** This forest plot illustrates the results of single-SNP MR analyses with the MR effect estimates for each SNP on the outcome (Internalizing Traits in children and adolescents).


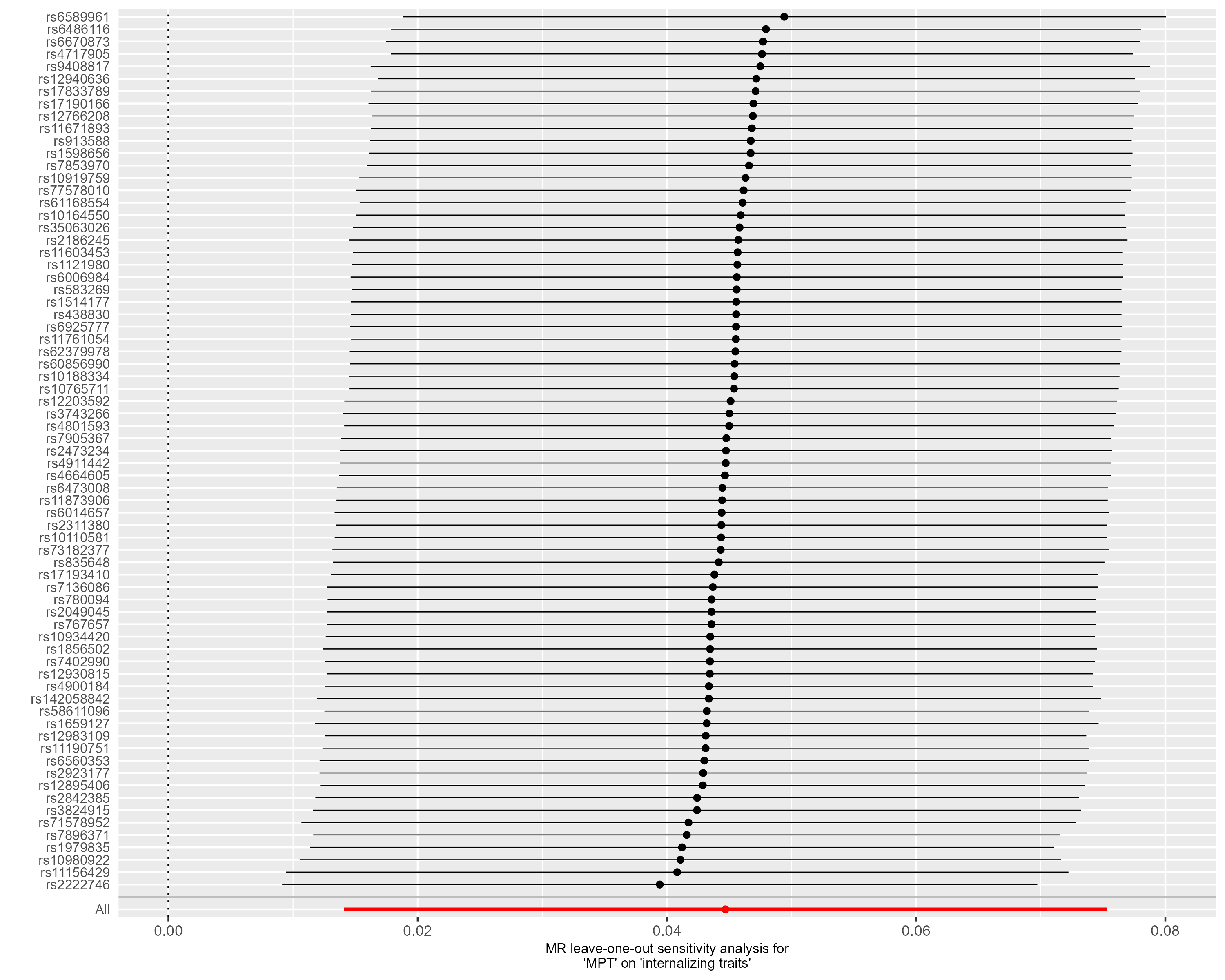


**Supplementary Figure S12 – Leave-one-out analysis - Early Life Internalizing Traits.** This forest plot illustrates the results of MR analyses (IVW) following the exclusion of individual genetic variants from the instrumental variable through leave-one-out analysis.

**Outcome: Depressed Affect**

72 of the 76 target SNPs of male puberty timing were available in the outcome GWAS by [Nagel et al. (2018)](#_ENREF_16). One outlier identified by MR-PRESSO. Thus, the instrumental variable consisted of 71 genetic variants. The exclusion of the outlier by MR-PRESSO did not alter the results of the primary endpoint (see ‘MR-PRESSO raw’ IVW estimate in Figure 3D). The funnel plot revealed a particularly precise genetic variant (rs11156429) whose effect estimate differed from those obtained by the IVW and MR Egger methods, suggesting a bias due to pleiotropy (see Supplementary Figure S14). Nonetheless, even when this variant was excluded in a leave-one-out analysis, the effect of male puberty timing on the outcome remained significant (refer to Supplementary Figure S16).


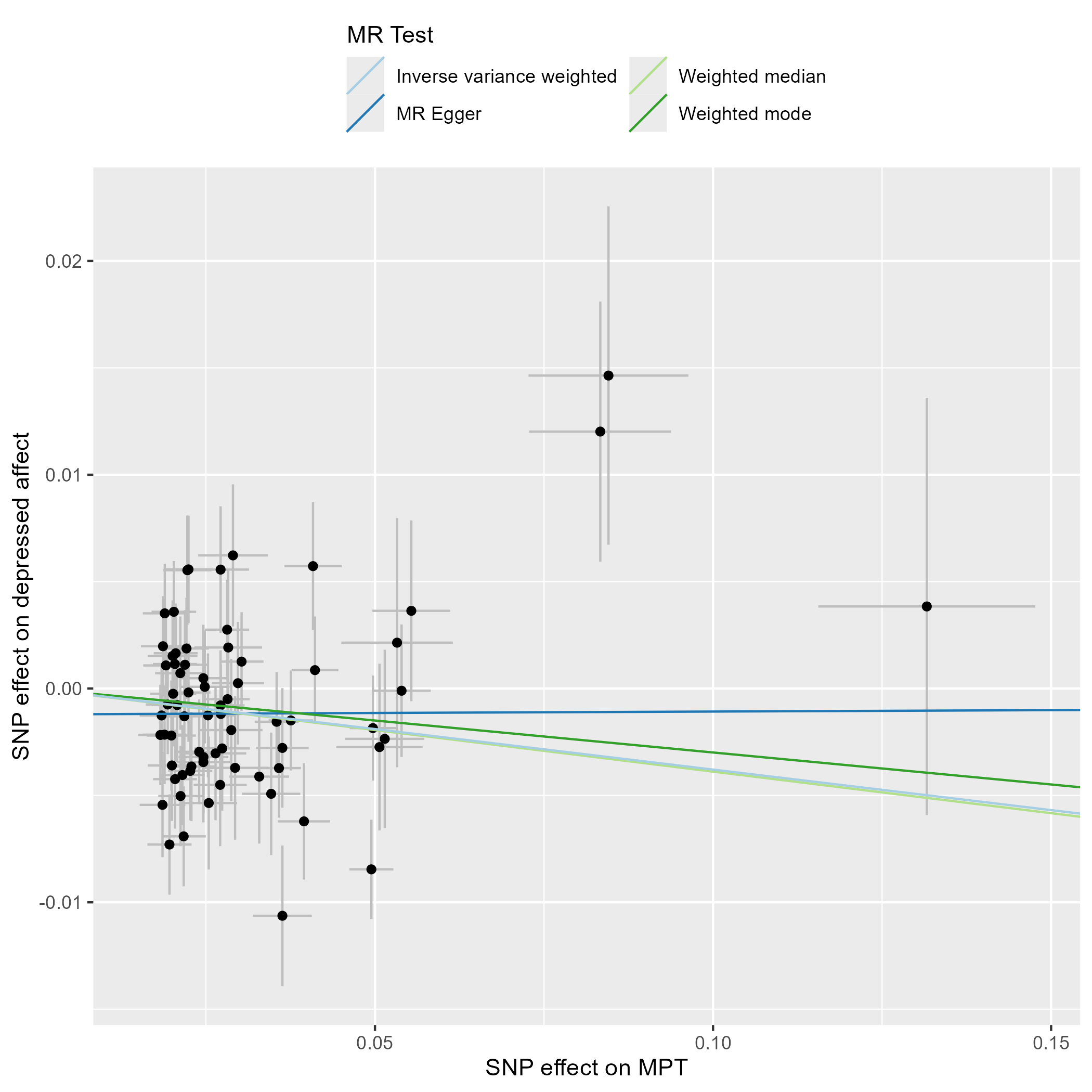


**Supplementary Figure S13 – Scatter Plot – Depressed Affect.** This scatter plot depicts the effect estimates (beta, error bars represent the standard error) of each genetic variant included in the analysis on the exposure (male puberty timing ([Hollis et al., 2020](#_ENREF_9))) and the outcome (‘Depressed Affect’ subdomain of Neuroticism, ([Nagel et al., 2018](#_ENREF_16))).


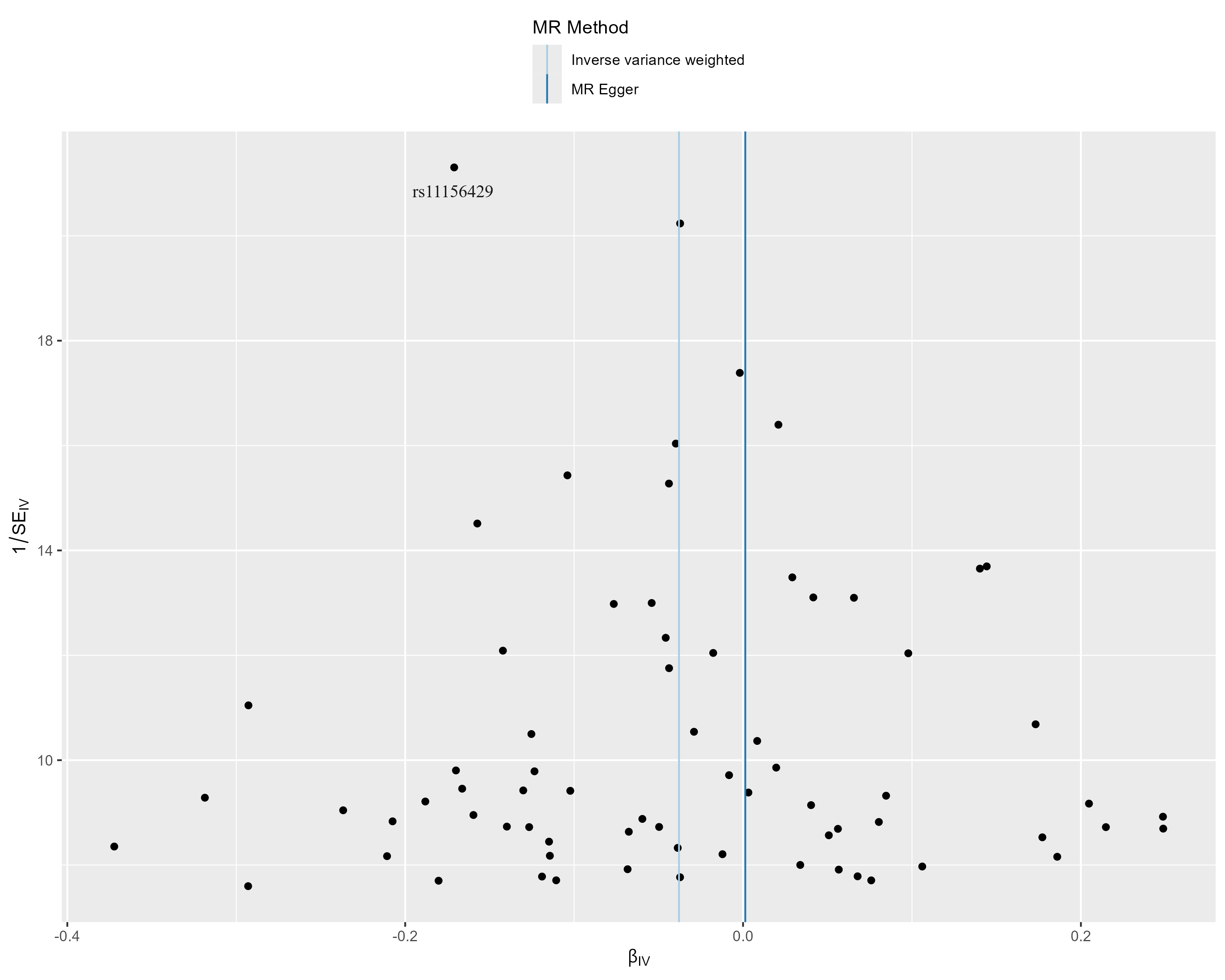


**Supplementary Figure S14 – Funnel Plot - Depressed Affect.** This funnel plot illustrates the precision of each genetic variant (as measured by the inverse of the standard error (SE_IV_)) and their MR effect estimate (β_IV_).


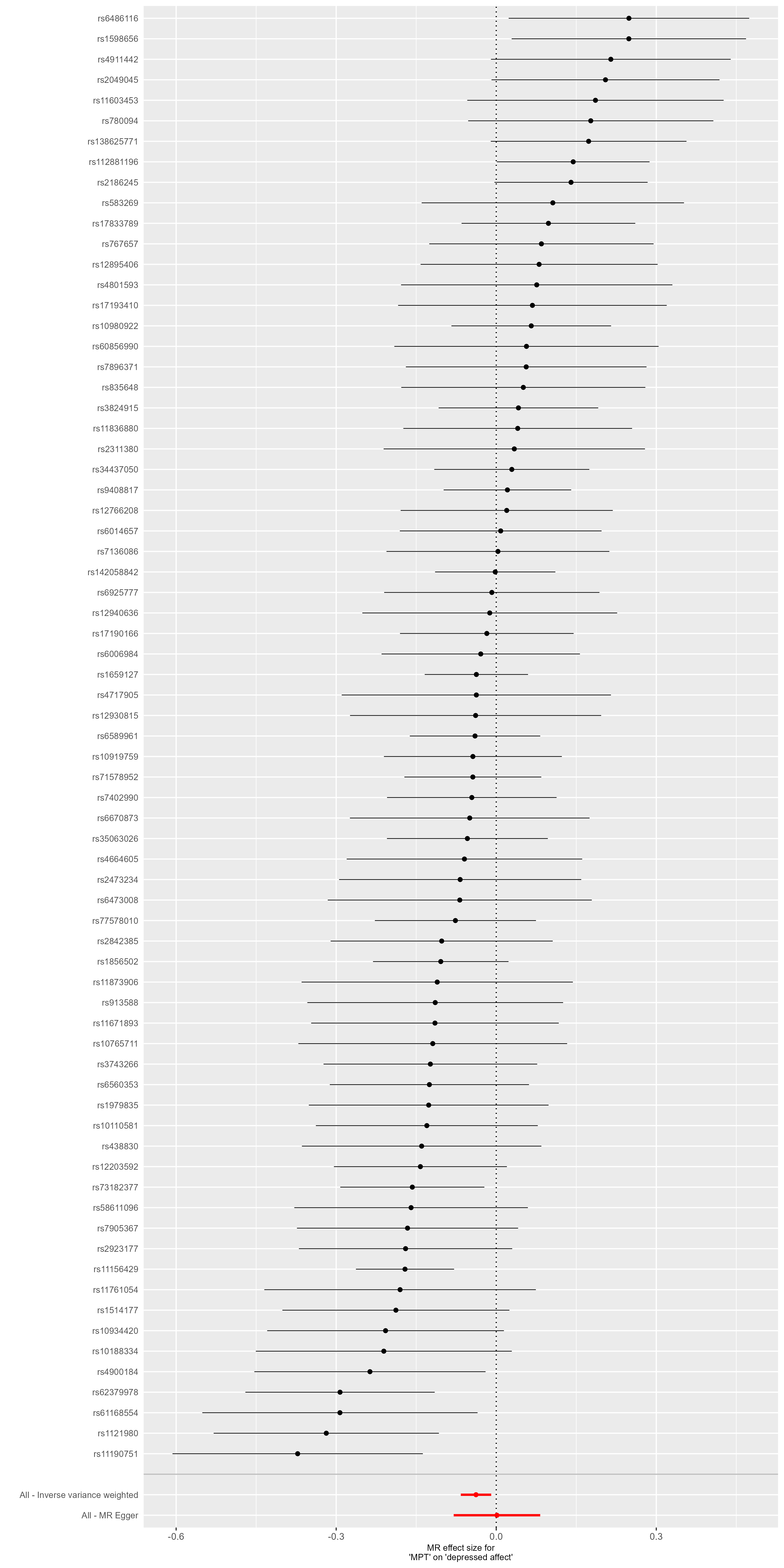


**Supplementary Figure S15 – Forest Plot - Depressed Affect.** This forest plot illustrates the results of single-SNP MR analyses with the MR effect estimates for each SNP on the outcome (‘Depressed Affect’ subdomain of Neuroticism).


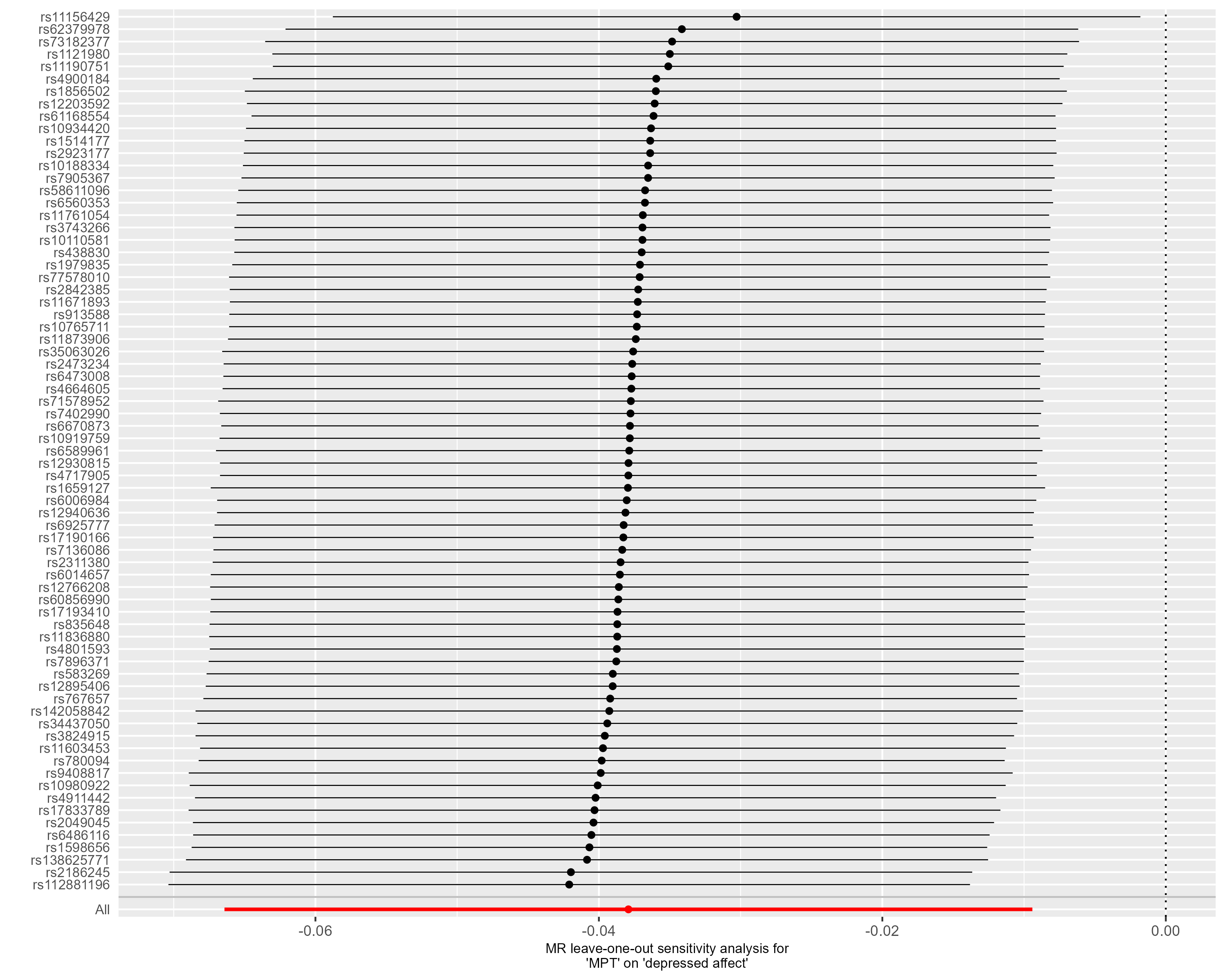


**Supplementary Figure S16 – Leave-one-out analysis - Depressed Affect.** This forest plot illustrates the results of MR analyses (IVW) following the exclusion of individual genetic variants from the instrumental variable through leave-one-out analysis.

**Outcome: Age at Onset of Depression.**

74 of the 76 target SNPs of male puberty timing were available in the outcome GWAS by [Nagel et al. (2018)](#_ENREF_16). No outlier was identified by MR-PRESSO. The uncorrected effect (p = 0.034) indicates that a younger onset of male puberty leads to an earlier onset of depression. However, this effect was not maintained after FDR correction (FDR corrected p = 0.115). The Q-statistic did not reveal significant heterogeneity (IVW: df=73, Q-statistic: 91.40, p=0.071; MR Egger: df=72, Q-statistic: 87.54, p=0.103). However, robust methods showed a mixed picture with non-significant estimates with the weighted median, the weighted mode approach, and the BMI-correct IVW estimate (Supplemental Figure S17). In line with this observation, leave-one-out-analyses revealed that after the exclusion of single variants, the 95%-CI of the effect estimate included the null effect (Supplementary Figure S21).


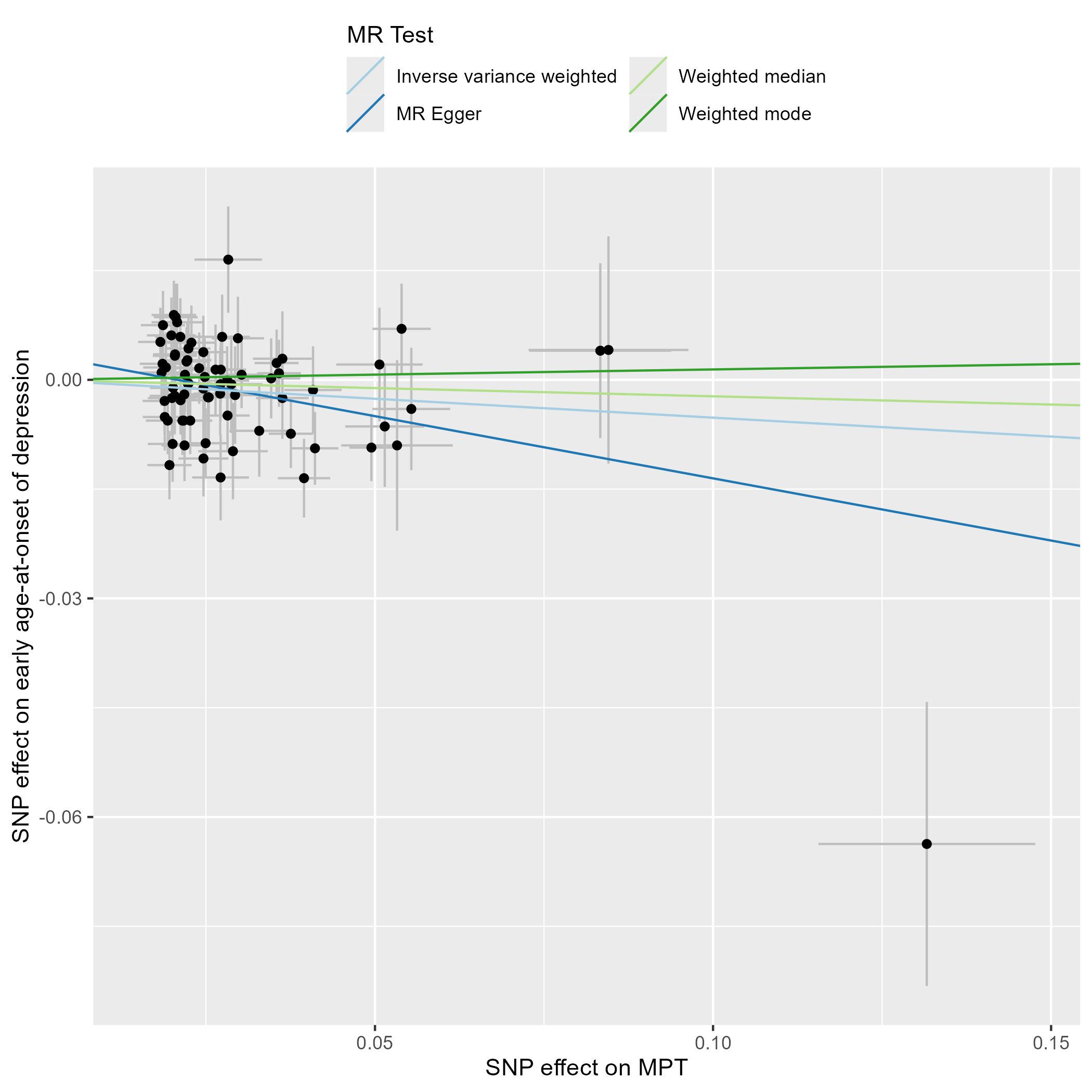


**Supplementary Figure S17 – Scatter Plot – Age at Onset of Depression.** This scatter plot depicts the effect estimates (beta, error bars represent the standard error) of each genetic variant included in the analysis on the exposure (male puberty timing ([Hollis et al., 2020](#_ENREF_9))) and the outcome (Age at Onset of Depression ([Harder et al., 2022](#_ENREF_5))).


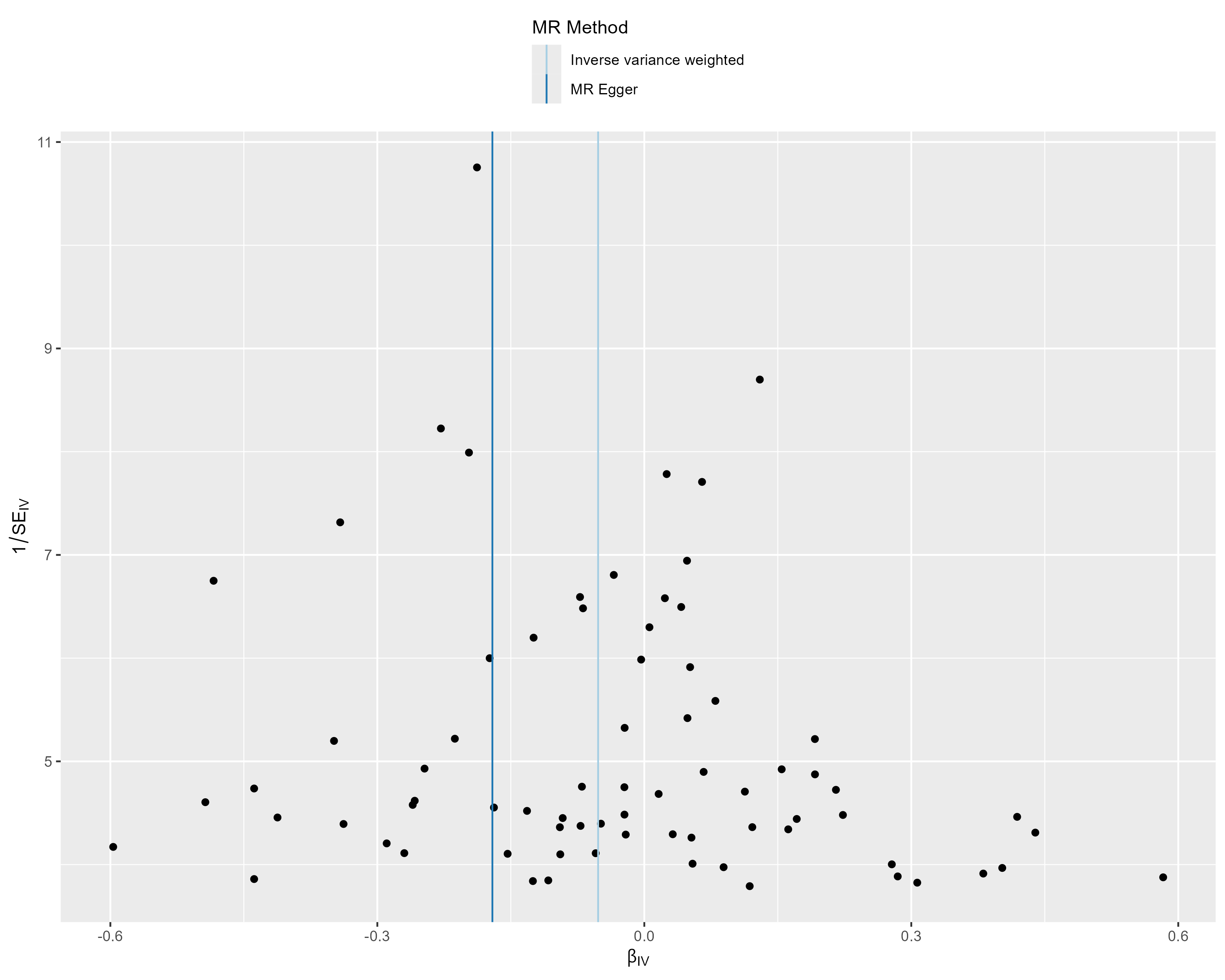


**Supplementary Figure S18 – Funnel Plot - Age at Onset of Depression.** This funnel plot illustrates the precision of each genetic variant (as measured by the inverse of the standard error (SE_IV_)) and their MR effect estimate (β_IV_).


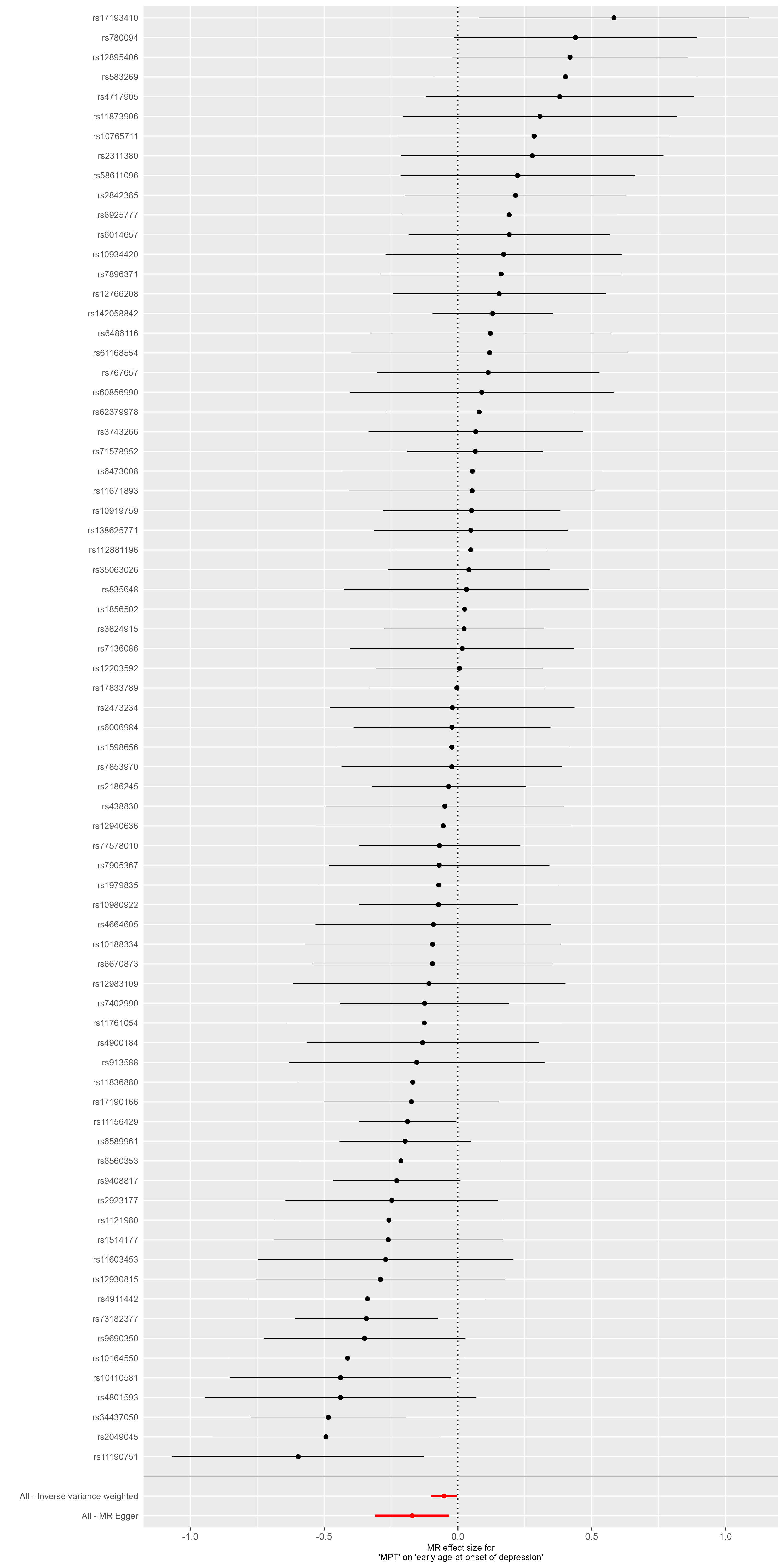


**Supplementary Figure S19 – Forest Plot - Age at Onset of Depression.** This forest plot illustrates the results of single-SNP MR analyses with the MR effect estimates for each SNP on the outcome (Age at Onset of Depression).


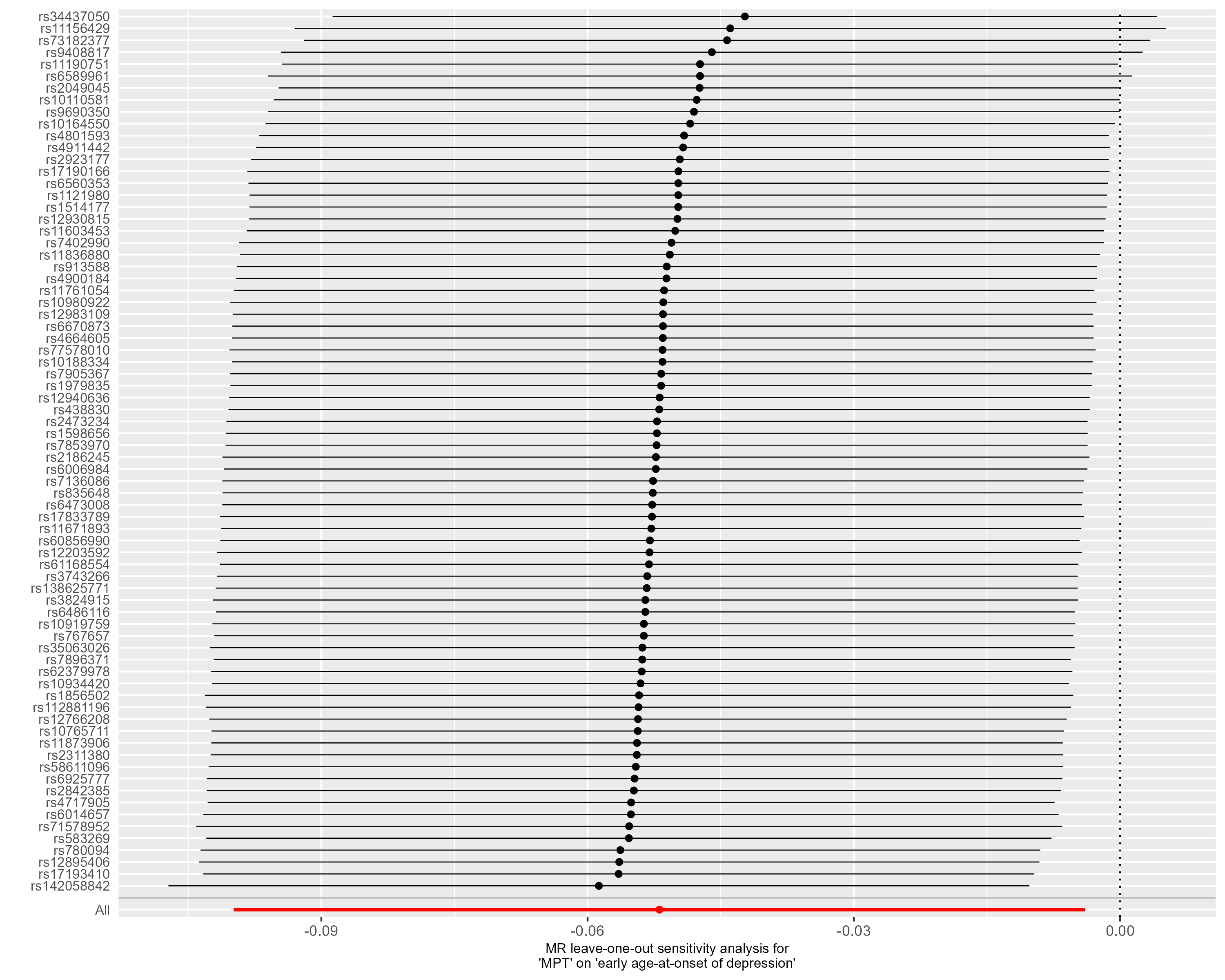


**Supplementary Figure S20 – Leave-one-out analysis - Age at Onset of Depression.** This forest plot illustrates the results of MR analyses (IVW) following the exclusion of individual genetic variants from the instrumental variable through leave-one-out analysis.


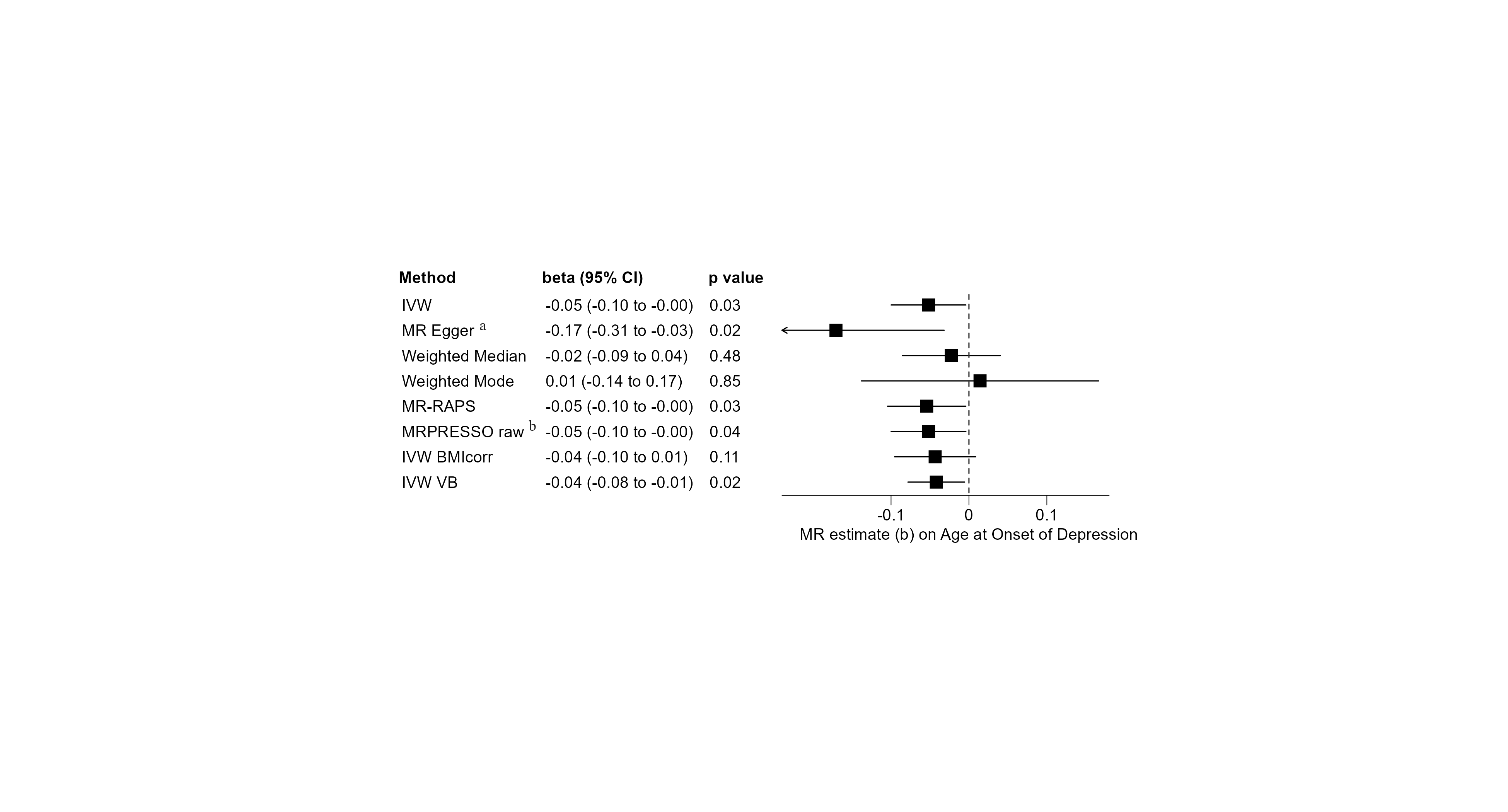


**Supplementary Figure S21 – Sensitivity Analyses – Age at Onset of Depression.** Univariable MR analyses on the effect of male puberty timing on the age at onset of depression. Negative MR estimates indicate that earlier male pubertal timing is associated with younger age at onset of depression. a: Eggers-intercept did not indicate significant directional pleiotropy (intercept=0.0035, se=0.0020, p=0.079). However, the I^2^-statistic was 0.734 and thus well below the threshold of 0.9. This suggests a violation of the NOME assumption, essential to the validity of MR Egger results. Therefore, the MR Egger result should be interpreted with caution. b: The MR-PRESSO global test for pleiotropy was not significant (RSSobs=94.12, p=0.084), and no outliers were identified by MR-PRESSO. Thus, the MR-PRESSO corrected IVW estimate (IVW) corresponds to the uncorrected IVW estimate (MR-PRESSO raw).

**Supplementary Results 2 – Details on outcomes with primary endpoint p value < 0.05**


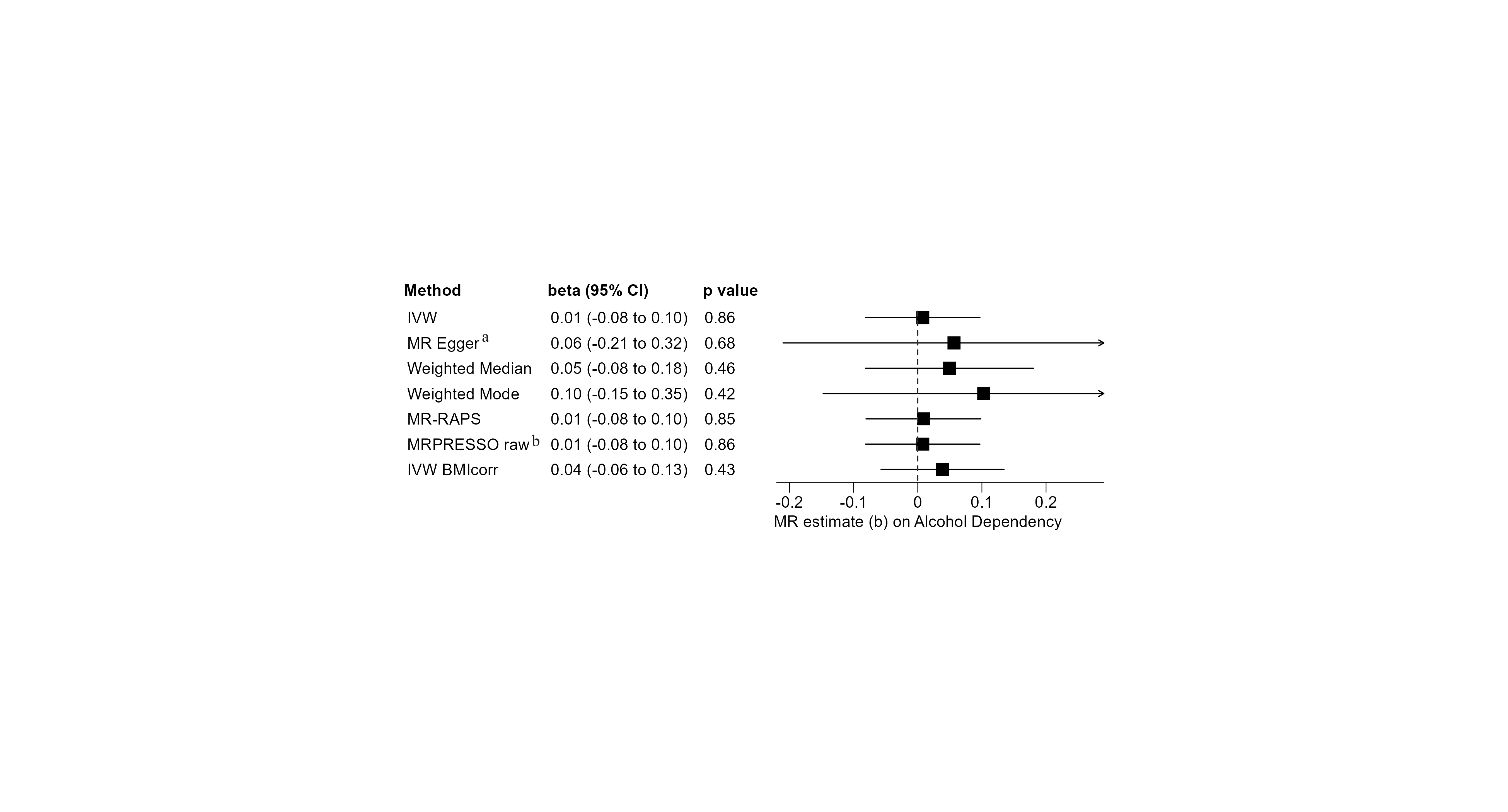


**Supplementary Figure S22 – Sensitivity Analyses – Alcohol Dependency.** Univariable MR analyses on the effect of male puberty timing on the risk of developing alcohol dependence. Seventy-five of the target SNPs for male puberty timing were covered in the outcome GWAS by [Walters et al. (2018)](#_ENREF_22). Positive MR estimates indicate that later male puberty timing is associated with higher risk of developing alcohol dependence. The Q-statistic did not reveal significant heterogeneity (IVW: df=74, Q-statistic: 82.60, p=0.231; MR Egger: df=73, Q-statistic: 82.43, p=0.211). a: Eggers-intercept did not indicate significant directional pleiotropy (intercept= -0.0015, se= 0.0038, p=0.705). The I^2^-statistic was 0.787, indicating a violation of the NOME assumption. b: The MR-PRESSO global test for pleiotropy was not significant (RSSobs=84.70, p=0.228), and no outliers were identified by the MR-PRESSO. Thus, the MR-PRESSO corrected IVW estimate (IVW) corresponds to the uncorrected IVW estimate (MR-PRESSO raw).


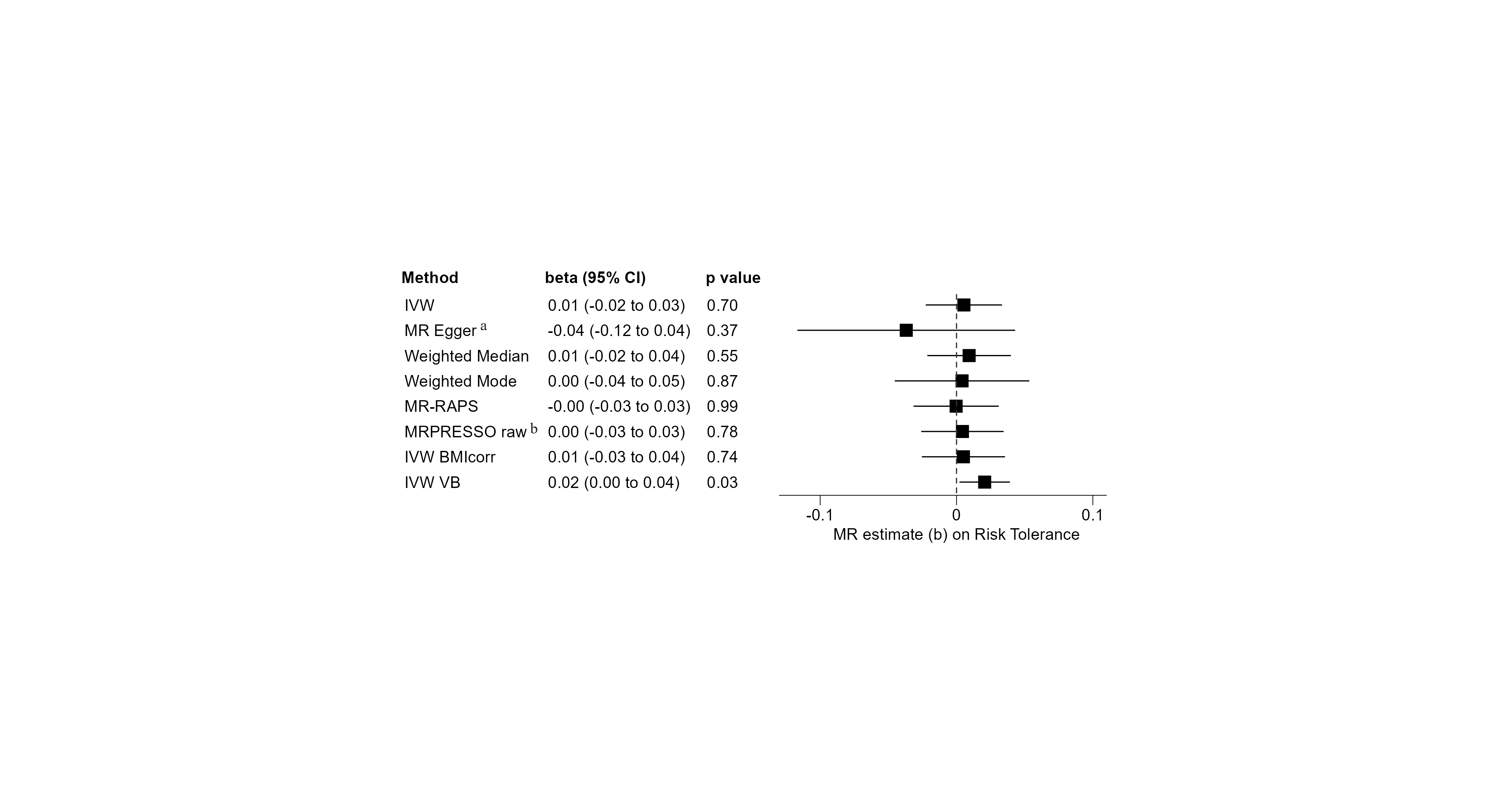


**Supplementary Figure S23 – Sensitivity Analyses – Risk Tolerance.** Univariable MR analyses on the effect of male puberty timing on general risk tolerance. 75 of the 76 target SNPs for male puberty timing were covered in the outcome GWAS by [Karlsson Linnér et al. (2019)](#_ENREF_13). Positive MR estimates indicate that later male puberty timing is associated with greater tolerance towards risky behavior. The Q-statistic revealed significant heterogeneity (IVW: df=72, Q-statistic: 147.20, p= 4x10^-7^; MR Egger: df=71, Q-statistic: 144.68, p=5x10^-7^). a: Eggers-intercept did not indicate significant directional pleiotropy (intercept= 0.0013, se= 0.0012, p=0.270). The I^2^-statistic was 0.747, and thus well below the threshold of 0.9, indicating a violation of the NOME assumption. Therefore, the MR Egger result should be interpreted with caution. b: The MR-PRESSO global test for pleiotropy was significant (RSSobs=184.55, p<3x10^-4^), and two outliers were identified by MR-PRESSO. Thus, the instrumental variable consisted of 73 SNPs. As the effect estimate based on the exposure ‘Voice Break’ indicated an effect on ‘Risk Tolerance’, additional sensitivity analyses were undertaken with ‘Voice Break’ as an exposure (not shown in the figure), all with non-significant findings: MR-PRESSO raw (beta=0.02, 95%-CI [-0.01, 0.04], p=0.188), MR Egger (beta=-0.01, 95%-CI [-0.04, 0.06], p=0.677), Weighted Median (beta=0.01, 95%-CI [-0.01, 0.03], p=0.450), and Weighted Mode (beta=0.00, 95%-CI [-0.03, 0.04], p=0.829).


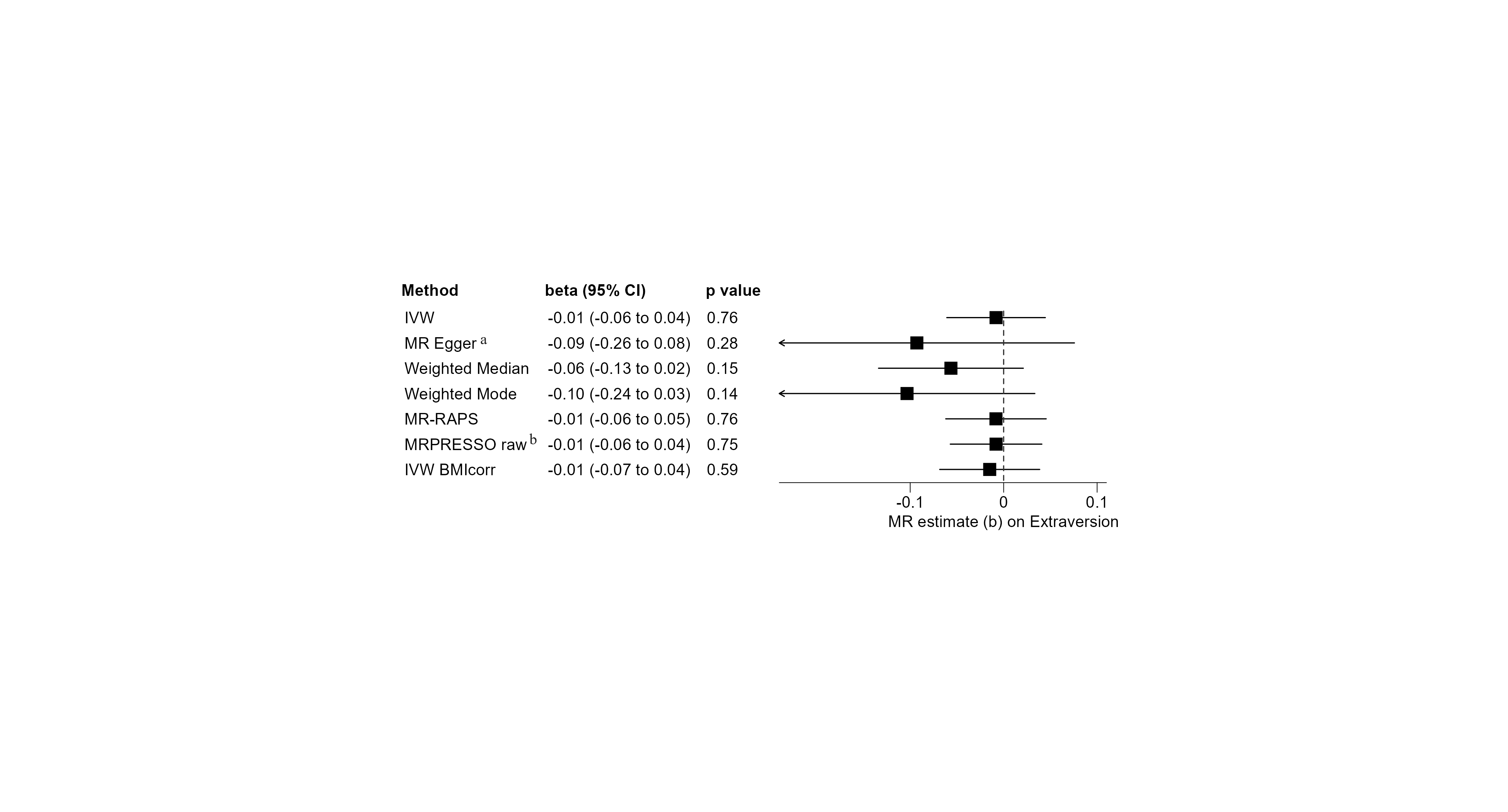


**Supplementary Figure S24 – Sensitivity Analyses – Extraversion.** Univariable MR analyses on the effect of male puberty timing on ‘Extraversion’. 73 of the 76 target SNPs for male puberty timing were covered in the outcome GWAS by [Van den Berg et al. (2016)](#_ENREF_20). Positive MR estimates indicate that later male puberty timing is associated with greater expression of the character trait ‘Extraversion’. The Q-statistic did not reveal significant heterogeneity (IVW: df=72, Q-statistic: 62.12, p=0.790; MR Egger: df=71, Q-statistic: 61.05, p=0.794). a: Eggers-intercept did not indicate significant directional pleiotropy (intercept= 0.0025, se=0.0024, p=0.303). The I^2^-statistic was 0.917, indicating no major violation of the NOME assumption for the conduction of MR Egger. b: The MR-PRESSO global test for pleiotropy was not significant (RSSobs=63.92, p=0.790), and no outliers were identified by MR-PRESSO. Thus, the MR-PRESSO corrected IVW estimate (IVW) corresponds to the uncorrected IVW estimate (MR-PRESSO raw).


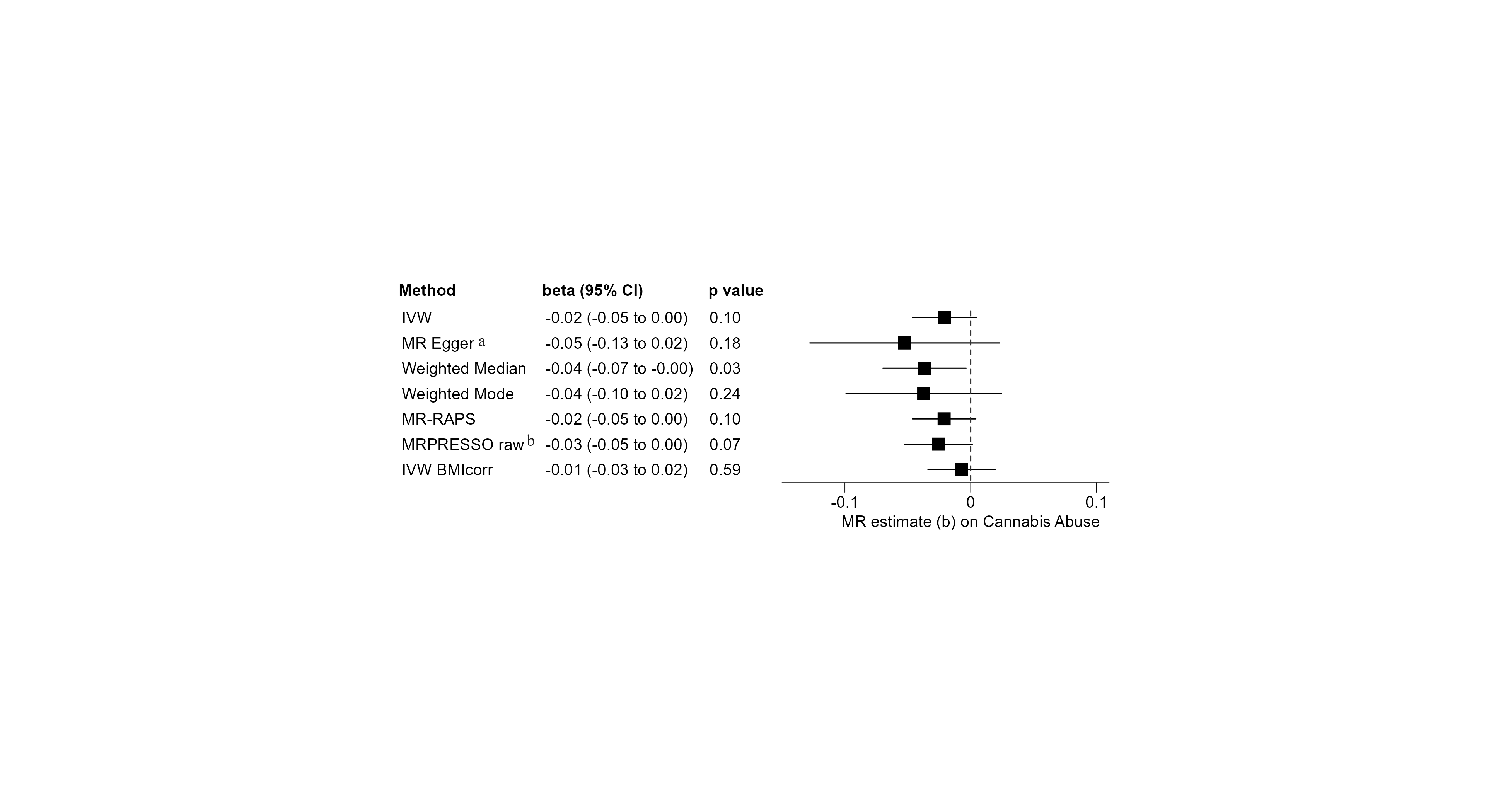


**Supplementary Figure S25 – Sensitivity Analyses – Cannabis Abuse.** Univariable MR analyses on the effect of male puberty timing on ‘Cannabis Abuse’. All 76 target SNPs for male puberty timing were covered in the outcome GWAS by [Johnson et al. (2020)](#_ENREF_12). Positive MR estimates indicate that later male puberty timing is associated with a higher risk of developing cannabis abuse. The Q-statistic did reveal a small amount of, however significant, heterogeneity (IVW: df=74, Q-statistic: 96.80, p=0.039; MR Egger: df=73, Q-statistic: 95.79, p=0.038). a: Eggers-intercept did not indicate significant directional pleiotropy (intercept=0.0009, se=0.0011, p=0.386). The I^2^-statistic was 0.783, indicating a relevant violation of the NOME assumption for the conduction of MR Egger. b: The MR-PRESSO global test for pleiotropy was significant (RSSobs=115.37, p=0.003), and one outlier was identified by the MR-PRESSO method. Thus, the instrumental variable consisted of 75 SNPs.


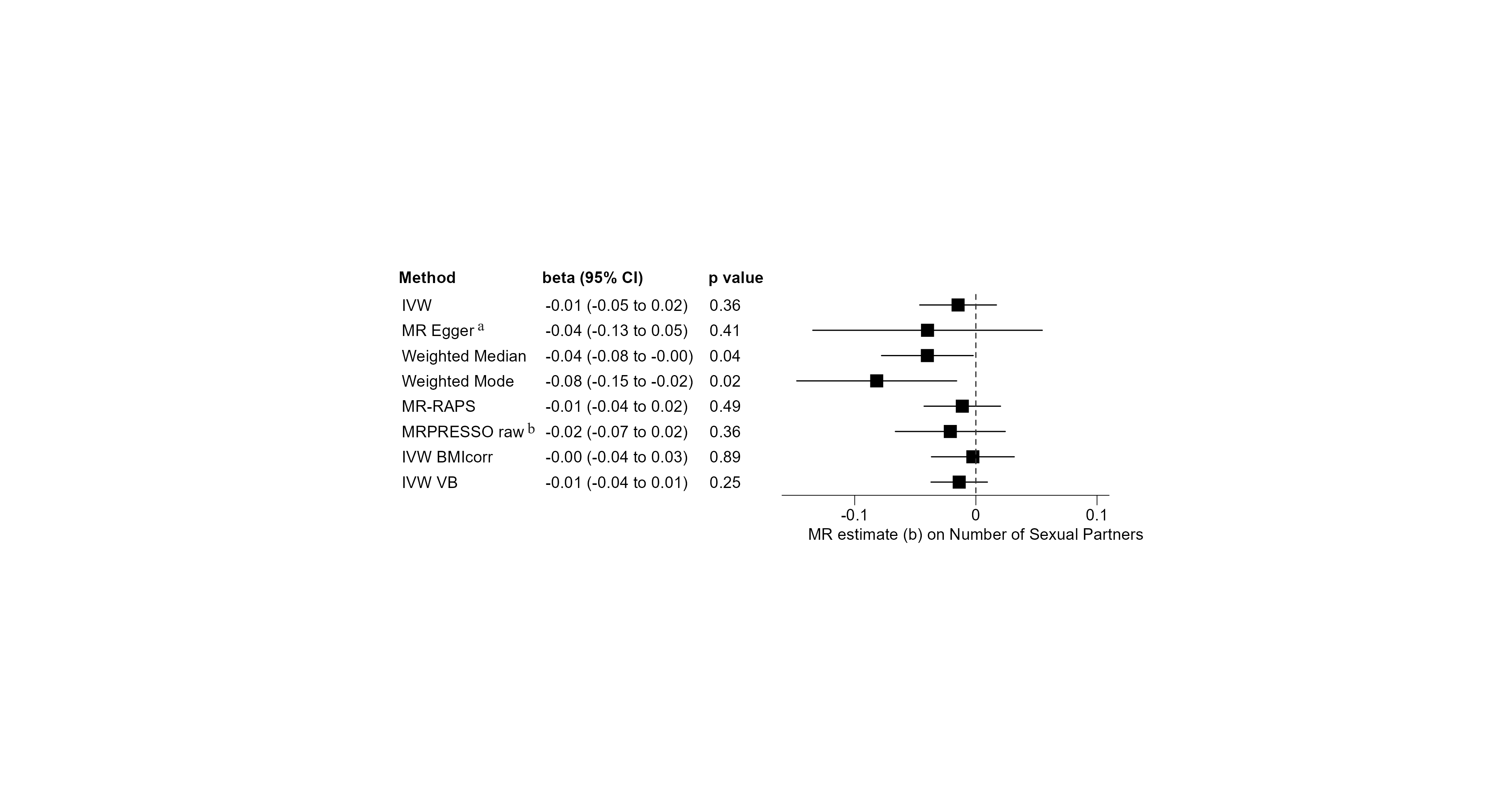


**Supplementary Figure S26 – Sensitivity Analyses – Number of Sexual Partners.** Univariable MR analyses on the effect of male puberty timing on the total number of sexual partners. 75 of the 76 target SNPs for male puberty timing were covered in the outcome GWAS by [Karlsson Linnér et al. (2019)](#_ENREF_13). Positive MR estimates indicate that later male puberty timing is associated with a larger number of total sexual partners. The Q-statistic did reveal significant heterogeneity (IVW: df=64, Q-statistic: 117.16, p=5x10^-5^; MR Egger: df=63, Q-statistic: 116.60, p=4x10^-4^). a: Eggers-intercept did not indicate significant directional pleiotropy (intercept=0.0075, se=0.00135, p=0.581). The I^2^-statistic was 0.753, and thus well below the threshold of 0.9, indicating a violation of the NOME assumption. Therefore, the MR Egger result should be interpreted with caution. b: The MR-PRESSO global test for pleiotropy was significant (RSSobs=338.26, p<3x10^-4^) and ten outliers were identified by the MR-PRESSO. Thus, the instrumental variable consisted of 65 SNPs.


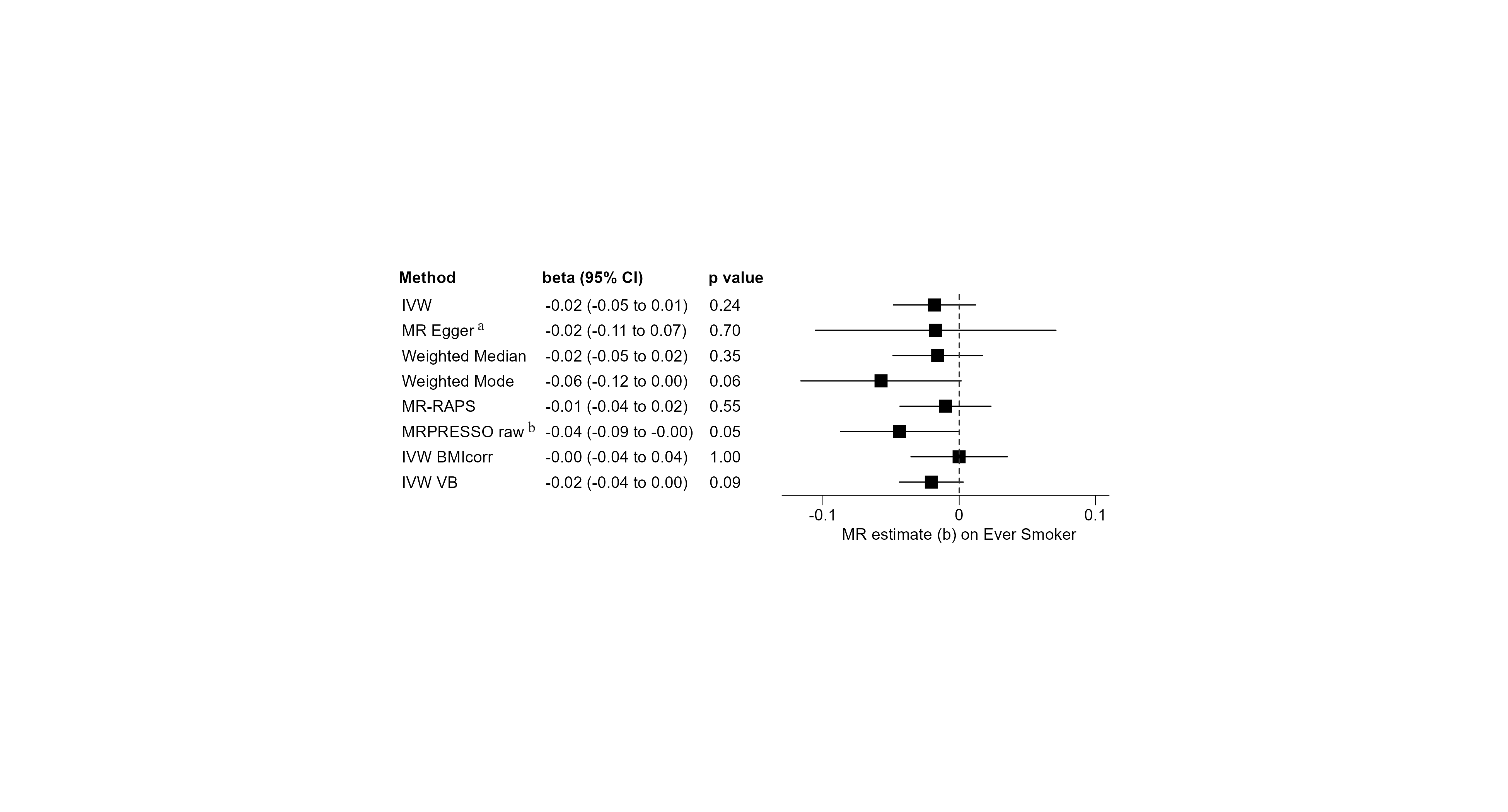


**Supplementary Figure S27 – Sensitivity Analyses – Ever Smoker.** Univariable MR analyses on the effect of male puberty timing on reporting to have ever smoked. 75 of the 76 target SNPs for male puberty timing were covered in the outcome GWAS by [Karlsson Linnér et al. (2019)](#_ENREF_13). Positive MR estimates indicate that later male puberty timing is associated with a higher risk of ever initiating smoking. The Q-statistic did reveal significant heterogeneity (IVW: df=66, Q-statistic: 149.32, p=2x10^-8^; MR Egger: df=65, Q-statistic: 149.32, p=1x10^-8^). a: Eggers-intercept did not indicate significant directional pleiotropy (intercept=0.0000, se=0.0013, p=0.983). The I^2^-statistic was 0.744, and thus well below the threshold of 0.9, indicating a violation of the NOME assumption. Therefore, the MR Egger result should be interpreted with caution. b: The MR-PRESSO global test for pleiotropy was significant (RSSobs=386.07, p<3x10^-4^), and eight outliers were identified by MR-PRESSO. Thus, the instrumental variable consisted of 67 SNPs. As the IVW estimate without exclusion of pleiotropic variants (MR-PRESSO raw) yielded a (borderline) significant result (p=0.049992), additional sensitivity analyses were undertaken without exclusion of pleiotropic variants, all with non-significant findings (not shown in the figure): MR Egger (beta=-0.03, 95%-CI [-0.16, 0.09], p=0.607), weighted Median (beta=-0.02, 95%-CI [-0.05, 0.01], p=0.275), and Weighted Mode (beta=-0.05, 95%-CI[-0.11, 0.01], p=0.125).


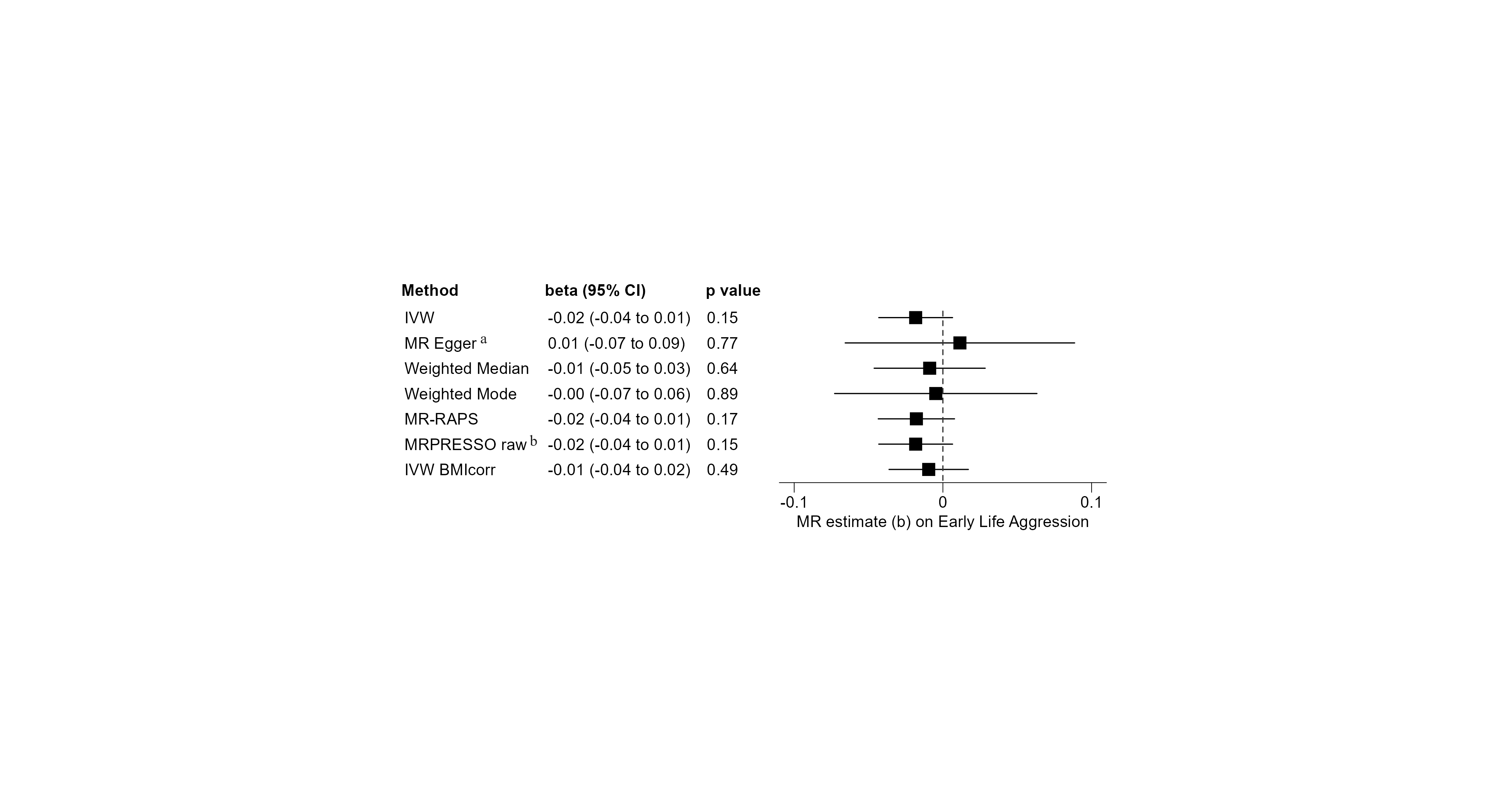


**Supplementary Figure S28 – Sensitivity Analyses – Early Life Aggression.** Univariable MR analyses on the effect of male puberty timing on aggression in children and adolescents. 74 of the 76 target SNPs for male puberty timing were covered in the outcome GWAS by [Ip et al. (2021)](#_ENREF_10). Positive MR estimates indicate that later male puberty timing is associated with a higher rating of aggression in childhood and adolescence. The Q-statistic did not reveal significant heterogeneity (IVW: df=73, Q-statistic: 76.98, p=0.352; MR Egger: df=72, Q-statistic: 76.30, p=0.342). a: Eggers-intercept did not indicate significant directional pleiotropy (intercept=-0.0009, se=0.0011, p=0.427). I^2^-statistic was 0.826, and thus below the threshold of 0.9, indicating a violation of the NOME assumption for the conduction of MR Egger. Therefore, the MR Egger result should be interpreted with caution. b: The MR-PRESSO global test for pleiotropy was not significant (RSSobs=79.07, p=0.379), and no outlier was identified by MR-PRESSO. Thus, the MR-PRESSO corrected IVW estimate (IVW) corresponds to the uncorrected IVW estimate (MR-PRESSO raw).


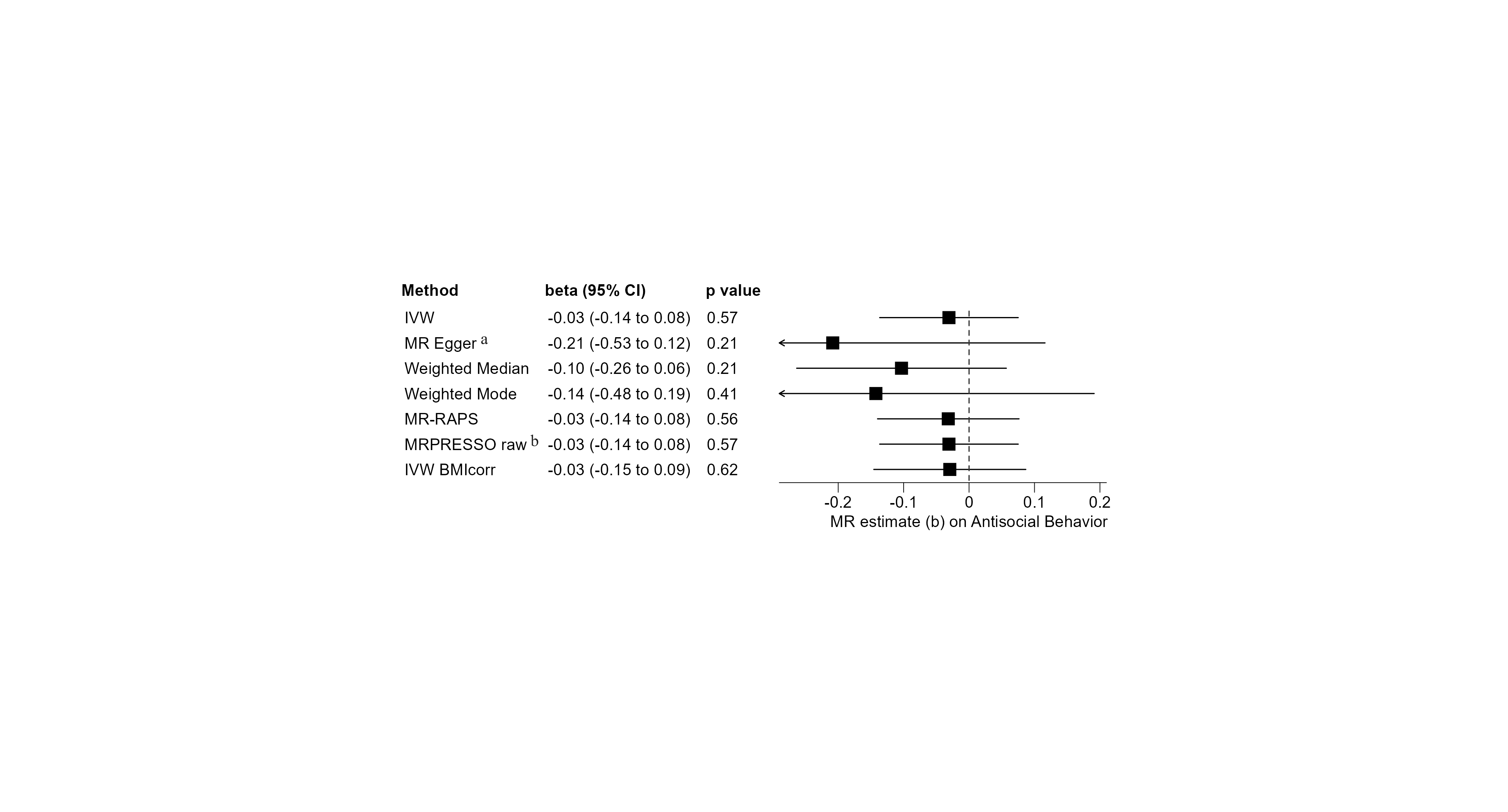


**Supplementary Figure S29 – Sensitivity Analyses – Antisocial Behavior.** Univariable MR analyses on the effect of male puberty timing on ‘Antisocial Behavior’. 75 of the 76 target SNPs for male puberty timing were covered in the outcome GWAS by [Tielbeek et al. (2017)](#_ENREF_19). Positive MR estimates indicate that later male puberty timing is associated with more antisocial behavior. The Q-statistic did not reveal significant heterogeneity (IVW: df=74, Q-statistic: 75.70, p=0.423; MR Egger: df=73, Q-statistic: 74.39, p=0.433). a: Eggers-intercept did not indicate significant directional pleiotropy (intercept=0.0053, se=0.0046, p=0.260). The I^2^-statistic was 0.822, indicating a relevant violation of the NOME assumption for the conduction of MR Egger. b: The MR-PRESSO global test for pleiotropy was not significant (RSSobs=78.21, p=0.411), and no outlier was identified by MR-PRESSO. Thus, the MR-PRESSO corrected IVW estimate (IVW) corresponds to the uncorrected IVW estimate (MR-PRESSO raw).


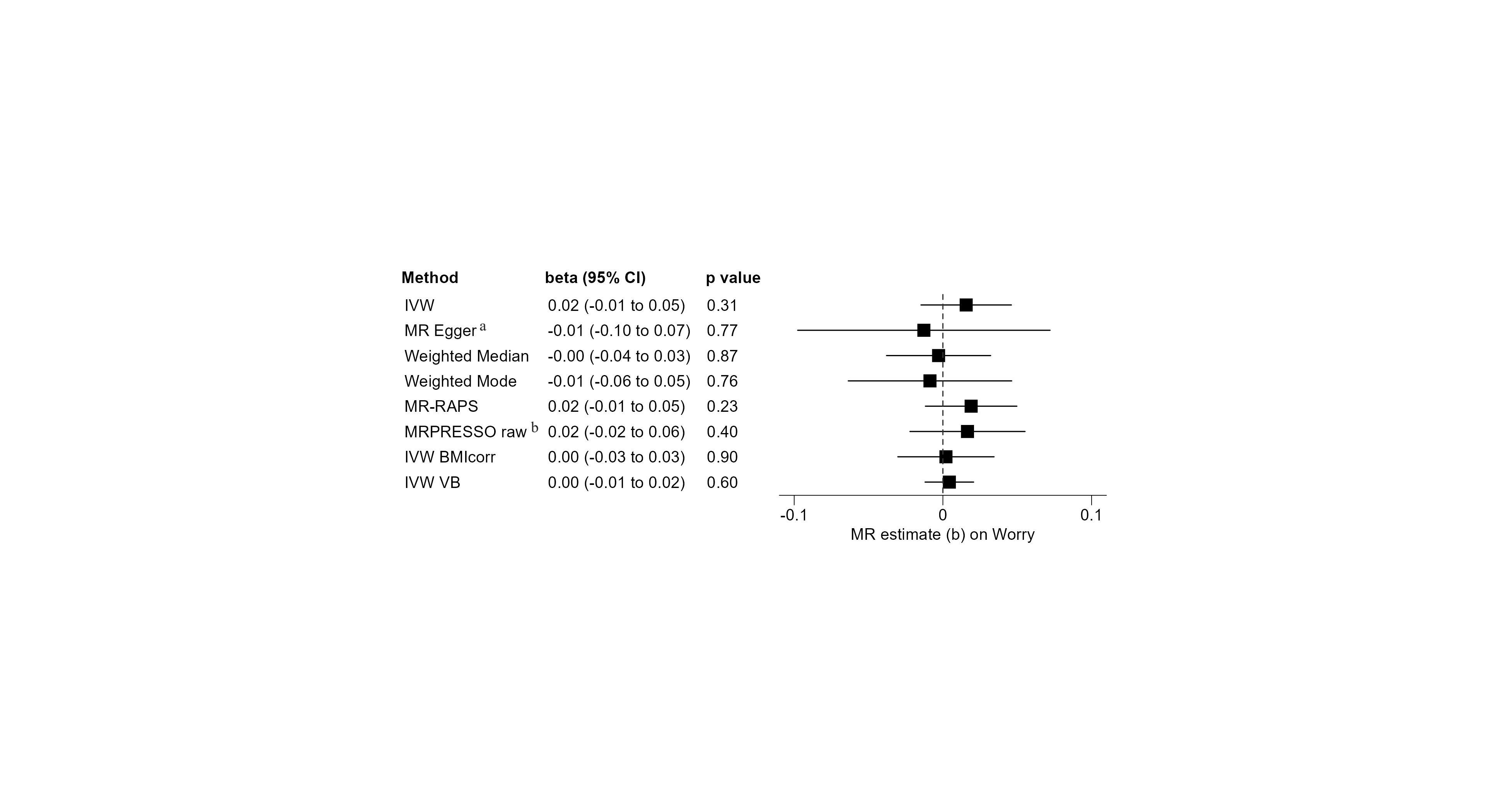


**Supplementary Figure S30 – Sensitivity Analyses – Worry.** Univariable MR analyses on the effect of male puberty timing on the ‘Worry’ subdomain of neuroticism. 72 of the 76 target SNPs for male puberty timing were covered in the outcome GWAS by [Nagel et al. (2018)](#_ENREF_16). Positive MR estimates indicate that later male puberty timing is associated with higher expression on the ‘Worry’ subdomain of neuroticism. The Q-statistic did reveal significant heterogeneity (IVW: df=66, Q-statistic: 118.80, p=7.3x10^-5^; MR Egger: df=65, Q-statistic: 117.90, p=6.6x10^-5^). a: Eggers-intercept did not indicate significant directional pleiotropy (intercept=0.0009, se=0.0012, p=0.484). The I^2^-statistic was 0.792, and thus below the threshold of 0.9, indicating a violation of the NOME assumption. Therefore, the MR Egger result should be interpreted with caution. b: The MR-PRESSO global test for pleiotropy was significant (RSSobs=224.89, p<3x10^-4^), and five outliers were identified by MR-PRESSO. Thus, the instrumental variable consisted of 67 SNPs.


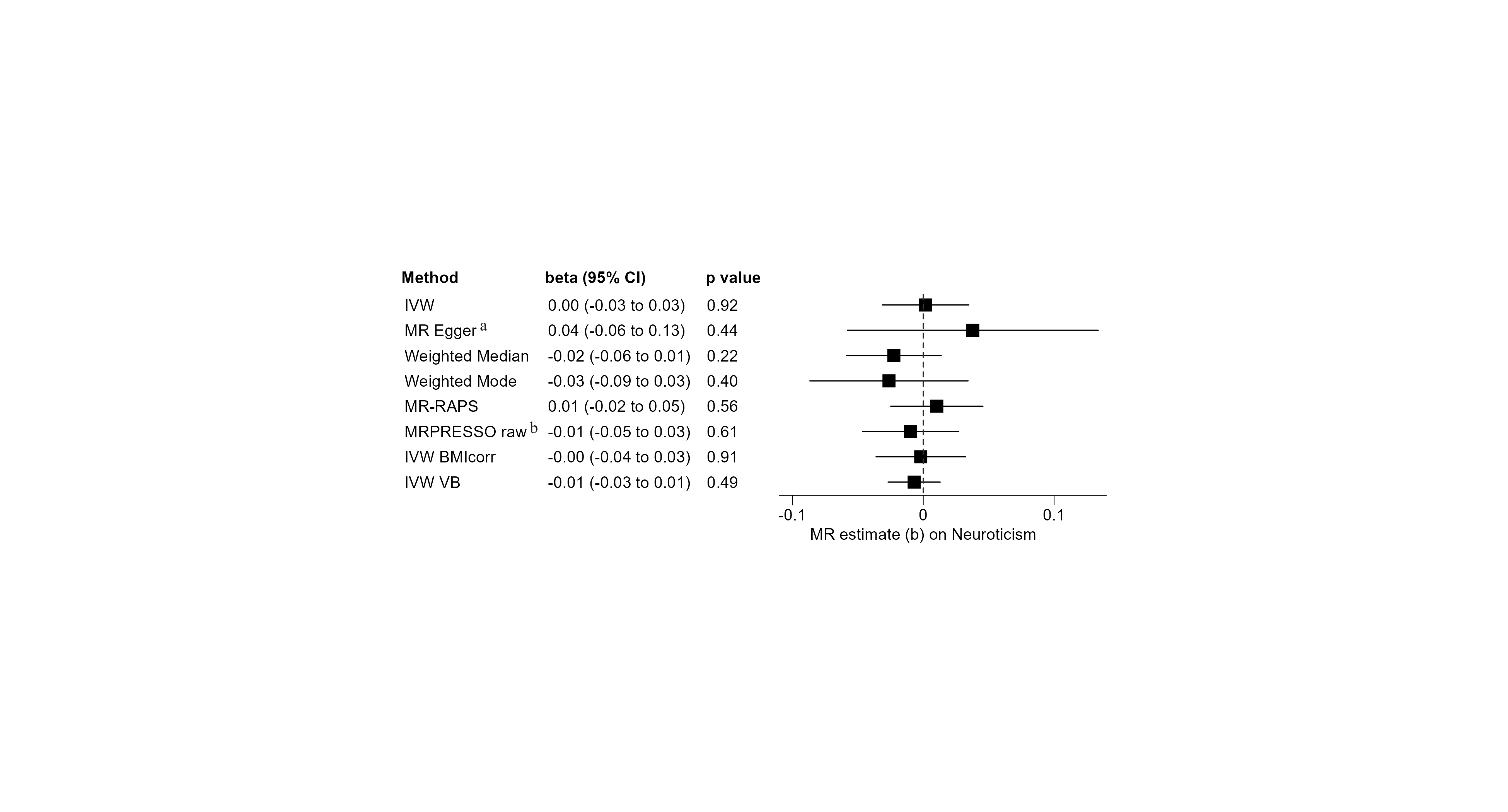


**Supplementary Figure S31 – Sensitivity Analyses – Neuroticism.** Univariable MR analyses on the effect of male puberty timing on ‘Neuroticism’. 72 of the 76 target SNPs for male puberty timing were covered in the outcome GWAS by [Nagel et al. (2018)](#_ENREF_16). Positive MR estimates indicate that later male puberty timing is associated with higher expression of neuroticism. The Q-statistic did reveal significant heterogeneity (IVW: df=68, Q-statistic: 152.11, p= 2.2x10^-8^; MR Egger: df=67, Q-statistic: 150.72, p=2.2x10^-8^). a: Eggers-intercept did not indicate significant directional pleiotropy (intercept=-0.0011, se=0.0014, p=0.434). The I^2^-statistic was 0.732, indicating a relevant violation of the NOME assumption. b: The MR-PRESSO global test for pleiotropy was significant (RSSobs=218.53, p<3x10^-4^), and three outliers were identified by MR-PRESSO. Thus, the instrumental variable consisted of 69 SNPs.


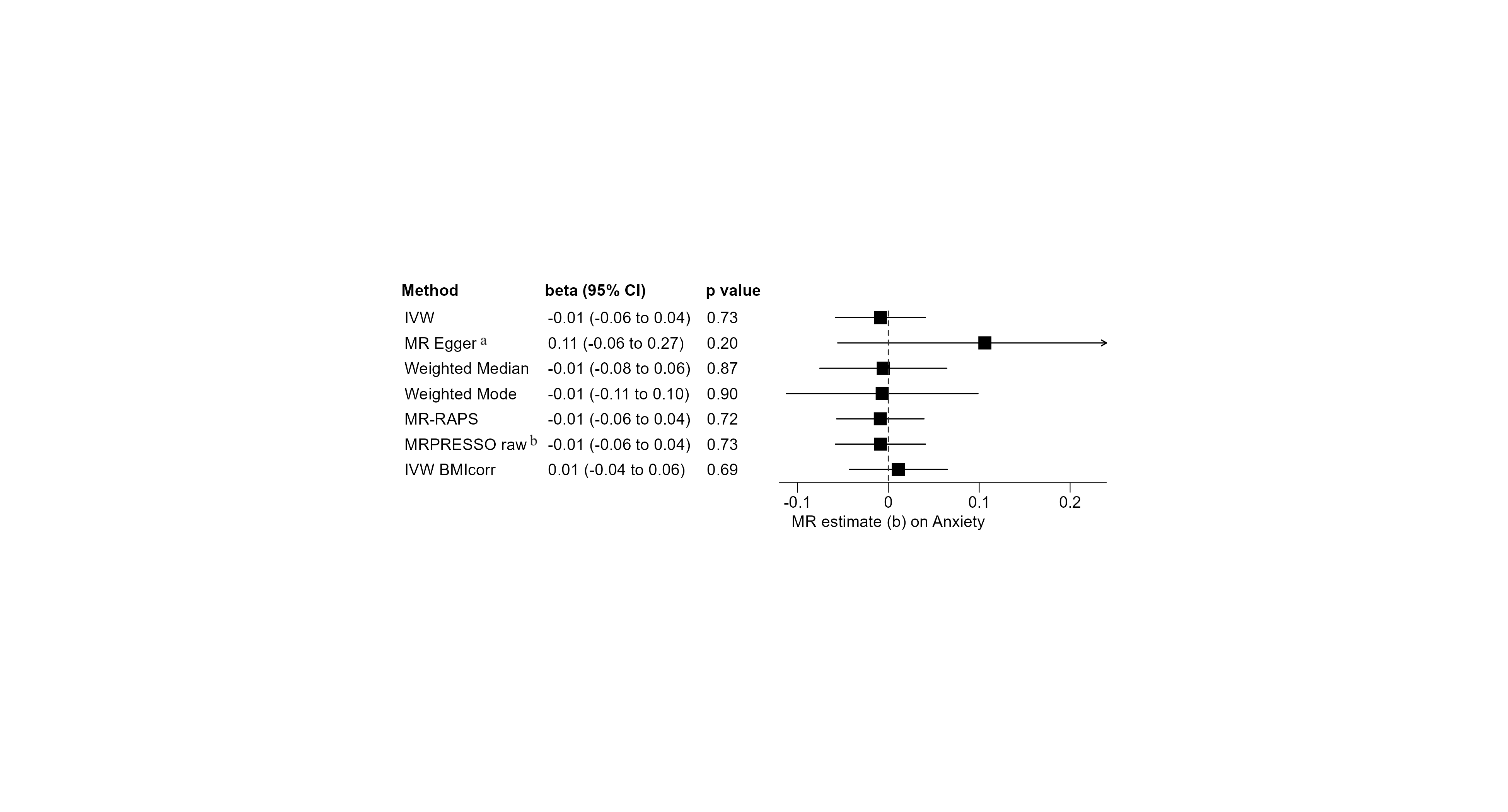


**Supplementary Figure S32 – Sensitivity Analyses – Anxiety Disorders.** Univariable MR analyses on the effect of male puberty timing on the development and/or severity of anxiety disorders (factor score). 73 of the 76 target SNPs for male puberty timing were covered in the outcome GWAS by [Otowa et al. (2016)](#_ENREF_17). Positive MR estimates indicate that later male puberty timing is associated with a higher risk for and/or higher disease severity of anxiety disorders. The Q-statistic did not reveal significant heterogeneity (IVW: df=72, Q-statistic: 79.22, p=0.262; MR Egger: df=71, Q-statistic: 76.91, p= 0.295). a: Eggers-intercept did not indicate significant directional pleiotropy (intercept=-0.0034, se=0.0023, p=0.149). The I^2^-statistic was 0.937, indicating no major violation of the NOME assumption. b: The MR-PRESSO global test for pleiotropy was not significant (RSSobs=80.90, p=0.278), and no outlier was identified by MR-PRESSO. Thus, the MR-PRESSO corrected IVW estimate (IVW) corresponds to the uncorrected IVW estimate (MR-PRESSO raw).


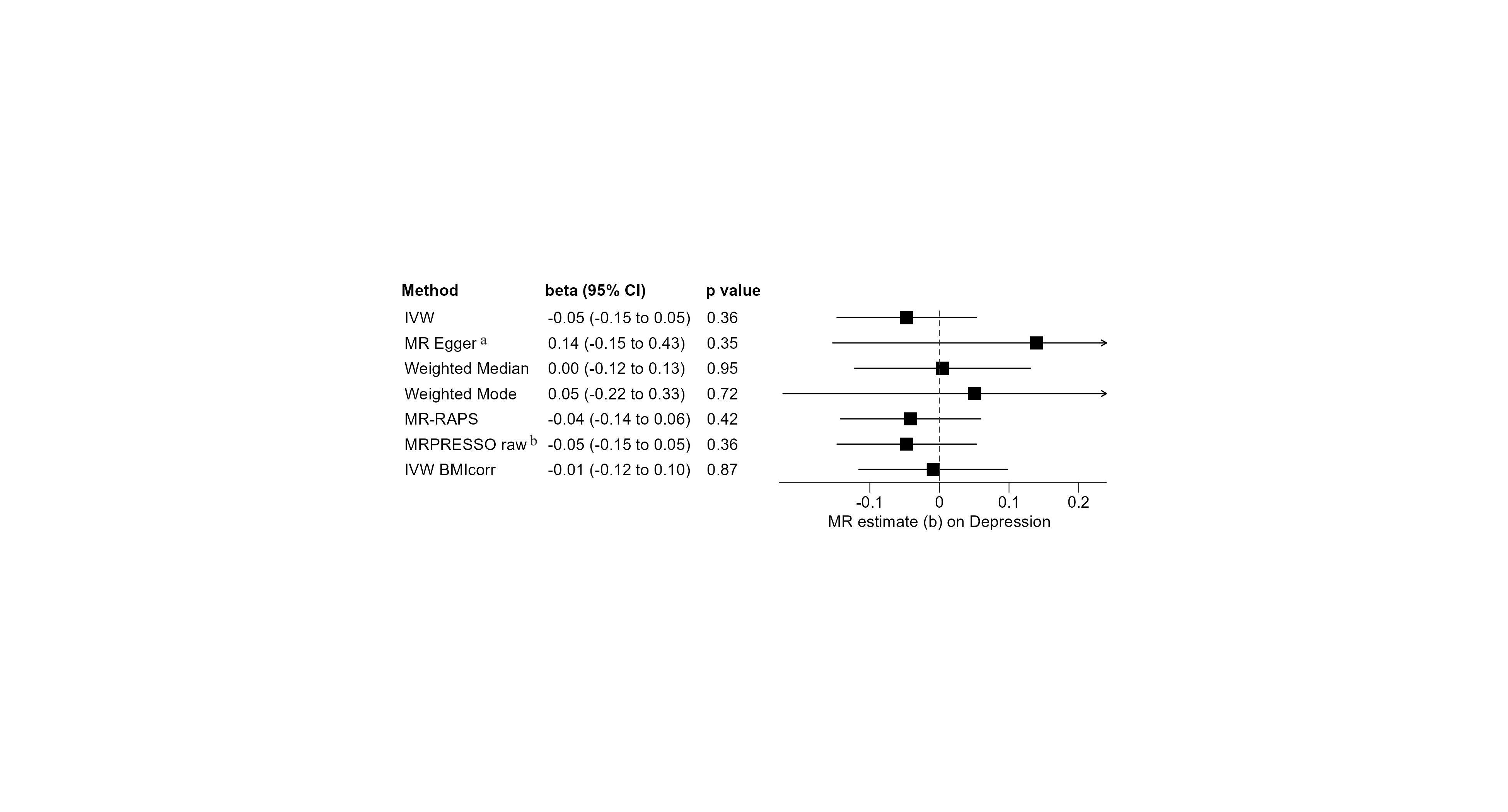


**Supplementary Figure S33 – Sensitivity Analyses – Depression.** Univariable MR analyses on the effect of male puberty timing on the risk of developing a major depressive disorder. 76 of the 76 target SNPs for male puberty timing were covered in the outcome GWAS by [Wray et al. (2018)](#_ENREF_24). Positive MR estimates indicate that later male puberty timing is associated with a higher risk for developing depression. The Q-statistic did reveal significant heterogeneity (IVW: df=75, Q-statistic: 110.26, p=0.005; MR Egger: df=74, Q-statistic: 107.70, p=0.006). a: Eggers-intercept did not indicate significant directional pleiotropy (intercept=-0.0056, se=0.0042, p=0.189). The I^2^-statistic was 0.776, and thus below the threshold of 0.9, indicating a violation of the NOME assumption. Therefore, the MR Egger result should be interpreted with caution. b: The MR-PRESSO global test for pleiotropy was significant (RSSobs=113.16, p=0.006). However, no significant outlier was identified by MR-PRESSO. Thus, the MR-PRESSO corrected IVW estimate (IVW) corresponds to the uncorrected IVW estimate (MR-PRESSO raw).

### Supplementary References

Bowden, Jack, Davey Smith, George, & Burgess, Stephen. (2015). Mendelian randomization with invalid instruments: effect estimation and bias detection through Egger regression. *International journal of epidemiology, 44*(2), 512-525.

Bowden, Jack, Davey Smith, George, Haycock, Philip C, & Burgess, Stephen. (2016). Consistent estimation in Mendelian randomization with some invalid instruments using a weighted median estimator. *Genetic epidemiology, 40*(4), 304-314.

Bowden, Jack, Del Greco M, Fabiola, Minelli, Cosetta, Davey Smith, George, Sheehan, Nuala A, & Thompson, John R. (2016). Assessing the suitability of summary data for two-sample Mendelian randomization analyses using MR-Egger regression: the role of the I 2 statistic. *International journal of epidemiology, 45*(6), 1961-1974.

Burgess, Stephen, Butterworth, Adam, & Thompson, Simon G. (2013). Mendelian randomization analysis with multiple genetic variants using summarized data. *Genetic epidemiology, 37*(7), 658-665.

Harder, Arvid, Nguyen, Thuy-Dung, Pasman, Joëlle A, Mosing, Miriam A, Hägg, Sara, & Lu, Yi. (2022). Genetics of age-at-onset in major depression. *Translational Psychiatry, 12*(1), 124.

Hartwig, Fernando Pires, Davey Smith, George, & Bowden, Jack. (2017). Robust inference in summary data Mendelian randomization via the zero modal pleiotropy assumption. *International journal of epidemiology, 46*(6), 1985-1998.

Haycock, Philip C, Borges, Maria Carolina, Burrows, Kimberley, Lemaitre, Rozenn N, Harrison, Sean, Burgess, Stephen, . . . Tsilidis, Kostas K. (2023). Design and quality control of large-scale two-sample Mendelian randomization studies. *International journal of epidemiology, 52*(5), 1498-1521.

Hemani, Gibran, Zheng, Jie, Elsworth, Benjamin, Wade, Kaitlin H, Haberland, Valeriia, Baird, Denis, . . . Langdon, Ryan. (2018). The MR-Base platform supports systematic causal inference across the human phenome. *Elife, 7*, e34408.

Hollis, Ben, Day, Felix R, Busch, Alexander S, Thompson, Deborah J, Soares, Ana Luiza G, Timmers, Paul RHJ, . . . Perry, John RB. (2020). Genomic analysis of male puberty timing highlights shared genetic basis with hair colour and lifespan. *Nature communications, 11*(1), 1536.

Ip, Hill F, Van der Laan, Camiel M, Krapohl, Eva ML, Brikell, Isabell, Sánchez-Mora, Cristina, Nolte, Ilja M, . . . Zafarmand, Hadi. (2021). Genetic association study of childhood aggression across raters, instruments, and age. *Translational psychiatry, 11*(1), 413.

Jami, Eshim S, Hammerschlag, Anke R, Ip, Hill F, Allegrini, Andrea G, Benyamin, Beben, Border, Richard, . . . Lu, Yi. (2022). Genome-wide association meta-analysis of childhood and adolescent internalizing symptoms. *Journal of the American Academy of Child & Adolescent Psychiatry, 61*(7), 934-945.

Johnson, Emma C, Demontis, Ditte, Thorgeirsson, Thorgeir E, Walters, Raymond K, Polimanti, Renato, Hatoum, Alexander S, . . . Clarke, Toni-Kim. (2020). A large-scale genome-wide association study meta-analysis of cannabis use disorder. *The Lancet Psychiatry, 7*(12), 1032-1045.

Karlsson Linnér, Richard, Biroli, Pietro, Kong, Edward, Meddens, S Fleur W, Wedow, Robbee, Fontana, Mark Alan, . . . Hammerschlag, Anke R. (2019). Genome-wide association analyses of risk tolerance and risky behaviors in over 1 million individuals identify hundreds of loci and shared genetic influences. *Nature genetics, 51*(2), 245-257.

Karlsson Linnér, Richard, Mallard, Travis T, Barr, Peter B, Sanchez-Roige, Sandra, Madole, James W, Driver, Morgan N, . . . Dick, Danielle M. . (2021). Multivariate analysis of 1.5 million people identifies genetic associations with traits related to self-regulation and addiction. *Nature neuroscience, 24*(10), 1367-1376.

Mills, Melinda C, Tropf, Felix C, Brazel, David M, van Zuydam, Natalie, Vaez, Ahmad, & Team, BIOS Consortium Management. (2021). Identification of 371 genetic variants for age at first sex and birth linked to externalising behaviour. *Nature human behaviour, 5*(12), 1717-1730.

Nagel, Mats, Jansen, Philip R, Stringer, Sven, Watanabe, Kyoko, De Leeuw, Christiaan A, Bryois, Julien, . . . Muñoz-Manchado, Ana B. (2018). Meta-analysis of genome-wide association studies for neuroticism in 449,484 individuals identifies novel genetic loci and pathways. *Nature genetics, 50*(7), 920-927.

Otowa, Takeshi, Hek, Karin, Lee, Minyoung, Byrne, Enda M, Mirza, Saira S, Nivard, Michel G, . . . Wolen, Aaron. (2016). Meta-analysis of genome-wide association studies of anxiety disorders. *Molecular psychiatry, 21*(10), 1391-1399.

Pulit, Sara L, Stoneman, Charli, Morris, Andrew P, Wood, Andrew R, Glastonbury, Craig A, Tyrrell, Jessica, . . . Ji, Yingjie. (2019). Meta-analysis of genome-wide association studies for body fat distribution in 694 649 individuals of European ancestry. *Human molecular genetics, 28*(1), 166-174.

Tielbeek, Jorim J, Johansson, Ada, Polderman, Tinca JC, Rautiainen, Marja-Riitta, Jansen, Philip, Taylor, Michelle, . . . Tiemeier, Henning. (2017). Genome-wide association studies of a broad spectrum of antisocial behavior. *JAMA psychiatry, 74*(12), 1242-1250.

Van den Berg, Stéphanie M, de Moor, Marleen HM, Verweij, Karin JH, Krueger, Robert F, Luciano, Michelle, Arias Vasquez, Alejandro, . . . Amin, Najaf. (2016). Meta-analysis of genome-wide association studies for extraversion: findings from the genetics of personality consortium. *Behavior genetics, 46*, 170-182.

Verbanck, Marie, Chen, Chia-Yen, Neale, Benjamin, & Do, Ron. (2018). Detection of widespread horizontal pleiotropy in causal relationships inferred from Mendelian randomization between complex traits and diseases. *Nature genetics, 50*(5), 693-698.

Walters, Raymond K, Polimanti, Renato, Johnson, Emma C, McClintick, Jeanette N, Adams, Mark J, Adkins, Amy E, . . . Bertelsen, Sarah. (2018). Transancestral GWAS of alcohol dependence reveals common genetic underpinnings with psychiatric disorders. *Nature neuroscience, 21*(12), 1656-1669.

Williams, Camille M, Poore, Holly, Tanksley, Peter T, Kweon, Hyeokmoon, Courchesne-Krak, Natasia S, Londono-Correa, Diego, . . . Waldman, Irwin D. (2023). Guidelines for evaluating the comparability of down-sampled GWAS summary statistics. *Behavior Genetics, 53*(5), 404-415.

Wray, Naomi R, Ripke, Stephan, Mattheisen, Manuel, Trzaskowski, Maciej, Byrne, Enda M, Abdellaoui, Abdel, . . . Andlauer, Till MF. (2018). Genome-wide association analyses identify 44 risk variants and refine the genetic architecture of major depression. *Nature genetics, 50*(5), 668-681.

Yavorska, Olena O, & Burgess, Stephen. (2017). MendelianRandomization: an R package for performing Mendelian randomization analyses using summarized data. *International journal of epidemiology, 46*(6), 1734-1739.

Zhao, Qingyuan, Wang, Jingshu, Hemani, Gibran, Bowden, Jack, & Small, Dylan S. (2020). Statistical inference in two-sample summary-data Mendelian randomization using robust adjusted profile score.

Zhu, Zhihong, Zhang, Futao, Hu, Han, Bakshi, Andrew, Robinson, Matthew R, Powell, Joseph E, . . . Visscher, Peter M. (2016). Integration of summary data from GWAS and eQTL studies predicts complex trait gene targets. *Nature genetics, 48*(5), 481-487.
